# Serendipitous and Systematic Chemoproteomic Discovery of MBLAC2, HINT1, and NME1‑4 Inhibitors from Histone Deacetylase-Targeting Pharmacophores

**DOI:** 10.1021/acschembio.5c00108

**Published:** 2025-05-08

**Authors:** Severin Lechner, Shuyao Sha, Jigar Paras Sethiya, Patrycja Szczupak, Rafal Dolot, Santosh Lomada, Amirhossein Sakhteman, Johanna Tushaus, Polina Prokofeva, Michael Krauss, Ferdinand Breu, Katharina Vögerl, Martin Morgenstern, German Mouse Clinic Consortium, Martin Hrabě de Angelis, Volker Haucke, Thomas Wieland, Carston Wagner, Guillaume Médard, Franz Bracher, Bernhard Kuster

**Affiliations:** † Chair of Proteomics and Bioanalytics, TUM School of Life Sciences, 9184 Technical University of Munich, Freising 85354, Germany; ‡ Department of Medicinal Chemistry, University of Minnesota College of Pharmacy, Minneapolis, Minnesota 55414, United States; ∥ Division of Bioorganic Chemistry, Centre of Molecular and Macromolecular Studies, Polish Academy of Sciences, Łódź 90-363, Poland; ⊥ Experimental Pharmacology Mannheim, European Center for Angioscience, Medical Faculty Mannheim, Heidelberg University, Ludolf-Krehl-Str. 13−17, Mannheim 68167, Germany; # Department of Biology, Chemistry, Pharmacy, Leibniz Institute fur Molecular Pharmacologie, Robert-Roessle-Strasse 10, Berlin 13125, Germany; ∇ Department of Pharmacy, Center for Drug Research, Ludwig-Maximilians University Munich, Munich 81377, Germany; ○ German Mouse Clinic, Helmholtz Zentrum München, German Research Center for Environmental Health, Institute of Experimental Genetics, Neuherberg 85764, Germany; ◆ Chair of Experimental Genetics, TUM School of Life Sciences, Technische Universität München, Freising 85354, Germany; ¶ German Center for Diabetes Research (DZD), Neuherberg 85764, Germany

## Abstract

Metalloenzyme inhibitors often incorporate a hydroxamic acid moiety to bind the bivalent metal ion cofactor within the enzyme’s active site. Recently, inhibitors of Zn^2+^-dependent histone deacetylases (HDACs), including clinically advanced drugs, have been identified as potent inhibitors of the metalloenzyme MBLAC2. However, selective chemical probes for MBLAC2, which are essential for studying its inhibitory effects, have not yet been reported. To discover highly selective MBLAC2 inhibitors, we conducted chemoproteomic target deconvolution and selectivity profiling of a library of hydroxamic acid-type molecules and other metal-chelating compounds. This screen revealed MBLAC2 as a frequent off-target of supposedly selective HDAC inhibitors, including the HDAC6 inhibitor SW-100. Profiling a focused library of SW-100-related phenylhydroxamic acids led to identifying two compounds, KV-65 and KV-79, which exhibit nanomolar binding affinity for MBLAC2 and over 60-fold selectivity compared to HDACs. Interestingly, some phenylhydroxamic acids were found to bind additional off-targets. We identified KV-30 as the first drug-like inhibitor of the histidine triad nucleotide-binding protein HINT1 and confirmed its mode of inhibition through a cocrystal structure analysis. Furthermore, we report the discovery of the first inhibitors for the undrugged nucleoside diphosphate kinases NME1, NME2, NME3, and NME4. Overall, this study maps the target and off-target landscape of 53 metalloenzyme inhibitors, providing the first selective MBLAC2 inhibitors. Additionally, the discovery of pharmacophores for NME1-4 and HINT1 establishes a foundation for the future design of potent and selective inhibitors for these targets.

## Graphical abstract

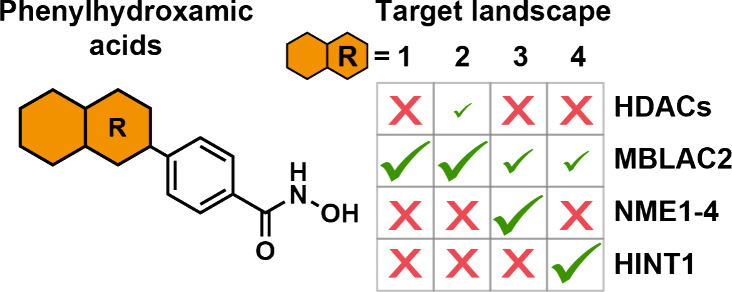

## Introduction

The hydroxamic acid motif is a functional group of small molecule drugs that binds to active site metal ions, such as Zn^2+^ cofactors in matrix metalloproteases or histone deacetylases.^1^ In a prior study, we unexpectedly found that a large fraction of hydroxamic acid–based HDAC inhibitors (HDACis) also bind and inhibit MBLAC2 as an off-target.^2^ Among those molecules were clinically advanced (Pracinostat, AR-42/REC-2282, in phase 3) or approved drugs (e.g., Panobinostat), as well as frequently used HDAC6-targeting chemical probes such as Tubastatin A, Nexturastat A, and Tubacin. There is not much known about the cellular function of MBLAC2. However, recombinant MBLAC2 hydrolyses acyl-CoA into the free fatty acid and CoA in vitro.^3^ MBLAC2 has also been shown to interact with the acyl-transferase ZDHHC20 ^3,4^ and other membrane-associated proteins, such as SLC9A6, which play roles in vesicle generation or endosomal biology.^4,5^ Knockdown or pharmacological inhibition of MBLAC2 substantially increases the number of extracellular vesicles in HEK293 cell culture and remodels the lipidome.^2^ These findings place MBLAC2 into the functional context of membrane-associated processes related to the endosome and lysosome, which eventually influence the secretion or uptake of extracellular vesicles (EVs).^2^ Considering the diverse physiological roles of EVs in cancerous and neurological diseases,^6^ a better understanding of MBLAC2 is desirable to recognize whether the inactivation of MBLAC2 via clinical HDACis might cause favorable or adverse effects.

Here, we report on the phenotypic characterization of a newly generated MBLAC2 knockout mouse model and the chemoproteomic characterization of 53 metal-chelating metalloenzyme inhibitors with the aim to providing biological and pharmacological tools to study MBLAC2 function. Surprisingly, the knockout mouse did not show obvious adverse phenotypes but gratifyingly, the chemoproteomic screen discovered highly selective MBLAC2 inhibitors. To our surprise, some of the profiled phenylhydroxamic acids bound to additional proteins, notably the nucleoside diphosphate kinases NME1–4 and the histidine triad nucleotide-binding proteins (HINT1–2). We further explored this serendipitous discovery by enzyme activity assays, molecular docking, and crystallography studies to demonstrate binding as well as inhibition of these off-targets. As a result, this study reports the first highly selective MBLAC2 inhibitors and the first drug-like small molecule inhibitors for two entirely unrelated enzyme families that may be further exploited in the future.

## Results

### MBLAC2 Is Apparently Expendable for Healthy Mouse Physiology

To investigate MBLAC2-associated phenotypes at a systems level, we examined whether genetic MBLAC2 inactivation would produce a phenotypic fingerprint indicative of MBLAC2 function. In collaboration with the International Mouse Phenotyping Consortium (IMPC) pipeline, we created an MBLAC2 knockout (KO) mouse model. Eight female and 10 male KO animals and 11 female and 13 male WT animals of age 8–16 weeks were subjected to testing of >70 clinical and physiological parameters including behavior, neurology, cardiovascular function, morphology, immunology, pathology, and clinical chemistry.^7–9^ MBLAC2 KO mice developed normally, with no substantial physiological differences observed across the >70 tested phenotypes. This data is publicly accessible via the Mouse Clinic Phenomap Viewer (https://tools.mouseclinic.de/phenomap/jsp/annotation/public/phenomap.jsf). Beyond the IMPC phenotyping pipeline, we conducted proteomic analyses of fresh-frozen brains from KO and WT mice. We identified and quantified over 8,000 proteins across all 12 brain samples from female and male WT and KO animals (n = 3 animals per group). This analysis revealed no substantial proteomic differences between KO and WT mice, except for the expected absence of MBLAC2 in the KO group (Figure S1). In summary, these findings suggest that MBLAC2 function is dispensable for maintaining normal physiology in healthy mice under the tested conditions.

### HDAC Inhibitor Selectivity Profiling Reveals Novel MBLAC2 Binders

Since the MBLAC2 KO mouse model did not provide new insights into the biological functions of MBLAC2, we shifted attention to identifying chemical tools that can inhibit MBLAC2 activity *in vitro*. MBLAC2 is a metalloenzyme predicted to harbor one or two Zn^2+^ ions in its active site.^3^ To identify novel pharmacophores for designing selective MBLAC2 inhibitors, we screened 23 metal-chelating molecules, including 12 HDAC inhibitors (Figure S2). We employed a chemoproteomic competition assay, which we had previously used to profile the target space and selectivity of HDAC and MBLAC2 inhibitors.^2^ In this assay, compounds of interest are incubated at increasing concentrations with a mixture of lysates of MV4–11 and SW620 cells. The compound-treated lysates are then mixed with bead-immobilized hydroxamic acid-containing compounds iA, iC, and iQ (immobilized Quisinostat),^2^ which pull down compound-binding proteins such as HDACs, MBLAC2, ISOC1/2, ALDH2, and GATD3A (Figure 1a) that can be identified and quantified by mass spectrometry (MS). Proteins targeted by the library compounds in a preincubation step are prevented from being pulled down by the immobilized probes, leading to a dose-dependent reduction in MS signal. Initially, 12 metal-chelating compounds were profiled at two concentrations (10 μM and 100 μM) (Figure S3). Two hit compounds showing target engagement in this experiment were subsequently tested, along with 12 reported HDAC inhibitors, in a nine-dose competition assay. The resulting compound selectivity data provides a significant update to the target landscape of HDAC inhibitors previously published by the authors^2^ and revealing several noteworthy findings (Figure 1b, Table S1). For instance, Tinostamustine and SBHA (suberoyl-bis-hydroxamic acid) were found to bind off-targets such as ISOC1/2, GATD3A, and ALDH2. Notably, ISOC1 was selectively targeted by SKLB-23bb with an EC_50_ of 5.6 μM, while Ibuproxam bound ALDH2 and GATD3A with EC_50_ values in the range of 23–30 μM. These findings align with previously observed off-target binding of structurally related small-molecule drugs (Figure S4).^2^

**Figure 1:**
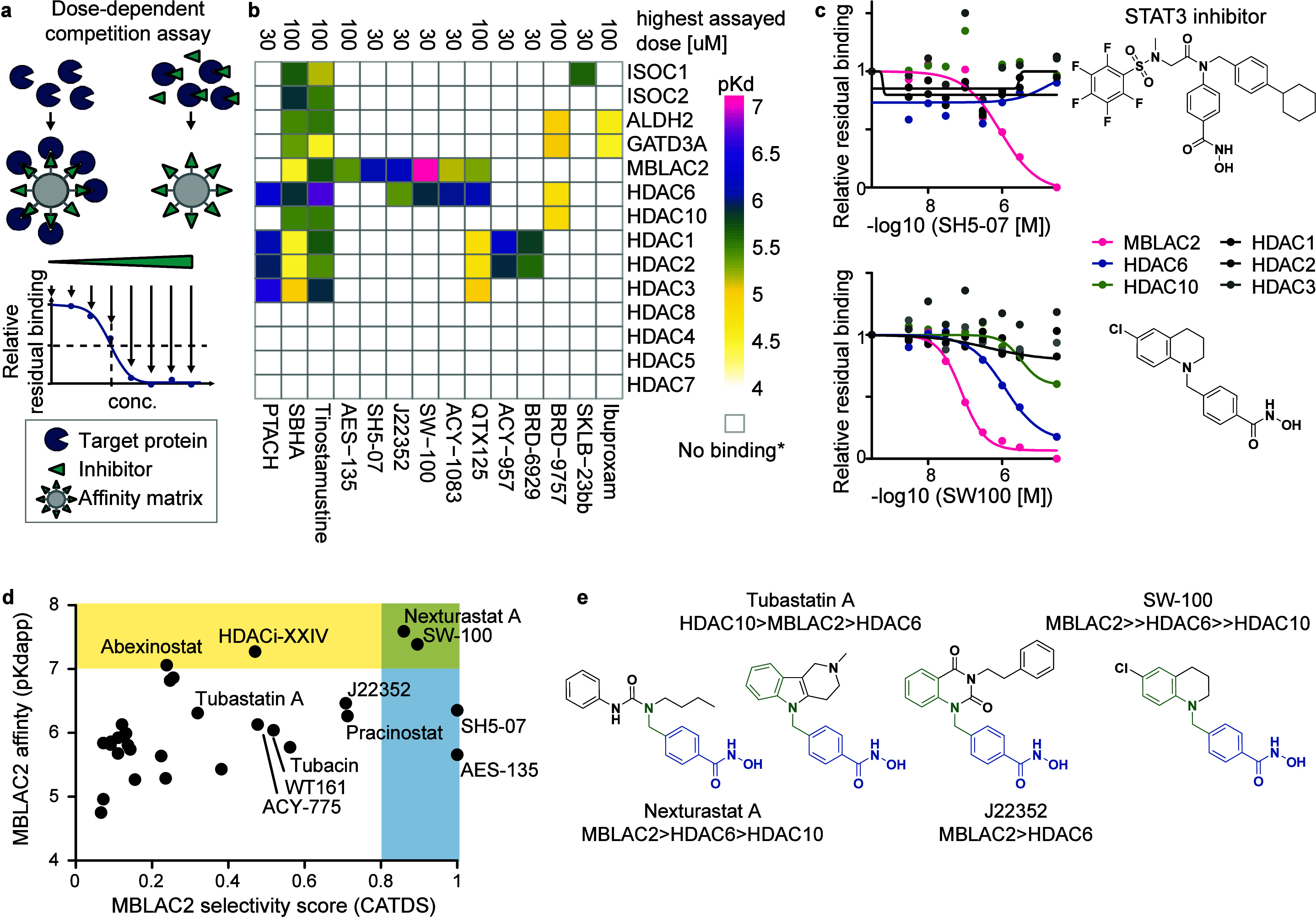
Chemoproteomic profiling identifies MBLAC2 binders among metalloenzyme inhibitors. (a) Schematic of the chemoproteomic competition assay used to screen for MBLAC2 binding compounds in 2-dose or 9-dose formats. (b) Heatmap of compound-target affinities (p*K*_D_^app^ values) from 9-dose profiling. White spaces indicate >50% residual binding at the highest tested concentrations (30 μM or 100 μM), i.e. p*K*_D_^app^ values higher than 30 μM or 100 μM. (c) Dose–response curves for SH5–07 (a proposed STAT3 inhibitor) and SW-100 (a purportedly selective HDAC6 inhibitor). (d) Drug affinity (p*K*_D_^app^) versus MBLAC2 selectivity (CATDS) score, highlighting potent and selective binders (CATDS > 0.8, p*K*_D_^app^ > 7). Combined data from this study and a previous chemoproteomic profiling with the same workflow.^2^ (e) Structures of select MBLAC2 binders, which show submicromolar MBLAC2 affinity and medium-to-high selectivity. Key pharmacophore features include the phenylhydroxamic acid (blue) and frequently an aromatic capping group attached in *para*-position via an aminomethyl unit (green). The order of target affinity is indicated for each drug.

In our previous survey of the HDAC inhibitor (HDACi) target space, more than 50% of hydroxamic acid-type compounds were found to bind and inhibit MBLAC2.^2^ Consistently, in this study, half of the 14 hydroxamic acid compounds tested in the nine-dose profiling assay bound to MBLAC2. Notably, AES-135, a proposed pan-HDAC inhibitor,^10^ displayed no HDAC target engagement at concentrations up to 30 μM but selectively bound to MBLAC2 with an apparent dissociation constant (K_D_^app^) of approximately 3.5 μM. Similarly, SH5–07, a proposed STAT3 inhibitor with high structural similarity to AES-135,^10,11^ engaged MBLAC2 with a K_D_^app^ of ∼ 800 nM (Figure 1c). Additionally, the purportedly selective HDAC6 inhibitors ACY-1083,^12^ J22352,^13^ and SW-100 ^14^ potently bound MBLAC2, raising concerns about their suitability as HDAC6 chemical probes (Figure 1b,c). These findings further highlight the potential significance of MBLAC2 as a frequent off-target of HDAC inhibitors.^2^ To identify potential starting points for developing selective MBLAC2 chemical probes, we calculated the concentration- and target-dependent selectivity (CATDS) score^15^ for all MBLAC2 binders identified in this and previous proteomic profiling campaigns^2^ (Fig. 1d). The CATDS score quantifies the extent of drug-target engagement at a specific concentration by comparing the engagement of a target of interest at its half-maximal binding concentration (K_D_^app^) to the total target engagement across all targets at the same concentration.^15^ The analysis revealed that compounds with low affinity for HDACs but potent MBLAC2 binding often share a phenylhydroxamic acid pharmacophore, frequently extended by a capping group attached in *para*-position via an aminomethyl unit (Fig. 1d-e). The four compounds AES-135, SH5–07, SW-100, and Nexturastat A (REF^2^) demonstrated highest selectivity for MBLAC2 over HDAC6, offering templates for designing highly selective MBLAC2 inhibitors. AES-135 and SH5–07, however, seem less favorable for MBLAC2 chemical probe development due to their origin as STAT3 inhibitor derivatives, which may introduce STAT3 off-target effects. Among the selective MBLAC2 binders, SW-100 stood out, showing approximately 25-fold selectivity for MBLAC2 over HDAC6 (K_D_^app^ [MBLAC2] = 75 nM, K_D_^app^ [HDAC6] = 1.2 μM). While Nexturastat A additionally binds HDAC10, SW-100, which is a compound structurally related to the HDAC10 and MBLAC2 inhibitor Tubastatin A,^2^ appears to have lost its affinity for HDAC10 (Fig. 1c). Based on these findings, we selected SW-100 as a template for designing selective MBLAC2 inhibitors.

### Chemoproteomic Structure-Affinity Relationship (SAR) Analysis Identifies MBLAC2 Chemical Probe Candidates

To find MBLAC2 inhibitors with enhanced selectivity, we synthesized a focused library of 27 phenylhydroxamic acid derivatives structurally related to SW-100 (Figure 2, Table S2). Some of these compounds were previously reported to exhibit low HDAC6 affinity,^16^ increasing the likelihood of repurposing molecules that had lost HDAC binding affinity while retaining strong MBLAC2 affinity. The series also included analogs with alternative metal-chelating moieties, such as thiohydrazide (MM7), carboxyanilide (MM21), and thiourea (KV-92), to explore the potential for achieving MBLAC2 selectivity through novel Zn^2+^-chelating warheads. We also included a compound with an extended linker region between the zinc-binding and capping groups (KV-176). This 27-compound library was analyzed for proteome-wide target binding using a two-dose (1 μM and 10 μM) chemoproteomic competition assay (Figure S5) to estimate MBLAC2 versus HDAC binding affinities. Subsequently, nine candidates were subjected to a full dose–response (nine doses) assay for validation. As expected, at 10 μM, the SW-100 analogs exhibited no binding to HDAC targets or off-targets other than HDAC6 and MBLAC2. The structure-affinity relationship (SAR) findings for HDAC6 and MBLAC2 are summarized in Figure 2 and Table S3.

**Figure 2:**
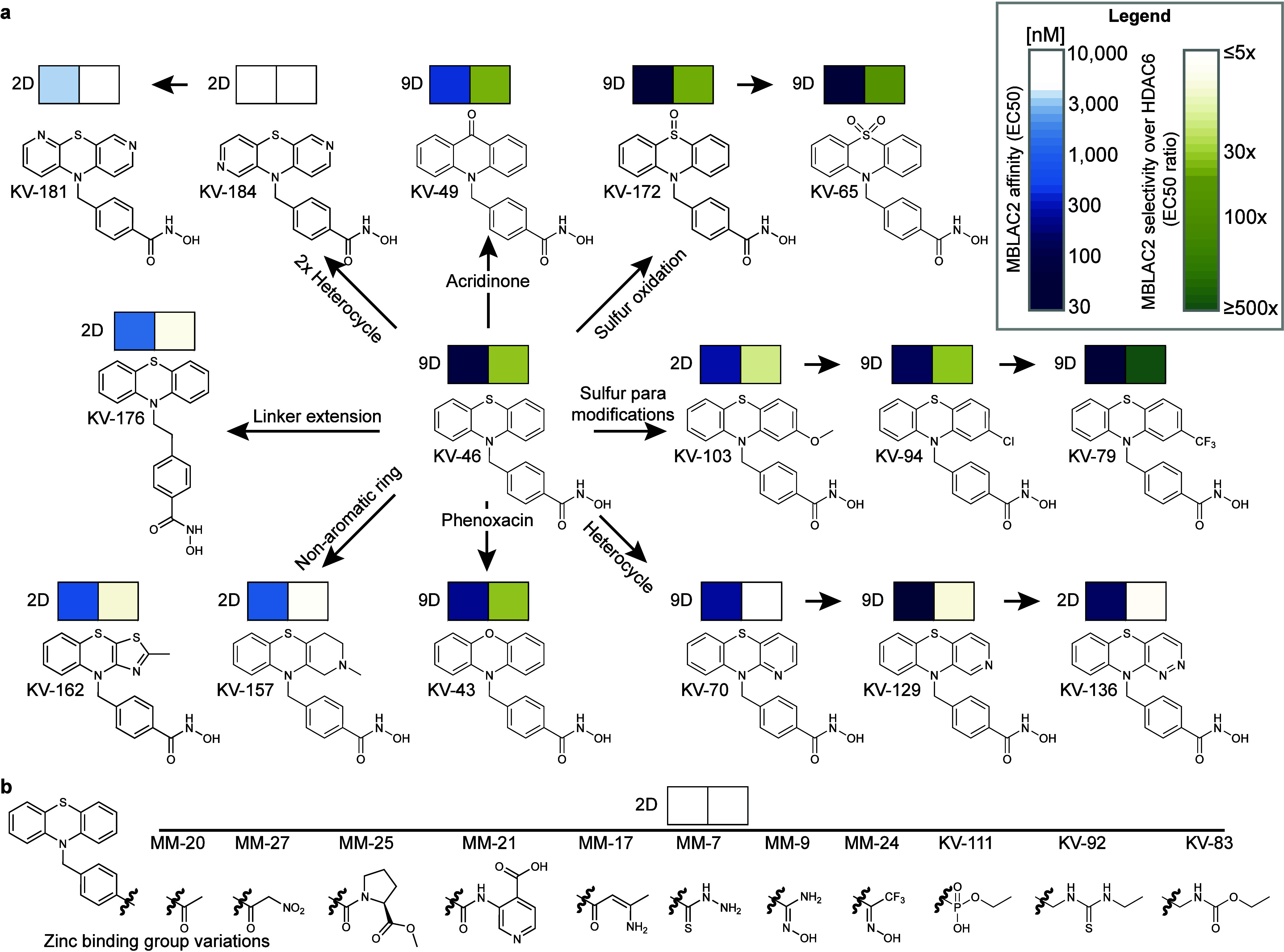
Structure–activity relationship (SAR) analysis of an MBLAC2 directed compound library. (a) Overview of MBLAC2 and HDAC6 inhibitors featuring hydroxamic acid moieties. Binding affinity (EC_50_) for MBLAC2 and selectivity over HDAC6 (EC_50_^HDAC6^/ EC_50_^MBLAC2^) are color-coded: high affinity (<100 nM, dark blue) and >30-fold selectivity (light to dark green) are key criteria for chemical probes. Data were derived from 2-dose (2D) or 9-dose (9D) profiling in chemoproteomic competition assays. For compounds with no detectable HDAC6 binding at the highest assayed concentration (30 μM), this threshold was used to calculate selectivity, potentially underestimating the value. Compounds are grouped by chemical features. (b) SAR of compounds with alternative zinc-chelating moieties. None of the compounds bound to MBLAC2 or HDAC6.

Phenothiazine-based KV-46,^16^ which served as the central pharmacophore for library diversification, displayed high potency for MBLAC2 (EC_50_= 83 nM) and more than 30-fold selectivity over HDAC6 (EC_50_ = 2.75 μM). Modifications such as introducing heterocycles, extending the linker region, or replacing one of the phenyl rings with a nonaromatic moiety typically reduced MBLAC2 selectivity by either increasing HDAC6 affinity, decreasing MBLAC2 affinity, or both. However, several compounds demonstrated improved selectivity for MBLAC2 compared to both SW-100 and the parent phenothiazine KV-46. For instance, modifications to the phenyl ring in the para position relative to the sulfur group yielded compounds with high MBLAC2 affinity and selectivity, such as KV-79 (EC_50_ = 25 nM). Interestingly, oxidation of the sulfur in sulfoxide KV-172 and sulfone KV-65 increased MBLAC2 affinity. KV-65 demonstrated an EC_50_ of 37 nM and 64-fold selectivity over HDAC6 (EC_50_ = 2.4 μM), meeting the criteria for a chemical probe with over 30-fold selectivity^17^ (Figure S6a). Notably, compounds featuring nonhydroxamic acid metal-chelating warheads lost binding affinity to both HDAC6 and MBLAC2 (Figure 2b). For example, the carbonyl group in MM-20, which is sterically less demanding than hydroxamic acid, showed no binding at concentrations up to 10 μM. This finding underscores the critical role of the hydroxamic acid-metal interaction in driving overall binding affinity (Figure 2b).

Based on the SAR data, we propose KV-79 and KV-65 as probes for MBLAC2. Consistent with previous findings that MBLAC2 inhibition or knockdown is not cytotoxic or cytostatic,^2^ treatment of cells with the selective MBLAC2 inhibitors KV-65 and KV-79 did not affect cell proliferation or fitness at concentrations up to 10 μM (Figure S6b). Our earlier observation that MBLAC2 inactivation leads to the upregulation of extracellular vesicles (EVs),^2^ combined with evidence of MBLAC2’s association with late endosomes and membrane processes,^2,5^ prompted us to hypothesize that MBLAC2 inactivation might impair endocytosis. To test this hypothesis, we measured clathrin-mediated endocytosis of fluorescently labeled transferrin (TF) in the presence and absence of MBLAC2 inhibitors. While MBLAC2 inhibitors showed a trend toward reducing TF uptake, the results were not statistically significant (Figure S6c). Thus, the precise biological roles of MBLAC2 remain elusive. However, we anticipate that the selective compounds identified in this study will serve as valuable tools for elucidating the cell biological functions of MBLAC2 in future research.

### Phenylhydroxamic Acids Also Bind NME and HINT Enzymes

Our initial screening library included three additional hydroxamic acids with 4-(heteroaryl)-phenyl motifs: KV-24, KV-30, and KV-50, which were proteomically characterized alongside the SW-100 analogs. While these compounds initially appeared to demonstrate excellent selectivity for MBLAC2 over HDAC6, we unexpectedly observed dose-dependent competition of several other proteins in chemoproteomic competition experiments (Figure 3a). These compounds also bound to HINT1 and NME4, and, with lower affinity, HINT2 and a protein ambiguously identified as NME1 or NME2 (Figure 3a,b). Since HINT and NME enzymes are not classified as canonical metalloproteins, their binding to hydroxamic acids was very surprising. We, therefore, investigated the mechanism underlying the observed binding in more detail. Analyzing published pulldown data^2^ revealed that immobilized Quisinostat (iQ) (Figure S7a) is the affinity probe responsible for the pulldown of HINTs and NMEs, with HINT1 actually being the most abundant protein in iQ pulldown data sets. Additionally, HINT2 and NMEs were identified in iQ pulldowns at intensity levels comparable to the designated HDAC targets of Quisinostat (Figure S7b). Notably, prior competition experiments with Quisinostat and iQ did not indicate binding of free Quisinostat to HINTs or NMEs^2^ which is why these target proteins went unnoticed. These observations suggest that the acylation of Quisinostat during the immobilization on the matrix plays a significant role in enhancing the binding affinity to these proteins. Of note, HINT1 and HINT2 have previously been pulled down by an affinity matrix containing a trifluoromethyloxadiazole zinc-binding group in chemoproteomic profiling of class IIa HDAC inhibitors.^18^ Even though our initial goal was to merely identify MBLAC2 inhibitors, the serendipitous discovery of potential ligands for HINT1 and an enzyme of the yet undrugged NME kinase family prompted us to further explore these unexpected small molecule-protein interactions.

**Figure 3:**
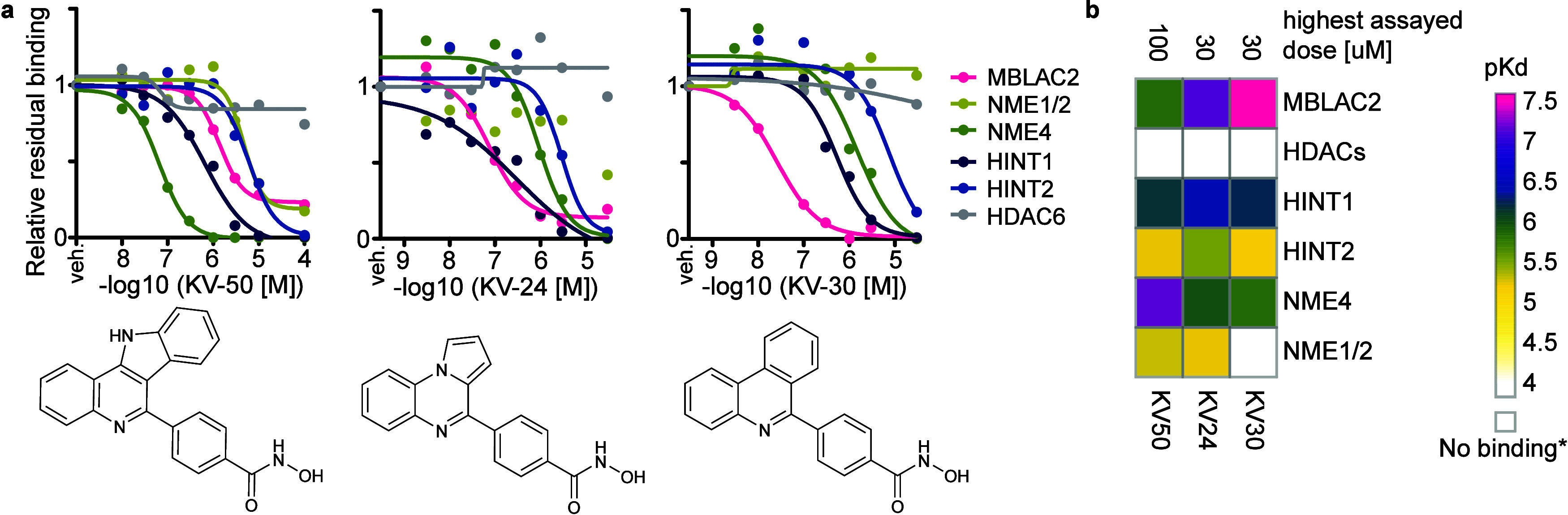
Binding of phenylhydroxamic acids KV-50, KV-24, and KV-30 to HINT and NME enzymes. (a) Dose–response curves for KV-50, KV-30, and KV-24 target proteins, as determined in chemoproteomic competition experiments. (b) Summary of the target space and selectivity profiles of KV-50, KV-30, and KV-24, derived from dose–response data.

### KV-24 and KV-30 Inhibit HINT1

Histidine triad nucleotide-binding proteins (HINTs) exhibit hydrolytic activity toward nucleoside phosphoramidates, such as tryptamine adenosine phosphoramidate (TrpA) (Figure 4a).^19^ While the biologically relevant substrates of HINT1 have not been clearly elucidated, the protein has been implicated in opioid receptor signaling,^20–22^ regulation of the melanoma-associated transcription factor MITF,^23,24^ and DNA damage repair.^25^ These functions position HINT1 as a potential pharmacological target for treating conditions such as opioid resistance or melanoma. The only reported HINT1-targeting pharmacophores are nucleoside-based nonhydrolyzable substrate analogs, such as the carbamate TrpGC (Figure 4a).^19,20,22^ We evaluated the inhibitory potential of KV-24 and KV-30 in enzyme activity assays using recombinant HINT1. Both compounds effectively inhibited HINT1 activity, with half-maximal inhibitory concentrations (IC50) of 12.3 μM for KV-24 and 12.6 μM for KV-30, which are comparable to the state-of-the-art HINT1 inhibitor TrpGC (IC50 = 9.1 μM) (Figure 4b). Notably, the IC50 values were approximately 1 order of magnitude higher than the EC_50_ values determined in lysate-based assays. This discrepancy could be attributed to reduced affinity for the recombinant enzyme due to the absence of post-translational modifications (PTMs), cofactors, or interaction partners that enhance drug binding. Alternatively, it might result from substrate dilution in the lysate, whereas high substrate concentrations in the enzyme activity assay require the inhibitor to compete more effectively.

**Figure 4:**
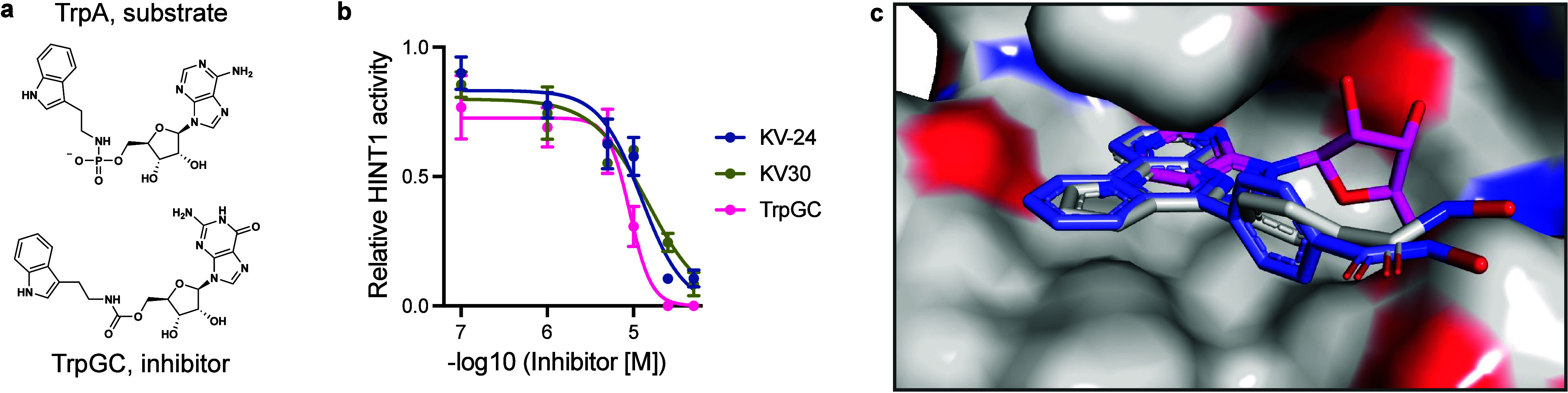
KV-24 and KV-30 bind and inhibit HINT1. (a) Structures of reported HINT1 substrate TrpA and HINT1 substrate analog HINT1 inhibitor TrpGC. (b) HINT1 enzyme activity assay showing inhibition by KV-24, KV-30, and the state-of-the-art HINT1 inhibitor TrpGC. TrpA is the HINT1 substrate. (c) Co-crystal structures of KV-24 (gray) and KV-30 (blue) bound to the nucelotide-binding pocket of HINT1. Structures determined here are overlaid with the AMP-bound (pink) HINT1 structure (PDB: 3TW2).

To investigate the inhibitory mechanism further, we solved cocrystal structures of HINT1 bound to KV-24 and KV-30 (PDB: 9GYP and 9GYQ) (Tables S4 and S5). The structures revealed that the aromatic ring systems of KV-24 and KV-30 are buried in a hydrophobic cleft typically occupied by the base of the substrate nucleoside (Figure 4c).^23^ This finding supports a competitive inhibition mode of action.

The binding of KV-24 and KV-30 to the nucleotide-binding pocket raises the question of whether these compounds also target other nucleotide-binding proteins, such as kinases. To evaluate potential off-target effects, we performed Kinobead competition assays. This chemoproteomic approach is conceptually very similar to the one used here in that broad spectrum kinase inhibitors are immobilized on beads. Kinobeads can assess compound binding to ∼ 200 human kinases and other kinase inhibitor off-targets (e.g., FECH and NQO2).^26,27^ Among the >190 kinases robustly quantified in our experiments, MERTK was the only kinase showing potential binding to KV-30 (Figure S8a). Additionally, casein kinase 2 family members exhibited reduced binding at two-digit μM concentrations of KV-24 (Figure S8a). Interestingly, KV-24 showed dose-dependent binding to calcium/calmodulin-dependent 3′,5′-cyclic nucleotide phosphodiesterase 1C (PDE1C), with an affinity of approximately 2 μM (Figure S8a,b). PDE1C is a cAMP/cGMP nucleotide-binding protein that features two divalent metal cation cofactors.^28^ The hydroxamic acid moiety of KV-24 might chelate these metal cofactors, supporting the hypothesis that KV-24 binds the active site of PDE1C. Thus, KV-24 and KV-30 should be regarded only as preliminary scaffolds for developing more selective inhibitors. With the help of our chemoproteomic platform, future structure–activity relationship (SAR) libraries based on the KV-24 and KV-30 scaffolds may be explored to improve selectivity toward either HINT1, kinase targets, or PDE1C.

### KV-50 Is a Pan-NME Inhibitor

Nucleoside diphosphate kinases (NMEs) are multifunctional proteins that assemble into hexamers^29–31^ and catalyze the formation of nucleoside triphosphates (NTPs) from nucleoside diphosphates.^31^ As such, for instance, NME1 and NME2 locally generate GTP to fuel dynamins during membrane remodeling and endocytosis.^32^ The inactivation of NME1 or NME2 has been shown to impair endocytosis.^32^ Beyond their cytoplasmic roles, NME2 has also been implicated in transcriptional regulation, such as at the MYC locus^33,34^ and NME1 has been linked to DNA damage repair.^35–37^ Both NME1 and NME2 contribute to metabolism-guided epigenetic gene regulation.^38–40^ NME3 is localized at the mitochondrial outer membrane and, for instance, has been linked to regulation of hypoxia-induced mitophagy.^41^ NME4 localizes to the mitochondrial intermembrane space and impacts the organelle’s biology via NTP generation and additional functions in cardiolipin and phospholipid transfer.^30^

To date, no inhibitor of NME enzyme activity has been reported. To evaluate whether KV-50 binding affects NME activity, we performed enzyme activity assays using recombinant NME1–4. The assay measured NME-catalyzed production of ATP from ADP (150 μM) and GTP (150 μM). The results showed that KV-50 inhibited all four enzymes, NME1–4, with EC_50_ values ranging from 2 to 5 μM (Figure 5a). Increasing the substrate concentration in these assays abolished KV-50-mediated inhibition, indicating a competitive binding inhibition mode (Figure S9a). This observation could explain the reduced potency of KV-50 in activity assays compared to chemoproteomic assays, as the latter involved lysates with diluted nucleotide pools. Compounds KV-24 and KV-30, which showed lower affinity to NME4 in chemoproteomic assays, only modestly reduced NME enzyme activity in vitro (Figure S9b). The diminished activity of the compounds in enzyme assays may also result from the absence of post-translational modifications (PTMs), cofactors, or interaction partners present in lysates, which might enhance binding affinity but are not replicated in the in vitro assay.

**Figure 5:**
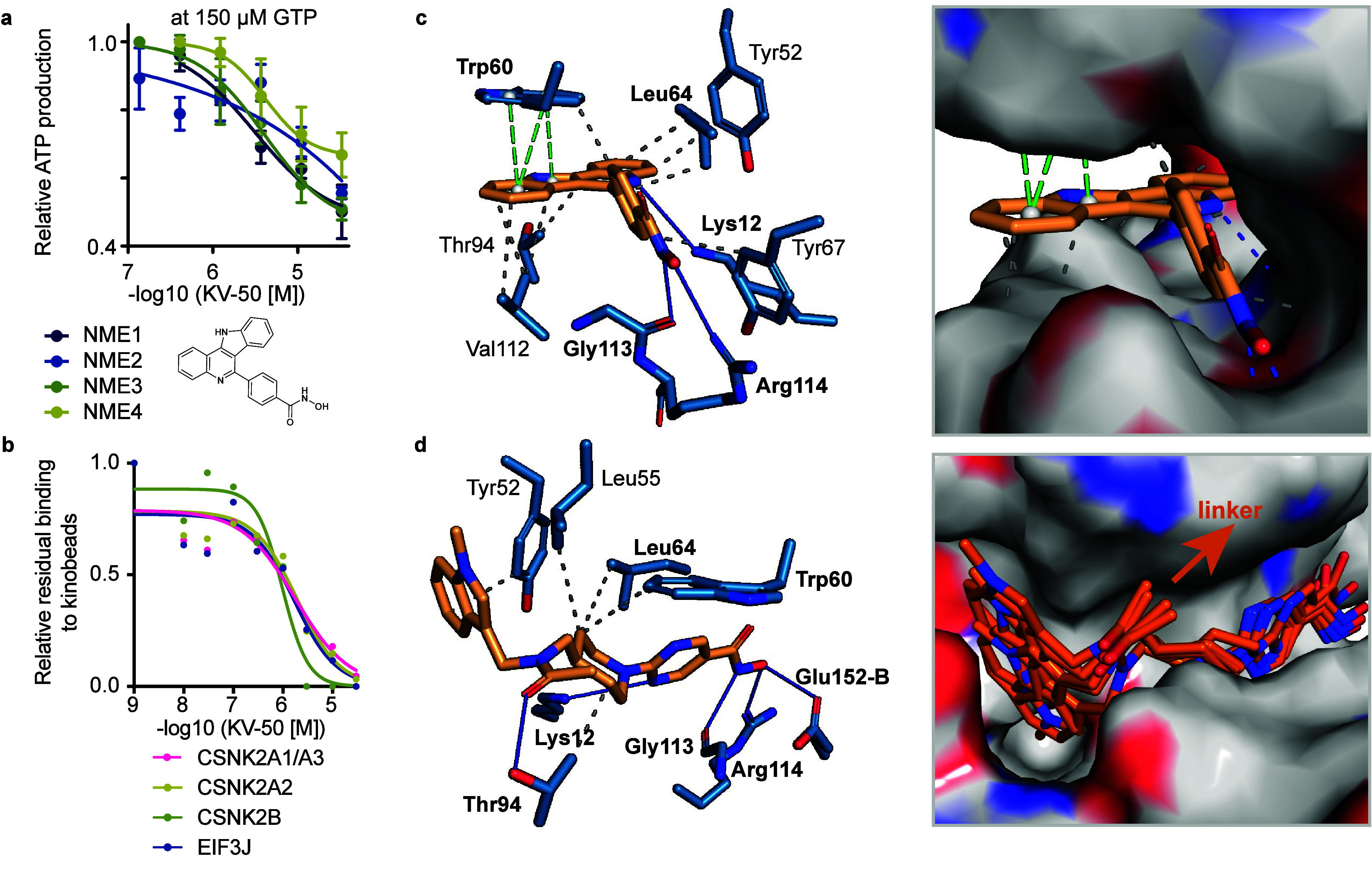
KV-50 is a pan-NME inhibitor. (a) Inhibition of NME1–4 enzyme activity by KV-50, measured via nucleoside-diphosphate kinase-catalyzed production of ATP from ADP and GTP. (b) Competition curves for casein kinase 2 catalytic subunits and interactors, as determined by the Kinobeads assay. (c) Docking pose of KV-50 in the hydrophobic cleft of NME1. (d) Docking pose of acetylated Quisinostat, modeled as a surrogate for the immobilized Quisinostat affinity matrix iQ, which consists of Quisinostat covalently linked to beads via acylation of its secondary amine (see also Figure S7a). Left: Key interactions between acetylated Quisinostat and NME1. Right: The top five docking poses show a consistent orientation, with the acetylated secondary amino group exposed to the solvent. Interactions were modeled with the PLIP tool: gray lines indicate van der Waals interactions, blue lines denote hydrogen bonds, and green lines represent π-stacking interactions.

Given that local GTP production by NME1 and NME2 is essential for fueling dynamin-driven endocytosis,^32^ we investigated the effect of KV-50-mediated NME1/2 inhibition on fluorescent transferrin (TF) uptake in cells. HeLa cells were treated with KV-50 or known endocytosis inhibitors (Pitstop-2 and Dynasore), and surface-bound and internalized fluorescent TF were quantified. KV-50-treated cells exhibited a reduced ratio of internalized TF to surface-bound TF (Figure S9c). However, KV-50 also induced morphological changes, such as rounding and swelling of cells under these assay conditions. We made similar observations for SW-620 and HEK293T cells (Figure S9d). Consequently, the observed reduction in TF uptake could not be unequivocally linked to NME1/2-dependent endocytosis inhibition. In addition to morphological changes, KV-50 treatment reduced SW620 cell confluency by 50% at 870 nM and decreased metabolic activity by 50% at 3.6 μM (Figure S9e), aligning with the NME1–4 inhibitory EC_50_ range of 2–5 μM. While the knockout of a single NME enzyme did not significantly impact cell fitness across >900 cancer cell lines in the DepMap project,^42^ pan-inhibition of NME enzymes, which share redundant functions, could be lethal and may explain these observations. Alternatively, additional off-targets of KV-50 might contribute to its cytostatic or cytotoxic effects. Assuming that KV-50 binds to the nucleotide-binding pocket of the NME kinase domain (competitive inhibition), other nucleotide-binding enzymes are potential off-target candidates. To explore this, we again conducted chemoproteomic competition assays with Kinobeads. Among >190 kinases identified, only the casein kinase 2 (CK2) complex proteins (CSNK2A1/3, CSNK2A2, CSNK2B, EIF3J) showed dose-dependent competition, with EC_50_ values in the range of 1–2 μM (Figure 5b). Of note, CK2 was previously annotated as a kinase with exceptionally high affinity to GTP.^43^ CK2 is known to promote cell proliferation and counteract apoptotic signaling.^44^ Thus, CK2 inhibition by KV-50 could also contribute to its observed effects on cell fitness.

Having identified a pharmacophore for the previously undrugged NMEs, we performed docking studies with KV-50 and the active site of NME1 to explore potential binding modes. In the top-ranked docking model, the planar aromatic ring structure of KV-50 is nestled within a hydrophobic cleft, forming π-stacking interactions with a tryptophan side chain (Trp60) (Figure 5c, Figure S9f). Notably, in the cocrystal structure of ADP-bound NME1 (PDB: UCN1), the adenine base of the ADP substrate binds to the same hydrophobic cleft, and the indole moiety of KV-50 overlaps with the adenine base, undergoing similar π-stacking interactions with Trp60 (Figure S9g). The indole moiety of the aromatic capping group, unique to KV-50, likely explains its higher affinity compared to KV-24 and KV-30. In addition to hydrophobic and π-stacking interactions, the docking suggests that the hydroxamic acid of KV-50 forms multiple hydrogen bonds. Specifically, the hydroxamic acid interacts with the backbone carbonyl oxygen of Gly113 and the side chain of Arg114, further stabilizing the binding pose.

Interestingly, docking studies with acetyl-Quisinostat, a surrogate for immobilized Quisinostat, revealed binding poses that also involve hydrogen bonding between the hydroxamic acid and the same amino acid residues as for KV-50 (Figure 5d). In these poses, the pymiridine ring of acetyl-Quisinostat overlaps with the phenyl ring of KV-50 (Figure S9h). Notably, the acetyl group, surrogating the alkyl-chain linker in immobilized Quisinostat (iQ), projects outward from the active site, making this binding mode plausible for the pulldown of NMEs via iQ (Figure S9h). Furthermore, the docking model suggests that the acetyl-oxygen of acetyl-Quisinostat forms a hydrogen bond with Thr94, potentially explaining why iQ binds to NMEs while free nonacetylated Quisinostat does not exhibit binding at concentrations up to 30 μM.^2^ These docking experiments rationalize the critical role of the hydroxamic acid in target binding and provide a framework for future medicinal chemistry efforts to design more potent and selective NME1 inhibitors.

## Discussion and Conclusions

We recently identified MBLAC2 as a frequent off-target of HDAC inhibitors with potential clinical implications.^2^ MBLAC2 knockdown or inhibition does not affect cell viability in HEK293 cells, SW620 colon cancer cells,^2^ or across several hundred cancer cell lines profiled in the DepMap project.^42^ Similarly, we here observed that MBLAC2 knockout (KO) in mice does not significantly impact the physiology of healthy animals. However, caution is warranted when extrapolating these findings to human patients treated with MBLAC2 inhibiting molecules, as (i) human physiology may be different to that of mouse models, (ii) MBLAC2 functions may be relevant in disease contexts not tested in our mouse models, and (iii) mice with a lifelong deletion of MBLAC2 may develop compensatory mechanisms, which would not occur in patients undergoing acute MBLAC2 inhibition with small molecules. Because the genetic depletion of MBLAC2 activity was not informative, we turned to creating selective MBLAC2 inhibitors instead which would allow the study of the immediate effects of MBLAC2 inactivation in human model systems. To identify such probes, we profiled a set of HDAC inhibitors with MBLAC2-targeting potential. Consistent with prior findings,^2^ MBLAC2 was an off-target for half of the hydroxamic acid–based HDACis tested. Additionally, the HDACis Tinostamustine (clinical phase I) and SBHA bound to off-targets such as ISOC1/2, GATD3A, and ALDH2. This aligns with observations that compounds related to Vorinostat (suberoylanilide hydroxamic acid, SAHA) or Ricolinostat (ACY-1215) exhibit similar off-target effects.^2^ A subset of hydroxamic acid-type compounds, including Ibuproxam and Bufexamac (Figure 1 and Figure S4a), also bound ALDH2 and GATD3A. These structurally related compounds are known to cause dermatitis via an unknown mechanism, which could be related to these off-target interactions.^45–47^

Among the compounds investigated for proteome-wide target binding, the phenylhydroxamic acid–based inhibitor SW-100 stood out for its selectivity for MBLAC2. Additional profiling of 30 SW-100-related molecules identified several highly selective MBLAC2 inhibitors. Interestingly, 70% of the tested phenylhydroxamic acids also bound MBLAC2, often with higher affinity than HDACs, suggesting that many reported phenylhydroxamic acids may function as dual MBLAC2/HDAC6 inhibitors—or even exhibit greater potency toward MBLAC2. This finding implies that observed phenotypic effects currently solely attributed to HDAC6 inhibition may also, or instead, result from MBLAC2 binding. For example, SW-100 was recently developed into an ^18^F-labeled PET imaging probe for quantifying HDAC6 expression in the brain^48^ but it likely also quantifies MBLAC2 expression. Another study used three purportedly selective HDAC6 inhibitors to link HDAC6 inactivation to the restoration of the neuronal structure and synaptic transmission in mouse prefrontal cortex.^49^ However, we and others have shown that the used inhibitors, Tubastatin A, Ricolinostat, and SW-100, are not HDAC6-selective^2,50,51^ but all potently inhibit MBLAC2.^2^ Considering that MBLAC2 shows highest protein expression levels in neuronal tissues,^52,53^ MBLAC2 inactivation must be taken into account as a potential explanation for phenotypes observed after HDAC6 inhibitor treatment.

Further observations support MBLAC2 as a potential driver of other phenotypes. Compounds KV-24, KV-30, KV-46, Tubastatin A, and other phenylhydroxamic acids have been identified to potently inhibit the proliferation of protozoans such as *Toxoplasma gondii* in human host cells.^54–57^ Since MBLAC2 is the only common target of these compounds, its inhibition in host cells may explain the observed effects. This observation raises the interesting and relevant question of whether *T. gondii* depends on host cell membrane processes involving MBLAC2.

Surprisingly, we found three phenylhydroxamic acids, KV-24, KV-30, and KV-50, to bind and inhibit the additional targets HINT1, PDE1C, CK2, and NME1–4. No drug-like small molecule inhibitors have been reported yet for HINT1 and NME1–4 but targeting these proteins may be of interest in certain contexts. HINT1 and NME enzymes play roles in transcriptional regulation, DNA damage repair, and metabolism-guided epigenetics, making them attractive drug targets in cancer treatment. For instance, HINT1 interacts with MITF,^24^ a melanocyte lineage master regulator implicated in melanoma. HINT1 releases MITF upon homo-oligomerization of HINT1 dimers induced by binding of the bivalent HINT1 ligand diadenosine-5′,5-P1,P4-tetraphosphate (Ap4A).^24^ Monovalent ligands, such as KV-30, that compete with Ap4A binding would prevent HINT1 from polymerizing and releasing MITF, and could, therefore, modulate MITF-driven transcriptional programs in melanoma. KV-50, an unspecific inhibitor of NME1, NME2, NME3, NME4, CK2, and MBLAC2, provides a starting point for developing selective NME inhibitors. However, the polypharmacology exhibited by KV-50 may also be of advantage as an anticancer agent because the simultaneous inhibition of the functionally redundant NME1 and NME2 ^31,32^ would lead to enhanced downstream effects. As a further example, a recent study identified NME1 as a potential target in Richter’s Transformation (RT) of Chronic Lymphocytic Leukemia (CLL), where NME1 and the mitochondrial regulator NME4 are upregulated and associated with altered mitochondrial structures and oxidative phosphorylation.^58^ Here, a dual inhibitor of NME1 and NME4 could hold therapeutic potential for treating RT-CLL. Casein kinase 2 (CK2) is another target investigated for cancer therapies.^44^ The CK2 inhibitors Silmitasertib and SGC–CK2–2 are considered chemical probes, but Kinobead profiling of Silmitasertib revealed off-target binding to several kinases.^27,59^ Since KV-50 binds only NMEs and MBLAC2 without targeting other kinases, it could serve as a starting point for designing selective CK2 inhibitors. Conversely, Silmitasertib and SGC–CK2–2 could potentially bind NMEs.

It is important to note that the HINT1 and pan-NME inhibitors identified in this study are not yet selective probes. However, our chemoproteomic platform provides a foundation for SAR exploration of KV-50, KV-24, and KV-30 derivatives to identify more selective and potent inhibitors. In addition, the docking data and cocrystal structures of these compounds reported here can guide rational design efforts.

In conclusion, chemoproteomic screening of a small library of 53 compounds identified selective MBLAC2 probes and uncovered first-in-class pharmacophores for the until now undrugged targets NME1–4 and HINT1. These discoveries may inform the prospective design of selective inhibitors, which hold promise as therapeutic candidates or as chemical probes for basic research.

## Supplementary Material



## Notes

The mass spectrometry proteomics data has been deposited in the MassIVE proteomics database with the data set identifier MSV000096082 (PDF)
