# Supplementary material for: Serendipitous and Systematic Chemoproteomic Discovery of MBLAC2, HINT1, and NME1‑4 Inhibitors from Histone Deacetylase-Targeting Pharmacophores
Source: ACS Chem Biol. 2025 May 8;20(6):1247–57. doi: 10.1021/acschembio.5c00108 (PMC12186257; doi:10.1021/acschembio.5c00108)
Supplement: Supplementary file 1 [file cb5c00108_si_001.pdf]

Supporting Information

Serendipitous and systematic chemoproteomic discovery of MBLAC2, HINT1, and NME1-4 inhibitors from HDAC-targeting pharmacophores

Severin Lechner, Shuyao Sha, Jigar Paras Sethiya, Patrycja Szczupak, Rafal Dolot, Santosh Lomada, Amirhossein Sakhteman, Johanna Tushaus, Polina Prokofeva, Michael Krauss, Ferdinand Breu, Katharina Vögerl, Martin Morgenstern, German Mouse Clinic Consortium, Martin Hrabě de Angelis, Volker Haucke, Thomas Wieland, Carston Wagner, Guillaume Médard, Franz Bracher, Bernhard Kuster**

Table S1| Compound target affinity data from chemoproteomic competition assay. The table shows the pK_D^{app} values determined via chemoproteomic selectivity profiling and related to Figure 1a. Values for compound target pairs that did not induce a dose-dependent competition are described as p pK_D^{app} <4 or p pK_D^{app} < 4.52, i.e., binding at concentrations higher than the highest profiled concentration of 30 µM or 100 µM.

	PTACH	SBHA	Tinostamustine	AES-135	SH507	J22352	SW-100	ACY-1083	QTX125	ACY-957	BRD-6929	BRD-9757	SKLB-23bb	ibuproxam
ISOC1	<4.52	5.66	5.18	<4	<4.52	<4.52	<4.52	<4.52	<4	<4.52	<4.52	<4	5.63	<4
ISOC2	<4.52	5.89	5.52	<4	<4.52	<4.52	<4.52	<4.52	<4	<4.52	<4.52	<4	<4.52	<4
ALDH2	<4.52	5.46	5.56	<4	<4.52	<4.52	<4.52	<4.52	<4	<4.52	<4.52	4.97	<4.52	4.62
GATD3A	<4.52	5.37	4.52	<4	<4.52	<4.52	<4.52	<4.52	<4	<4.52	<4.52	5.05	<4.52	4.52
MBLAC2	<4.52	4.52	5.73	5.39	6.09	6.20	7.12	5.17	5.29	<4.52	<4.52	<4	<4.52	<4
HDAC6	6.36	5.90	6.70	<4	<4.52	5.39	5.92	6.06	6.15	<4.52	<4.52	4.78	<4.52	<4
HDAC10	<4.52	5.48	5.51	<4	<4.52	<4.52	<4.52	<4.52	<4	<4.52	<4.52	4.86	<4.52	<4
HDAC1	6.14	4.52	5.72	<4	<4.52	<4.52	<4.52	<4.52	4.81	6.26	5.83	<4	<4.52	<4
HDAC2	5.98	4.52	5.43	<4	<4.52	<4.52	<4.52	<4.52	4.76	5.93	5.62	<4	<4.52	<4
HDAC3	6.53	5.02	5.93	<4	<4.52	<4.52	<4.52	<4.52	5.01	<4.52	<4.52	<4	<4.52	<4
HDAC8	<4.52	<4	<4	<4	<4.52	<4.52	<4.52	<4.52	<4	<4.52	<4.52	<4	<4.52	<4
HDAC4	<4.52	<4	<4	<4	<4.52	<4.52	<4.52	<4.52	<4	<4.52	<4.52	<4	<4.52	<4
HDAC5	<4.52	<4	<4	<4	<4.52	<4.52	<4.52	<4.52	<4	<4.52	<4.52	<4	<4.52	<4
HDAC7	<4.52	<4	<4	<4	<4.52	<4.52	<4.52	<4.52	<4	<4.52	<4.52	<4	<4.52	<4

Table S2| Overview of compounds synthesized for the focused library to screen for selective MBLAC2 inhibitors. The table indicates whether compound synthesis has been reported before or whether synthesis is reported here for the first time with details in the supporting information.

Compound	Synthesis procedure
KV-24	Reference[56]
KV-30	Reference[56]
KV-43	Supporting information
KV-46	Reference[16]
KV-49	Supporting information
KV-50	Supporting information
KV-65	Reference[16]
KV-70	Reference[16]
KV-79	Reference[16]
KV-83	Supporting information
KV-92	Supporting information
KV-94	Reference[16]
KV-103	Reference[16]
KV-111	Supporting information
KV-129	Reference[16]
KV-136	Reference[16]
KV-157	Reference[16]
KV-162	Reference[16]
KV-172	Reference[16]
KV-176	Reference[16]
KV-181	Reference[16]
KV-184	Reference[16]
MM-7	Supporting information
MM-9	Supporting information
MM-17	Supporting information
MM-20	Supporting information
MM-21	Supporting information

MM-24	Supporting information
MM-25	Supporting information
MM-27	Supporting information

Table S3| Compound target affinity data from chemoproteomic profiling of the focused MBLAC2 targeting library. The table shows the EC_{50} values in µM as determined via chemoproteomic selectivity profiling and related to figure 2. The highest assayed concentration was 30 µM for KV24 and KV30 and 100 µM for all other compounds.

	KV-24	KV-30	KV-43	KV-46	KV-49	KV-50	KV-65	KV-70	KV-79	KV-94	KV-129	KV-172
HDAC6	>30.0	>30.0	7.82	2.76	17.52	>100.0	2.41	1.01	29.92	4.70	0.49	1.75
MBLAC2	0.08	0.03	0.23	0.08	0.43	1.38	0.04	0.26	0.02	0.14	0.05	0.04

Table S4| Crystallographic parameters and data collection statistics.

PDB ID	9GYP	9GYQ
Crystallization conditions	10 % w/v PEG4000, 0.1 M sodium cacodylate pH 6.0	10 % w/v PEG4000, 0.1 M sodium cacodylate pH 6.0
Crystal size (µm)	200 × 120 × 30	150 × 100 × 20
Ligand	KV24	KV30
Ligand code	A1IQU	A1IQV
Soaking time (min.)	15	15
X-ray source	Rigaku XtaLAB Synergy-S	
Wavelength (Å)	1.54184	
Detector	HyPix-6000HE	
Oscillation width (°)	0.38	0.38
Temperature (K)	100	100
No. of frames	640	399
Space group	C2	C2
Unit-cell parameters		
a (Å)	77.96	78.01
a (Å)	46.45	46.47
c (Å)	63.79	63.84
α (°)	90.00	90.00
β (°)	94.53	94.60
γ (°)	90.00	90.00
Total no. of reflections	79203 (2946)	41823 (3160)
Unique reflections	21094 (1233)	15270 (1147)
Completeness (%)	99.3 (98.7)	98.2 (99.5)
Resolution (Å)	18.41-1.80 (1.84-1.80)	18.02-2.00 (2.05-2.00)
Rmergea	0.056 (0.344)	0.065 (0.206)
Rp.i.m	0.032 (0.251)	0.045 (0.141)
Multiplicity	3.8 (2.4)	2.7 (2.8)
Mosaicity	1.16	1.32
Wilson B factor	12.3	12.6
Mean I/sd(I)	15.1 (2.9)	12.3 (4.8)
CC(1/2)	0.998 (0.874)	0.996 (0.944)

Table S5| Refinement statistics.

PDB ID	9GYP	9GYQ
No. of reflections used in refinement	20088	14512
No. of reflections used to Rfree	981	740
Rcryst (Rfree)	0.141 (0.191)	0.141 (0.188)
No. of non-H-atoms		
Protein	1897	1815
Solvent	260	218
Ligand	23 (KV24) + 2×5 (SO42-)	24 (KV30) + 2×5 (SO42-)
R.m.s.d.s from ideal values		
Bond lengths (Å)	0.008	0.008
Bond angles (°)	1.644	1.630
Ramachandran plot		
Favored [%]	99.1	99.1
Allowed [%]	0.9	0.9
Outliers [%]	0	0

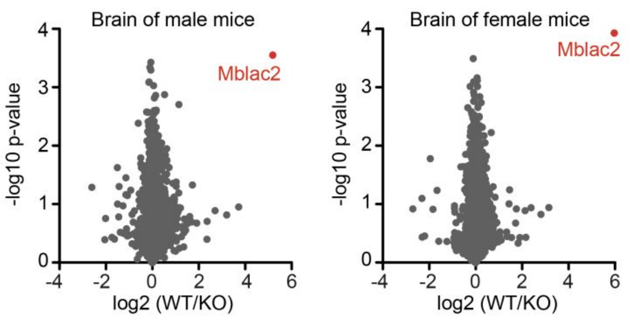

Fig. S1| Comparison of MBLAC2 KO and WT mouse brain proteome. Deep-frozen whole brain samples of three KO and three WT mice of each sex were subjected to DIA proteomics analysis. Volcano plots show the data for all >8000 proteins quantified across all samples. MBLAC2 peptides were only identified in WT samples. Minimum values were imputed for proteins detected only in WT and not in KO to enable WT/KO ratio calculations and to show MBLAC2 in the plot.

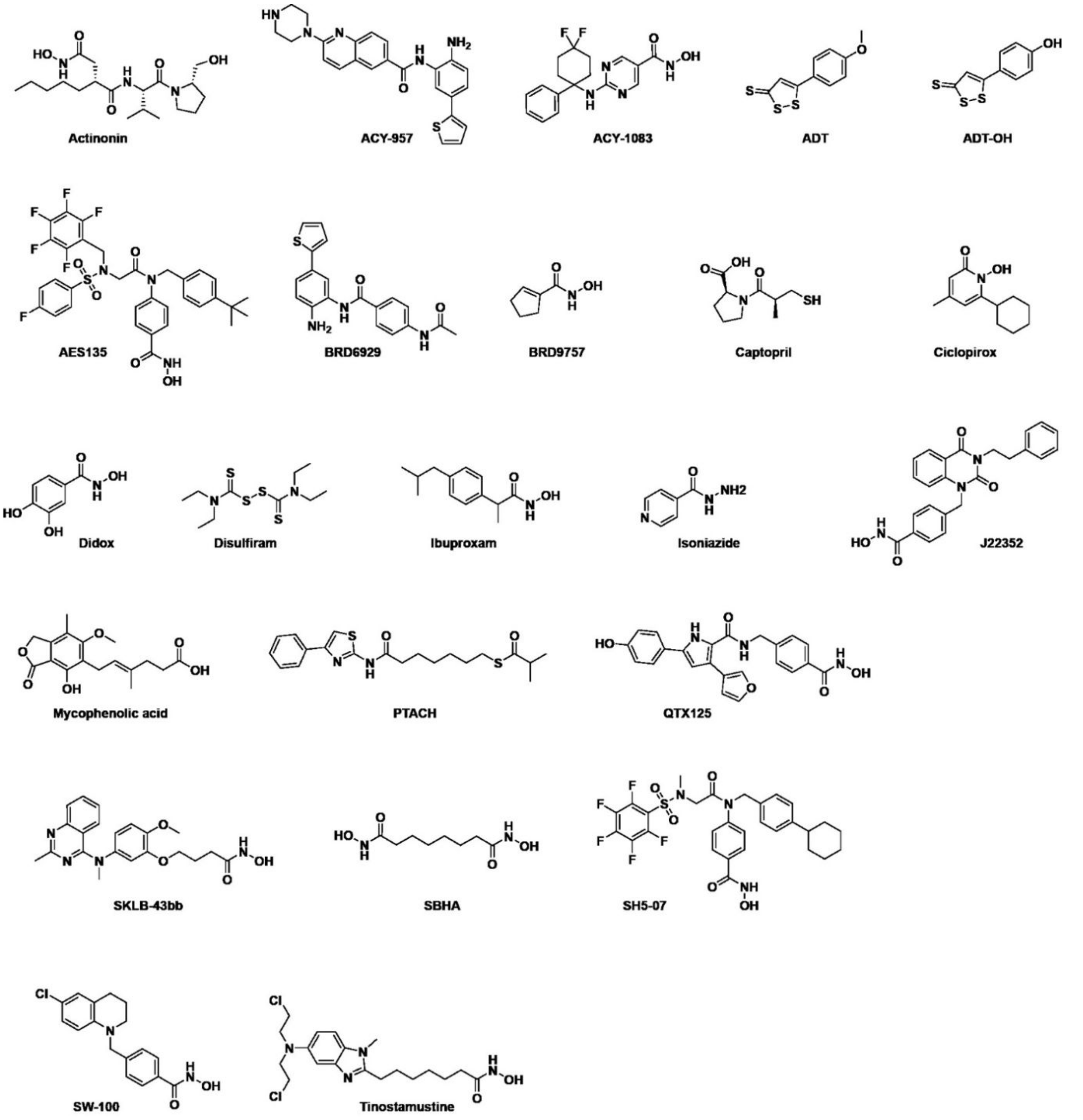

Fig. S2| Structures of HDAC inhibitors and other metal-chelating compounds profiled for HDAC and MBLAC2 binding.

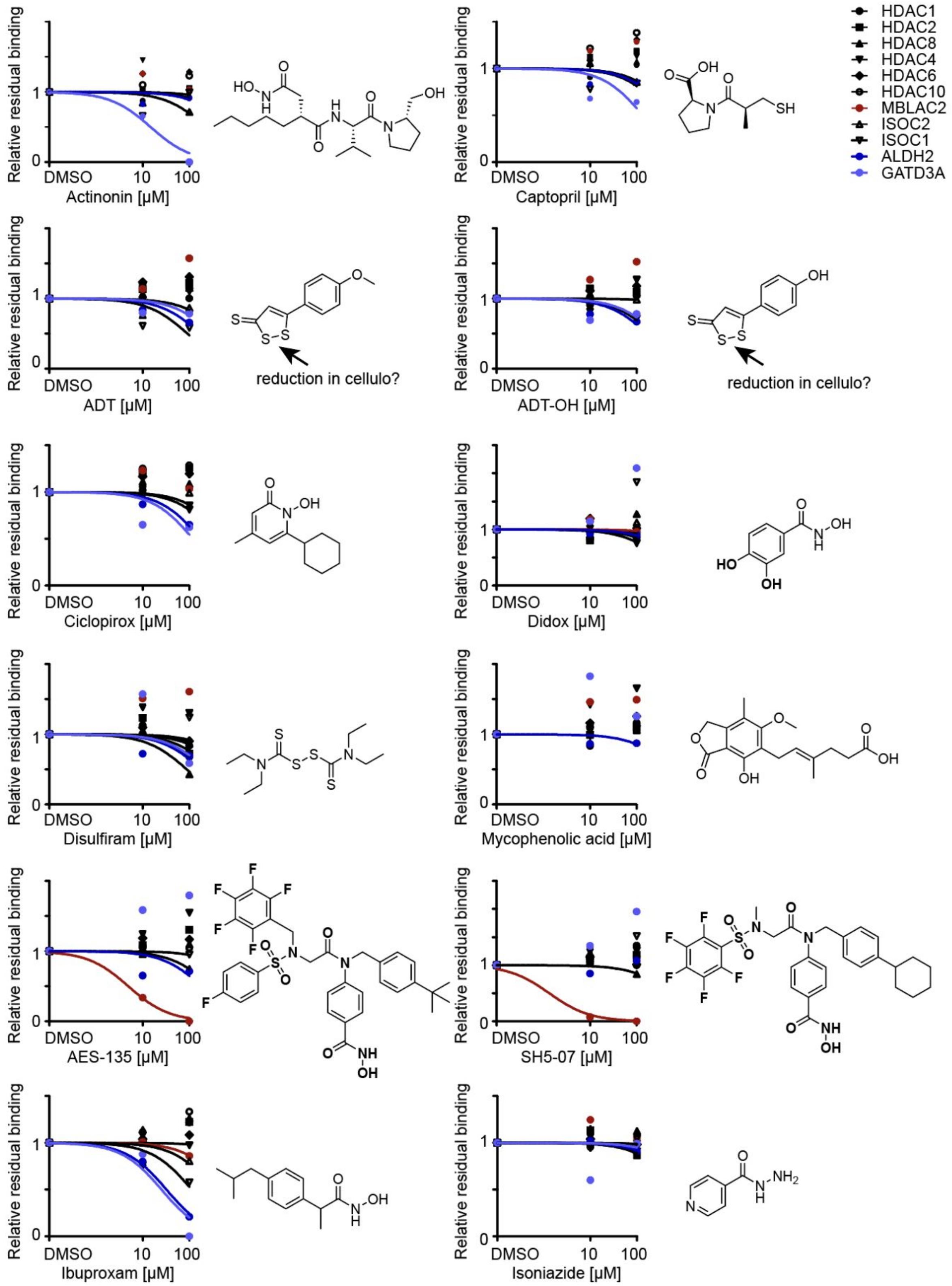

Fig. S3 | 2-dose chemoproteomic screen of metal-chelating compounds for the discovery of MBLAC2 inhibitors.

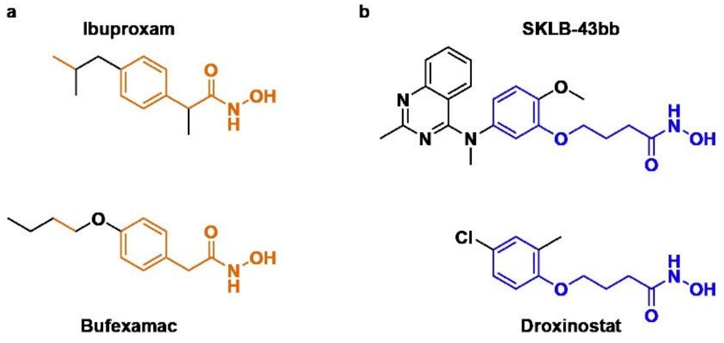

Fig. S4| Comparison of chemical structures of GATD3A, ALDH2, and ISOC1 binders. a, the common features of ALDH2/GATD3A binders Ibuproxam and Bufexamac are highlighted in orange. **b**, the common features of ISOC1 binders SKLB-43bb and Droxinostat are highlighted in blue.

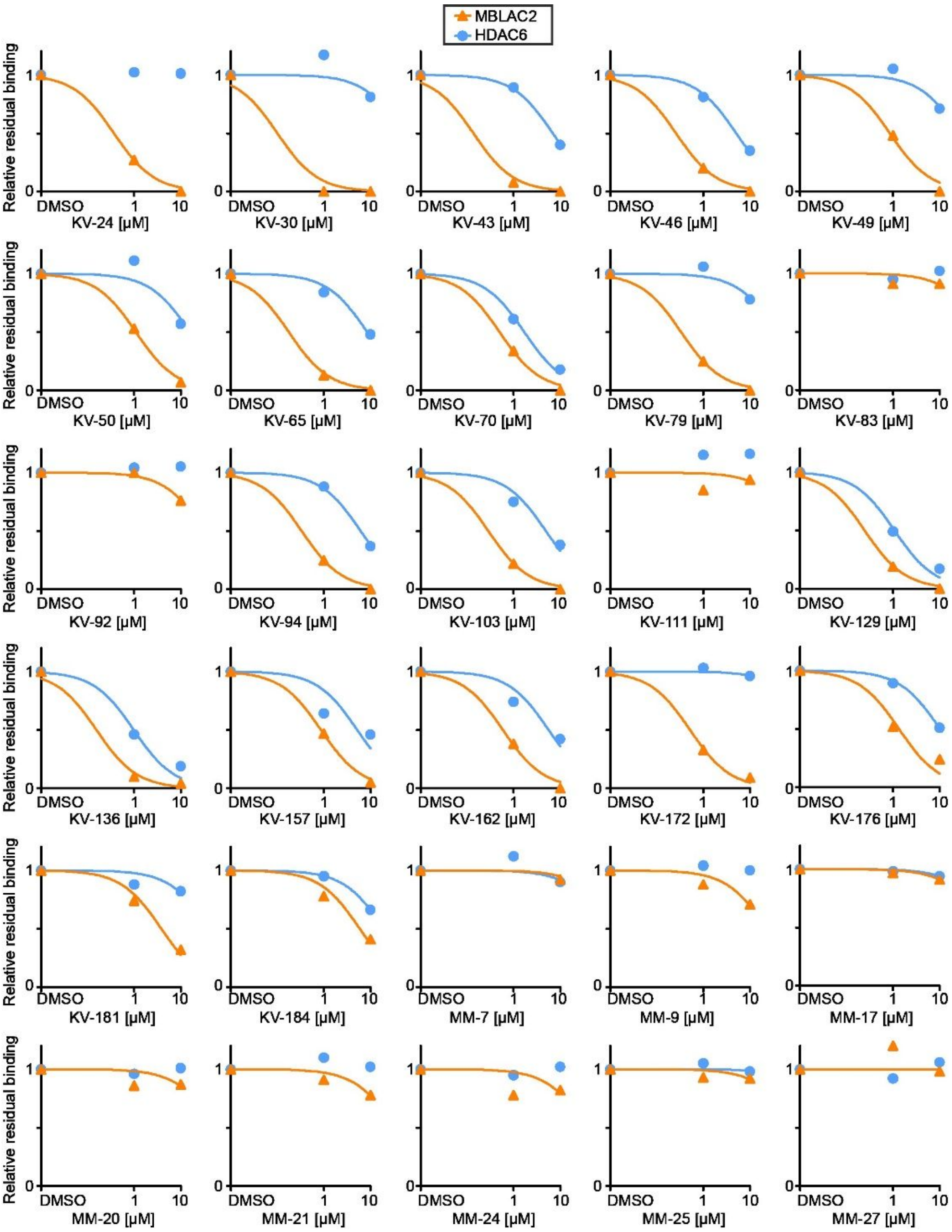

Fig. S5| 2-dose chemoproteomic competition assay as screen for MBLAC2 selective inhibitor candidates.

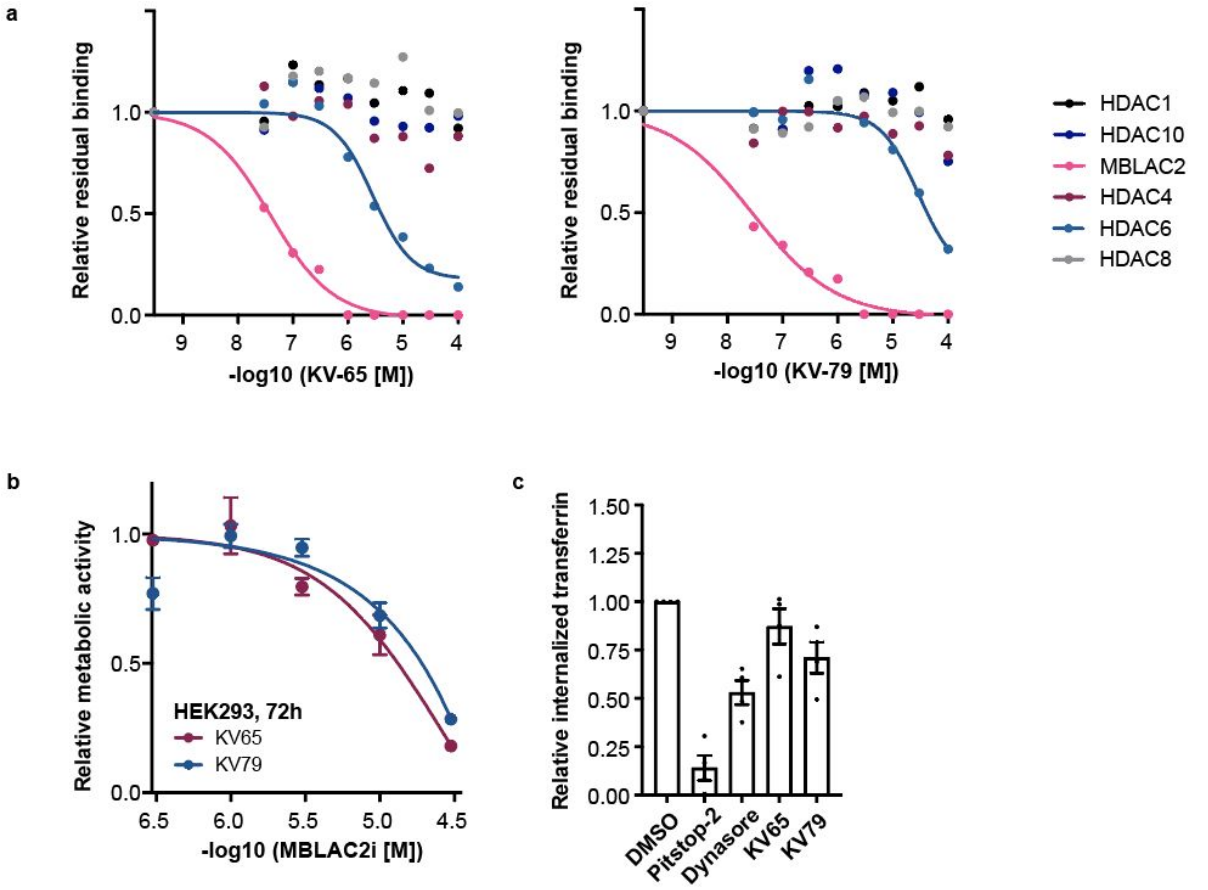

Fig. S6| Characterization of MBLAC2 inhibitors. a, 9-dose competition assay of MBLAC2 selective inhibitors KV-65 and KV-79. **b**, Impact of MBLAC2i on cell metabolic activity assessed in an alamar blue assay. Cells were treated in triplicates with five concentrations of MBLAC2i and a DMSO vehicle control for 72h. **c**, Clathrin-mediated endocytosis assay based on measurement of fluorescent transferrin uptake. Dynasore and Pitstop-2 serve as positive controls. HeLa cells were pre-incubated with 100 µM Dynasore, 2 µM KV65, or 2 µM KV79 for 20 h before the microscopy readout. 30 µM Pitstop-2 was added only for 15 min before readout.

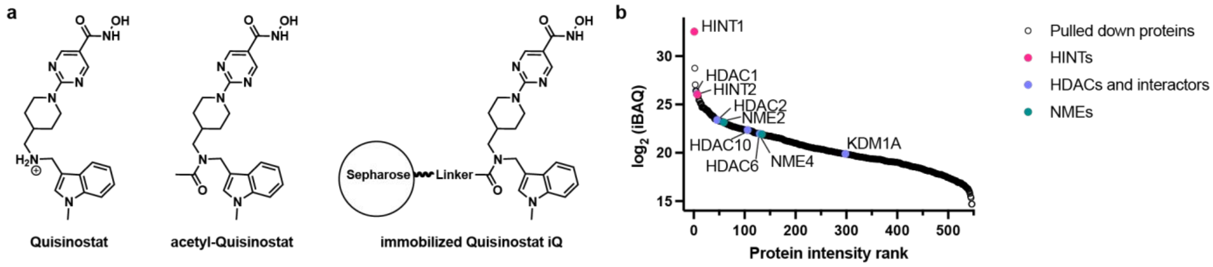

Fig. S7| Ranking plot of proteins pulled down by immobilized Quisinostat, iQ. a, Structures of the unspecific HDAC inhibitor Quisinostat, acetyl-Quisinostat, and the Quisinostat-based affinity matrix iQ used for the pulldown of metalloenzymes. Quisinostat is depicted in its protonated and hence positively charged form, which is predominant at physiological pH. **b,** Previously published pulldown data[2] was reanalyzed for the enrichment of identified off-targets. iBAQ intensities approximate the copy number of pulled-down proteins relative to each other. For instance, for each pulled down Quisinostat target HDAC1 ($\log_2$(iBAQ) = 32.5), around 85 HINT1 proteins ($\log_2$(iBAQ) = 26.1) are pulled down.

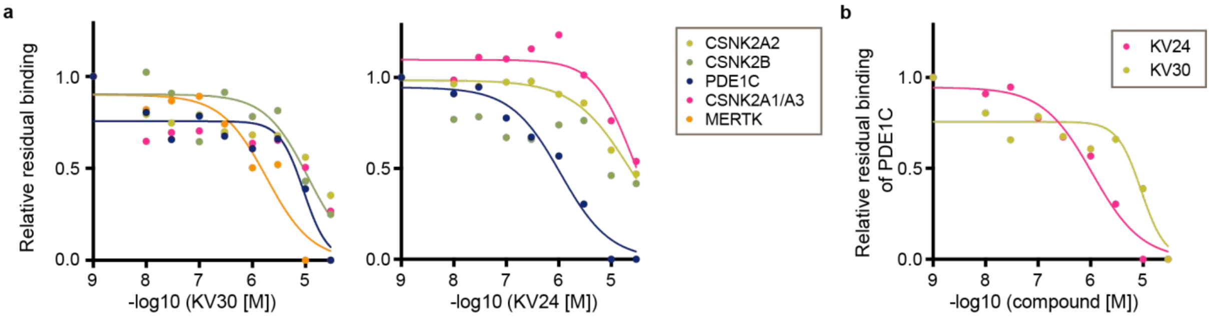

Fig. S8| Kinobeads assay for the identification of potential nucleotide binding off-targets. KV-24 and KV-30 were analyzed for kinase binding in the chemoproteomic kinobead assay setup. Of the more than 190 robustly quantified kinases, only MERTK and casein kinase 2 complex members showed binding at high concentrations. Additionally, the cyclic nucleotide phosphatase PDE1C showed dose-dependent competition.

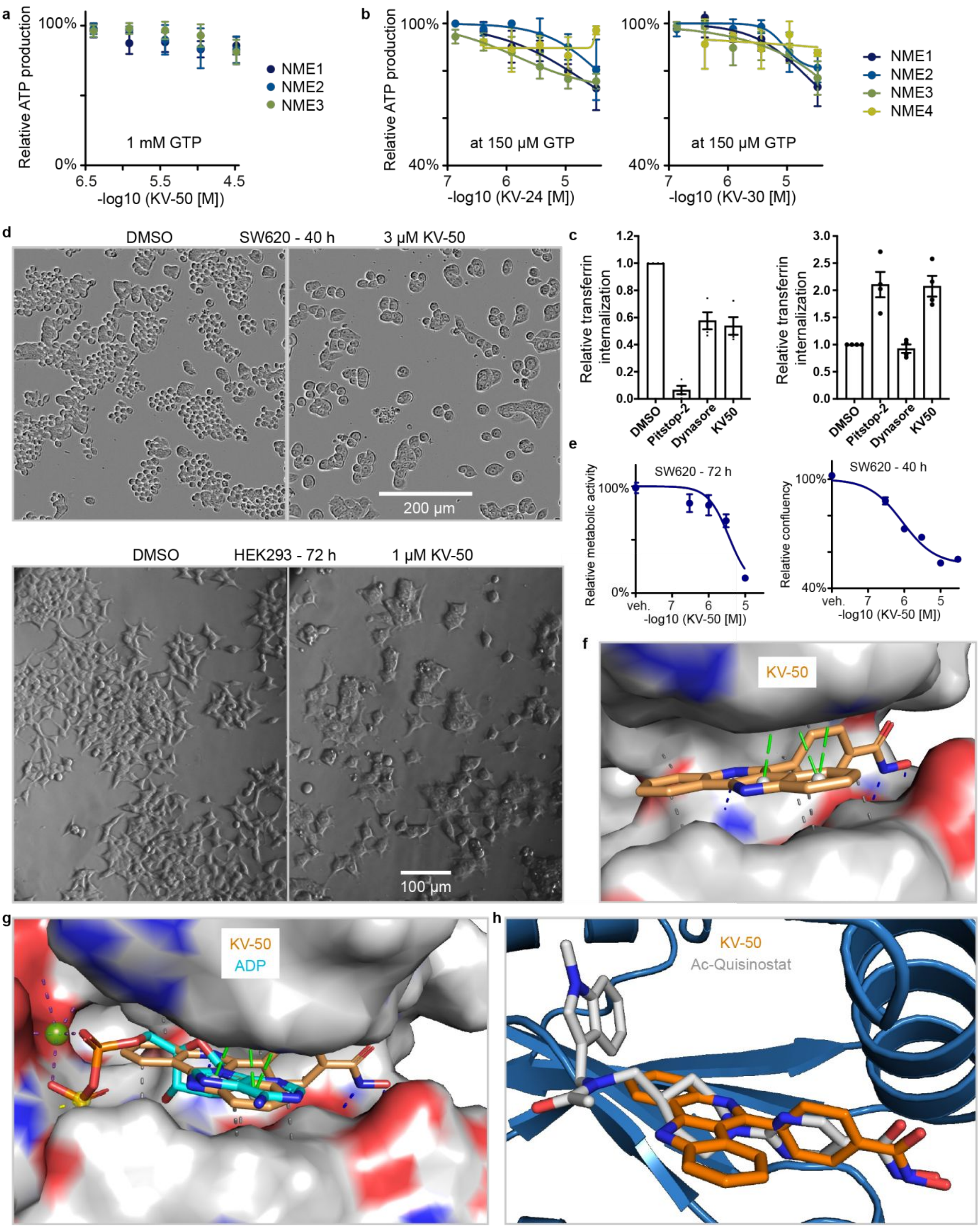

Fig. S9| KV-50 is a competitive NME1-4 inhibitor. a, NME enzyme activity assay at different KV-50 concentrations and in the presence of a large excess of substrate GTP. **b**, NME enzyme activity assay in the presence of potential inhibitors KV-24 and KV-30. **c**, exemplary microscope images of SW620 and HEK293 cells treated with KV-50 or vehicle control DMSO. Concentrations and incubation time are indicated in the figure. **d**, Dose-dependent effect of KV-50 treatment on SW620 confluency and metabolic activity (Alamar blue assay). Incubation times are indicated in the figure panel. **e**, Exemplary docking position of KV-50 in NME1 (PDB: UCN1).

Potentially relevant interactions are indicated by green lines (pi-stacking), blue lines (hydrogen bonds), and grey lines (van-der-Waals interactions). **f**, Docking pose of KV-50, as shown in e, overlaid with the ADP-cofactor (cyan) from the co-crystal structure (PDB: UCN1). **g**, Docking poses of the NME-binding affinity matrix iQ analog acetyl-Quisinostat overlaid with the docking pose of KV-50.

Methods

Chemoproteomic competition assays

Sample preparation. For the MBLAC2 and HDAC inhibitor competition assay, the generation of affinity matrices, lysate generation, pulldown assay, and sample preparation were performed as described in detail in REF[1]. For the kinobeads assay, the generation of affinity matrices, lysate generation, pulldown assay, and sample preparation were performed as described in detail in REF[2].

LC-MS/MS. The different sample types were measured with different LC-MS/MS settings. MBLAC2 2-dose competition assays were measured with the Lumos1 setup, MBLAC2 9-dose competition assays were measured with the Lumos2 setup, and kinobeads assays were measured with the Eclipse setup.

Peptides were analyzed via LC–MS/MS on a Dionex Ultimate3000 nano HPLC coupled to either one of two Orbitrap Fusion Lumos mass spectrometers, or to an Orbitrap Eclipse mass spectrometer, operated via the Thermo Scientific Xcalibur software.

In the all setups, peptides were loaded on a trap column (100 µm × 2 cm, packed in-house with Reprosil-Gold C18 ODS-3 5 µm resin, Dr. Maisch, Ammerbuch) and washed with 5 µL/min solvent A (0.1% formic acid in HPLC grade water) for 10 min. Peptides were then separated on an analytical column (75 µm × 40 cm, packed in-house with Reprosil-Gold C18 3µm resin, Dr. Maisch, Ammerbuch) using a 50 min gradient ranging from 4 to 32% solvent B (0.1% formic acid, 5% DMSO in acetonitrile) in solvent A (0.1% formic acid, 5% DMSO in HPLC grade water) at a flow rate of 300 nL/min.

The mass spectrometers were operated in data-dependent mode, automatically switching between MS1 and MS2 spectra. MS1 spectra were acquired over a mass-to-charge (m/z) range of 360–1300 m/z at a resolution of 60,000 (at m/z 200) in the Orbitrap using a maximum injection time of 50 ms and an automatic gain control (AGC) target value of 4e5.

In Lumos settings, up to 12 peptide precursors were isolated in (isolation width of 1.2 Th for Lumos1 and 1.7 for Lumos2, maximum injection time of 75 ms, AGC value of 1e5 for Lumos1 and 2e5 for Lumos2), fragmented by HCD using 30% normalized collision energy (NCE) and analyzed in the Orbitrap at a resolution of 15,000 (Lumos2) or 30,000 (Lumos1). The dynamic exclusion duration of fragmented precursor ions was set to 20 s (Lumos1) or 30 s (Lumos2). In Eclipse measurements, a cycle time of 2 s was chosen. Peptides were isolated in the quadrupole with an isolation offset of 1.2 (isolation width of 1.2 Th, maximum injection time of 54 ms, AGC target of 200%) and fragmented by HCD with an NCE of 30% and analyzed in the Orbitrap at a resolution of 30,000. The dynamic exclusion on the Eclipse was set to 20 s.

*Data analysis***.** The data analysis of MBLAC2/HDAC inhibitor competition assays was performed as described in detail in REF[1]. LFQ intensity values were used for the calculation of relative residual binding to the affinity matrix.

The data analysis of kinobeads inhibitor competition assays was performed as described in detail in REF[2]. Raw intensity values were used for the calculation of relative residual binding to the affinity matrix.

Mblac2 mouse model generation and phenotyping at the German Mouse Clinic. From the age of 8-16 weeks, the Mblac2 -/- mice were phenotyped systematically in the German Mouse Clinic (GMC) at Helmholtz Munich (www.mouseclinic.de) as described previously[3–5] and in accordance with the standardised phenotyping pipeline of the IMPC (IMPReSS: https://www.mousephenotype.org/impress/index). 8 female and 10 male homozygous mutant animals (Mblac2 KO) and 11 female and 13 male wildtype control animals (wt) were compared. Phenotyping protocols are in agreement with the IMPC standard procedures (https://www.mousephenotype.org/impress/PipelineInfo?id=14) and published elsewhere[4,6–11]. The information about the Mblac2em1(IMPC)HmguMblac2 (MBLAC2 KO) mouse line generation as well as the possibility to order the mouse model can be found at https://www.mousephenotype.org/data/genes/MGI:1920102#order.
Mice were housed in IVC cages with water and standard mouse chow available ad libitum according to the European Union directive 2010/63/EU and GMC housing conditions (www.mouseclinic.de). All tests performed in this study met approval by, and complied with, the rules of the district government of Upper Bavaria (Regierung von Oberbayern) Germany.
For proteomics analysis, perfused brains were collected from MBLAC2 KO (n=3 males and 3 females), and WT (n=3 males and 3 females) adult mice.

Mouse brain proteomics – sample preparation. Fresh frozen mouse brains were pulverized using using a tissue lyser (Qiagen, 3 min at 300 s1) by adding a metal ball (diameter 5 mm) at temperatures around -80 °C. Protein samples were prepared via an SDS-lysis and SP3 bead sample preparation protocol. In brief, pulverized mouse brain was transferred to Eppendorf reaction vessels and lysed with a SDS lysis buffer (2% SDS in 10 mM Tris HCl, pH = 8). Then, lysates were heated to 95 °C for 10 min under shaking and 10% trifluoroacetic acid (TFA) was added to a give a solution with a final concentration of 1% TFA. After 1 min incubation and vortexing, a solution of 20% N-methyl morpholine (NMM) was added to give solution with final concentration of 2% (v/v) NMM. The samples were centrifuged at 21000 g for 5 min, and supernatant was used to measure the protein concentration by means of the BCA assay (Thermo, Pierce).

Subsequently, proteins were cleaned via the SP3 protocol[12] with minor adaptions on a Bravo Automated Liquid Handling Platform (Agilent Technologies). 180 μg of protein was mixed with 1 mg of beads (1:1 mix, Sera-Mag beads A and B) for 10 min at 1200 rpm and room temperature. Sample volumes were adjusted to 120 μL with 100 mM HEPES (pH 8.5). Protein precipitation was induced by adding 70% ethanol. The beads were washed thrice with 80% ethanol and once with 100% ACN. Thereafter, proteins were reduced and alkylated in a digestion buffer (10 mM TCEP, 50 mM CAA, 100 mM HEPES, pH 8.5) at 37 °C and 1200 rpm for 1 h. Proteins were then digested by incubating with trypsin (enzyme-to-substrate ratio of 1:50) at 37 °C and 1200 rpm for 16 h. The resulting digests were acidified by TFA to a final concentration of 1%.

Subsequently, samples were desalted using the stage tip protocol[13]. Five disks of C18 material (3M Empore) were packed into a 200 μL pipette tip. The StageTips were pre-equilibrated with a sequential wash using ACN, 0.1% FA in 40% ACN, and 0.1% FA. After loading the peptides and reapplying the flowthrough, the StageTips were washed with 0.1% FA, and the peptides were eluted using 0.1% TFA in 40% ACN. Cleaned peptide solutions were dried using a speedvac and stored at -80 °C until measurement.

Mouse brain proteomics – LC-MS/MS. Proteome analysis of brain mouse tissue samples was performed on a micro-flow LC timsTOF HT setup described in detail in REF[14]. Liquid chromatography in micro-flow mode was performed on a Vanquish Neo UHPLC System (Thermo Fisher Scientific) at a flow rate of 50 µL/min. Peptide separation was performed on an Acclaim™ PepMap™ C18 column (15 cm lengths, 1 mm inner diameter, 2 um particle size, Thermo Fisher Scientific, #164711) using a binary gradient of buffer A (0.1% FA with 3% DMSO in dH_2O) and buffer B (0.1% FA with 3% DMSO in 80% Acetonitril (ACN)). 10 µg of peptide mix was injected and analyzed using a 58 min gradient starting at 1% B up to 45% B (1 h method). Data were acquired in data independent acquisition (DIA) mode utilizing a 3x8 dia-PASEF window scheme covering a mobility range between 0.64 to 1.45 V^*s/cm^2. A ramp time of 100 ms and advanced collision energy settings were applied.

Mouse proteomics data analysis. The data files were analyzed with DIA-NN 1.8.1. The DIA library was created with the internal deep learning-based tool after in silico Trypsin/P digestion of the uploaded protein sequence FASTA file. The FASTA file contained 17,807 entries of canonical protein sequences and another FASTA file with 381 entries of potential contaminants was added (https://pubs.acs.org/doi/abs/10.1021/acs.jproteome.2c00145). All parameters were kept at default settings and C-carbamidomethylation, oxidation of methionine, N-terminal methionine excision as well as N-terminal acetylation were allowed as posttranslational modifications. The resulting protein group tables were further analyzed by Perseus 1.6.2.2. Proteins were log2 transformed, filtered for contaminants and for proteins that were present in at all replicates of at least one triplicate. Missing values were then replaced by randomly choosing values from a normal distribution with a downshift of 1.8 and a width of 0.3.

CME of transferrin and confocal microscopy analyses. For transferrin uptake experiments HeLa cells were seeded on Matrigel-coated coverslips, and incubated with 0.1 % DMSO, or with 100 µM dynasore, 20 µM KV50, 2 µM KV65, or 2 µM KV79 in media containing 10% FCS at 37°C for 20 h. Cells were about 60-70% confluent the next day. Then, cells were incubated with the same inhibitors, or with 30 µM Pitstop-2, in serum-free medium for 15 min. All inhibitor treatments were performed in duplicate, to enable quantification of surface levels of transferrin receptors, and to assess the capability of transferrin receptor endocytosis.

To measure transferrin receptor endocytosis, cells in inhibitor-containing media were moved on ice, supplemented with 5 µg/mL AlexaFluor568-labeled transferrin, and subsequently incubated for 15 min at 37°C. Cells were then washed once for 2 min in ice-cold acid buffer (0.1 M acetic acid pH5.3, 500 mM NaCl), and twice in ice-cold PBS containing 10 mM $MgCl_2$, and finally fixed in 4% PFA / 4% sucrose for 30 min at RT. To determine surface levels of transferrin receptors cells were incubated on ice for 45 min with 15 µg/mL AlexaFluor568-transferrin, washed twice briefly with ice-cold PBS, and fixed in 4% PFA / 4% sucrose on ice for 5 min, and thereafter for 30 min at RT. All coverslips were mounted in Shandon™ Immu-Mount™ containing Hoechst33342, and imaged on a spinning disk confocal microscope (Ultraview ERS, Perkin Elmer) equipped with a 40x oil-immersion objective. Quantitative analysis was performed using ImageJ. Fluorescence intensities gained from individual images were corrected for background, and divided by the number of cells. Values were normalized to controls (DMSO-treated cells). At least 75 cells were analysed for each condition in each experiment.

Biochemical hHint1 assay. All the experiments were conducted under kinetics mode using Varian Cary Eclipse Fluorimeter. First, we reproduced the enzyme kinetics using the known substrate tryptamine 5'-adenosine phosphoramidate, TrpA (K_m = 0.1388). To determine the half inhibitory concentration (IC_{50}) of test compounds (KV-24 and KV-30), we kept the hHint1 enzyme (0.002 µM) and substrate concentration (0.5 µM) constant. Briefly, hHint1 was pre-incubated with six different concentration of

test compounds (0.1, 1, 5, 10, 25, and 50 µM) in 600 µL of assay buffer (20 mM HEPES, 1mM $MgCl_2$ pH- 7.2) for 30 s at 25ºC. Next the substrate TrpA was added to the cuvette and the rate of hydrolysis was monitored at excitation wavelength 280 nm and emission wavelength 360 nm. For a negative control, a DMSO blank was treated in the same way. The fluorescence intensity was measured for 1 minute, and a slope was calculated (Intensity/time).

Nucleotide diphosphate kinase activity assays. The phosphotransfer activity of the NDPKs (NME1-4) was measured in a reaction mixture containing buffer A (20 mM Tris-HCl pH 8.0, 150 mM NaCl, and 1 mM MgCl2). All the samples were pipetted in replicates on a 384 well plate. One volume of the respective NDPK protein solution (NME1-4) was added from stock proteins (diluted in buffer a to a concentration of 300 pM) to the wells in four replicates. To these wells, one volume of the inhibitors (Six dilutions were prepared, starting with a 30 µM stock solution and then performing three-fold serial dilutions) was added and incubated at room temperature for 30 minutes. Then, one volume of the substrate mixture (300 µM GTP and 30 µM ADP) was added to the protein-inhibitor mixture-containing wells and incubated at room temperature for 30 minutes. Finally, three volumes of the Kinase-GLO reagent (Promega V6711) containing an ATP-dependent firefly luciferase were added to all the wells and five minutes later, the luminescence was measured using a plate reader. Eight different concentrations of ATP (0 - 10 µM) were used as standards for a calibration curve to calculate the amount of ATP produced by the different NDPKs.

Docking of ligands to NME1. The x-ray crystal structure of nucleoside diphosphate kinase A, NME1 (1UCN), in complex with ADP was retrieved from protein data bank as PDB format. In order to validate the docking protocol, the structure of co-crystal ligand was re-docked in the binding cavity of the enzyme using a grid box centered at x = -8.676, y = 97.894 and z = 17.288 (which corresponds to the phosphate moiety in the ADP structure). Autodock vina with the exhaustiveness parameter set to 100 was used during docking simulation[15]. Partial atomic charges in the protein structure were calculated using Autodock tools 1.5.7[16]. The obtained RMSD (1.850) between the first top docking pose and the observed coordinates of ADP at crystal structure was used as a measure for the validity of docking method to predict the binding of unknown ligands. To ensure that the obtained RMSD value was not obtained by chance, two other replicates of the same self-docking experiment were repeated using the chain B and Chain C protein complex structures of the same PDB code. Based on the obtained converged docking RMSD values (1.8512, 1.862 and 1.840 with calculated binding energies of -8.1, -8.0 and -8.0 kcal/mol, respectively), the current docking protocol was consequently used for the compounds of interest. The 3D mol2 structures of the compounds were also converted to PDBQT after adding Gasteiger charges using Autodock tools 1.5.7[16].

The first top 5 poses of docking results were extracted for each ligand and used for further analysis and visualizations. PLIP (*fully automated protein–ligand interaction profiler)* was used for 3D visualization of the ligand-protein complexes[17].

hHINT1-compound co-crystal structure determination. hHINT1 crystals were grown by vapor diffusion using hanging drops, where the drops consisted of 2 µL protein and 2 µL well solution. The well solutions contained 10-14% (w/v) PEG4000 and 100 mM sodium cacodylate pH 6.0-6.6. Crystals formed after 4-5 days of incubation at 8 °C. Co-crystals with KV-24 and KV-30 were prepared by soaking pre-formed crystals in mother liquor containing 12.5 mM of the respective ligand for 10-30 minutes. DMSO was used to adequately dissolve the KV compounds, but this was at the expense of the structural integrity of the soaked crystals and thus the ability to soak the crystals for an extended period of time. The soaked crystals did not need to be cryopreserved but had to be mounted on a very thin film of the crystallization buffer. The excess liquid was removed by gently touching the mounting loop against the plate surface and the crystal was then cooled directly in a stream of N_2. Diffraction

data were acquired using a Rigaku XtaLAB Synergy-S diffractometer equipped with a HyPix-6000HE Hybrid Photon Counting (HPC) detector and a sealed microfocus Cu X-ray source, and a low temperature Oxford Cryostream 800 liquid nitrogen cooling system at 100K. The data acquisition strategy was calculated in *CrysAlis PRO* to ensure the desired data redundancy and percent completeness. The data was processed, integrated and scaled using *CrysAlis PRO* and *AIMLESS*[18]. The statistics for data acquisition and processing are listed in Table S4.

Molecular replacement was performed with the hHINT1 coordinates (PDB ID: 6yqm[19]) using *MOLREP* software[20]. Modeling and molecular visualization was performed in *COOT*[21]. Ligand restraints were calculated using *JLigand*[22] and refinement was performed using *REFMAC5*[23]. All refinement steps were monitored with R_{cryst} and R_{free} values. The stereochemical quality of the resulting models was assessed using the program *MOLPROBITY*[24] and the validation tools implemented in *COOT*. The values of the mean temperature factors for protein main and side chains, ligands and water molecules were calculated using the program *BAVERAGE* from the *CCP4* suite[25]. The superposition of protein structures was performed with the program *LSQKAB*[26]. The refinement statistics of the described structures are listed in Table S5.

Chemistry

General information

All chemicals and reagents were purchased from commercial suppliers and used without further purification. Solvents with lower quality than p.a. were purified by distillation. Dry tetrahydrofuran as well as 1,4-dioxane were heated to reflux and distilled over sodium-benzophenone-ketyl.

TLC was performed on 0.2 mm silica gel plates (40 x 80 mm) Polygram® SIL G/UV254 (Macherey-Nagel) with fluorescence indicator using UV-light at 254 nm and 336 nm as well as appropriate staining for detection.

For flash column chromatography (FCC) silica gel 60 with particle size 0.040 - 0.063 mm was used. The solvent systems are described in the individual synthesis protocols.

NMR spectra were recorded with an Avance III HD 400 MHz Bruker BioSpin (400 MHz) or an Avance III HD 500 MHz Bruker BioSpin (500 MHz) with CryoProbe™ Prodigy (both Bruker) and with a Jeol JNMR-GSX 400 (400 MHz) or Jeol JNMR-GSX 500 (500 MHz) (both Jeol). Chemical shifts were referenced to the residual solvent signals and the coupling constants J are shown in Hertz [Hz]. Spectra were processed with the software MestReNova (Mestrelab Research).

High resolution mass spectrometry using electrospray ionization was performed on a Thermo Finnigan LTQ FT (Thermo Electron Corporation). Electrospray ionization was performed using IonMax ion source with a voltage of 4 kV, a heater temperature of 250 °C, the N_2 sheath flow 20 or 25 and sweep flow 5 units.

IR spectra were recorded with a Perkin Elmer FT-IR Paragon 1000 (Perkin Elmer) or recorded on a Jasco FT/IR-4100 using ATR PRO450-S (Jasco Germany) and all spectra were corrected accordingly.

Melting points were measured with a Büchi B-540 (Büchi) apparatus, are presented in degrees Celsius [°C] and are not corrected.

Syntheses under microwave irradiation were performed using Discover (S-Class Plus) SP (CEM).

The purity of all compounds was determined by HPLC performed on a HP Agilent 1100 HPLC (Agilent Technologies) with a G1315A diode array detector, a G1316A ColComp oven, a G1311A QuatPump pump system and an Agilent Poroshell column 120 Å, EC-C18 2.7 µm, 3.0 x 100 mm or a Synergi™ column, 4 µm, Hydri-RP 80 Å, 4.6 x 250 mm, Ea (Phenomenex). For detection, wavelengths of 210 nm and 254 nm were used. The purity of the compounds was shown to be 95 % or higher, if not stated otherwise.

ChemBioDraw Professional 16.0 (CambridgeSoft), MestReNova (Mestrelab Research) and Office 2013 (Microsoft) were used as software.

General procedures

General procedure 1: Synthesis of THP-protected hydroxamic acids by COMU-mediated amidation

The respective carboxylic acid was dissolved under N_2 atmosphere in dry DMF and cooled to 0 °C. Then, trimethylamine or *N*,*N*-diisopropylethylamine were added dropwise. A solution of (1-cyano-2-ethoxy-2-oxoethylidenaminooxy)dimethylamino-morpholino-carbenium hexafluorophosphate (COMU) (1.5 eq.) in dry DMF was added and the mixture stirred for 5 min. Afterwards, a solution of *O*-(tetrahydro-2*H*-pyran-2-yl)hydroxylamine (1.5 eq.) in dry DMF was added. The reaction mixture was stirred at 0 °C for 1 h and then stirred at room temperature. The reaction times are described in the single protocols. The mixture was poured into water and extracted multiple times with DCM. The combined organic phases were washed with brine and dried over magnesium sulfate. After evaporation of solvent, the crude product was purified by FCC.

General procedure 2: Deprotection of *O*-THP-hydroxamates

To a solution of THP-protected hydroxamic acid in DCM 4 M HCl in 1,4-dioxane was added. The exact amounts of HCl are outlined in the single protocols. The reaction mixture was stirred at room temperature. The precipitated hydroxamic acid was collected by filtration and washed with a small amount of cold DCM.

General procedure 3: Hydrolysis of esters

After suspending the ester compound in a mixture of 1,4-dioxane/water (1:1), 2 M NaOH (2.5 eq. or 5.0 eq.) was added, and the mixture was stirred at room or higher temperature. Conversion was monitored by TLC. After complete conversion, the mixture was cooled to room temperature, if necessary, and then acidified with 1 M citric acid or 2 M HCl to pH 4. The precipitated solid was collected by filtration under vacuum, washed with water and dried. If no precipitation has occurred, the mixture was extracted with the described organic solvent. The combined organic phases were washed with brine, dried over magnesium sulfate and the solvent was evaporated.

(±)-4-[(10*H*-Phenoxazin-10-yl)methyl]-*N*-[(tetrahydro-2*H*-pyran-2-yl)oxy]benzamide (KV-40)

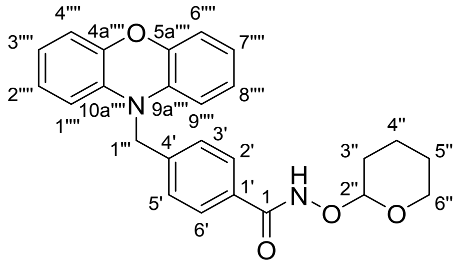

$C_{25}H_{24}N_2O_4$

M_r = 416.48

Synthesis was performed according to General procedure 1. Therefore, 251 mg (0.791 mmol) 4-[(10*H*-phenoxazin-10-yl)methyl]benzoic acid[27], dissolved in 2.0 mL dry DMF, 220 µL (1.59 mmol) triethylamine, 507 mg (1.18 mmol) COMU in 1.0 mL dry DMF and 138 mg (1.18 mmol) *O*-(tetrahydro-2*H*-pyran-2-yl)hydroxylamine in 1.0 mL dry DMF were used. After 3 h, the mixture was extracted three times with DCM (20 mL each). The crude product was purified by FCC (DCM/MeOH = 98:2, R_f = 0.3 and iso-hexane/ethyl acetate = 1:1, R_f = 0.4) and resulted in 174 mg (yield 53 %) of an off-white solid as product.

M.p.: 171 – 172 °C

^{1}H NMR (400 MHz, CD_2Cl_2): δ (ppm) = 8.92 (s, 1H, NH), 7.75 – 7.62 (m, 2H, 2'-H, 6'-H), 7.46 – 7.31 (m, 2H, 3'-H, 5'-H), 6.76 – 6.61 (m, 6H, 2''''-H, 3''''-H, 4''''-H, 6''''-H, 7''''-H, 8''''-H), 6.30 (dd, *J* = 6.4, 1.9 Hz, 2H, 1''''-H, 9''''-H), 5.02 (t, *J* = 3.0 Hz, 1H, 2''-H), 4.82 (s, 2H, 1'''-H), 3.97 (ddd, *J* = 11.9, 8.9, 3.0 Hz, 1H, 6''-H_a), 3.60 (dtd, *J* = 11.1, 4.1, 1.7 Hz, 1H, 6''-H_e), 1.89 – 1.75 (m, 3H, 3''-H_a, 3''-H_e, 4''-H_a/H_e), 1.68 – 1.52 (m, 3H, 4''-H_a/H_e, 5''-H_a, 5''-H_e)

^{13}C NMR (101 MHz, CD_2Cl_2): δ (ppm) = 166.0 (C-1), 145.7 (C-4a'''', C-5a''''), 141.6 (C-4'), 134.1 (C-9a'''', C-10a''''), 131.9 (C-1'), 128.3 (C-2', C-6'), 127.0 (C-3', C-5'), 124.3 (C-2''''/C-4'''', C-8''''/C-6''''), 122.0 (C-3'''', C-7''''), 115.8 (C-4''''/C-2'''', C-6''''/C-8''''), 112.7 (C-1'''', C-9''''), 103.1 (C-2''), 63.2 (C-6''), 49.4 (C-1'''), 28.7 (C-3''), 25.6 (C-5''), 19.3 (C-4'')

IR (KBr): ṽ (cm^{-1}) = 3198, 3064, 2947, 1651, 1613, 1591, 1571, 1490, 1380, 1294, 1272, 1203, 1130, 1111, 1014, 949, 904, 873, 846, 738

HRMS (ESI): m/z = 417.1809 $[M+H]^+$ calculated for $C_{25}H_{25}N_2O_4^+$ 417.1809

4-[(10*H*-Phenoxazin-10-yl)methyl]-*N*-hydroxybenzamide (KV-43)

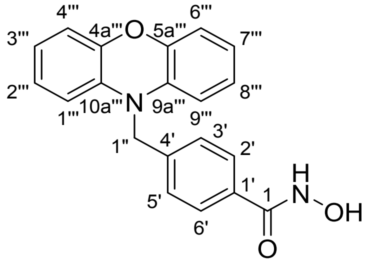

Literature known substance[28]

$C_{20}H_{16}N_2O_3$

M_r = 332.36

Synthesis was performed according to General procedure 2. Therefore, 108 mg (0.259 mmol) (±)-4-[(10*H*-phenoxazin-10-yl)methyl]-*N*-[(tetrahydro-2*H*-pyran-2-yl)oxy]benzamide (**KV-40**) in 6.0 mL DCM and 340 µL (1.36 mmol) 4 M HCl in 1,4-dioxane were used. The reaction mixture was stirred for 14 h and resulted in 20 mg (yield 23 %) of an off-white solid as product.

M.p.: 203 °C

^{1}H NMR (500 MHz, DMSO-*d*$_6$): δ (ppm) = 11.15 (s, 1H, NHO*H*), 9.00 (bs, 1H, N*H*OH), 7.74 – 7.69 (m, 2H, 2'-H, 6'-H), 7.37 – 7.33 (m, 2H, 3'-H, 5'-H), 6.74 (ddd, *J* = 7.9, 7.3, 1.8 Hz, 2H, 2'''-H, 8'''-H), 6.71 (dd, *J* = 7.9, 1.8 Hz, 2H, 4'''-H, 6'''-H), 6.67 (ddd, *J* = 7.9, 7.2, 1.3 Hz, 2H, 3'''-H, 7'''-H), 6.47 (dd, *J* = 8.0, 1.3 Hz, 2H, 1'''-H, 9'''-H), 4.92 (s, 2H, 1''-H)

^{13}C NMR (126 MHz, DMSO-*d*$_6$): δ (ppm) = 164.1 (C-1), 144.1 (C-4a''', C-5a'''), 139.9 (C-4'), 133.1 (C-9a''', C-10a'''), 131.7 (C-1'), 127.3 (C-2', C-6'), 126.2 (C-3', C-5'), 124.1 (C-2''', C-8'''), 121.3 (C-3''', C-7'''), 115.1 (C-4''', C-6'''), 112.4 (C-1''', C-9'''), 46.8 (C-1'')

IR (KBr): ṽ (cm^{-1}) = 3239, 3061, 3017, 2856, 1614, 1569, 1539, 1491, 1429, 1382, 1309, 1273, 1155, 1132, 1015, 893, 844, 739, 637, 582

HRMS (ESI): m/z = 331.1087 [M-H]$^-$ calculated for $C_{20}H_{15}N_2O_3^-$ 331.1088

Methyl 4-{[9-oxoacridin-10(9*H*)-yl]methyl}benzoate (KV-42)

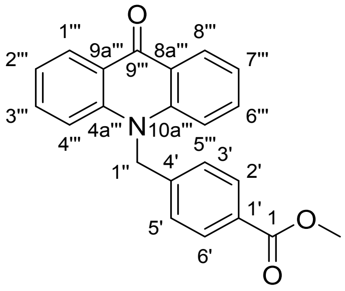

Literature known substance[29]

$C_{22}H_{17}NO_3$

M_r = 343.38

After dissolving 400 mg (2.05 mmol) acridin-9(10*H*)-one under N_2 atmosphere in 2.0 mL dry DMF, 4.08 mL (4.08 mmol) 1 M lithium bis(trimethylsilyl)amide in toluene were added dropwise and the mixture was stirred for 15 min. Then, 798 mg (3.48 mmol) methyl-4-(bromomethyl)benzoate in 1.5 mL dry DMF were added. After a reaction time of 24 h at room temperature, the mixture was poured into water and extracted four times with DCM (30 mL each). The combined organic phases were washed with brine and dried over magnesium sulfate. Then, the solvent was evaporated, and the crude product was purified by FCC (DCM/MeOH = 99:1, R_f = 0.4) resulting in 542 mg (yield 77 %) as light-yellow crystals.

M.p.: 235 – 236 °C

^{1}H NMR (500 MHz, CD_2Cl_2): δ (ppm) = 8.52 (dd, *J* = 8.0, 1.7 Hz, 2H, 1'''-H, 8'''-H), 8.02 – 7.97 (m, 2H, 2'-H, 6'-H), 7.65 (ddd, *J* = 8.7, 6.9, 1.8 Hz, 2H, 3'''-H, 6'''-H), 7.36 – 7.28 (m, 6H, 3'-H, 5'-H, 2'''-H, 4'''-H, 5'''-H, 7'''-H), 5.66 (s, 2H, 1''-H), 3.87 (s, 3H, CH_3)

^{13}C NMR (126 MHz, CD_2Cl_2): δ (ppm) = 178.3 (C-9'''), 166.9 (C-1), 143.0 (C-4a''', C-10a'''), 141.6 (C-4'), 134.6 (C-3''', C-6'''), 130.8 (C-2', C-6'), 130.5 (C-1'), 128.1 (C-1''', C-8'''), 126.4 (C-3', C-5'), 123.2 (C-8a''', C-9a'''), 122.2 (C-2''', C-7'''), 115.7 (C-4''', C-5'''), 52.6 (CH_3), 51.1 (C-1'')

IR (KBr): ṽ (cm^{-1}) = 3054, 3028, 2948, 2834, 1716, 1634, 1611, 1598, 1494, 1462, 1431, 1381, 1280, 1177, 1106, 1014, 937, 875, 746, 670, 542

HRMS (ESI): m/z = 344.1281 $[M+H]^+$ calculated for $C_{22}H_{18}NO_3^+$ 344.1281

4-{[9-Oxoacridin-10(9*H*)-yl]methyl}benzoic acid (KV-47)

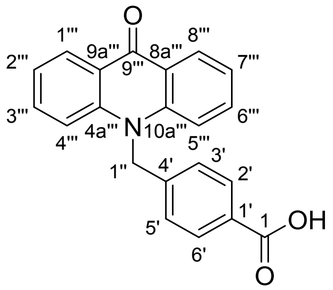

$C_{21}H_{15}NO_3$

M_r = 329.36

The synthesis was performed according to General procedure 3. Therefore, 250 mg (0.728 mmol) methyl 4-{[9-oxoacridin-10(9*H*)-yl]methyl}benzoate (**KV-42**) and 910 µL (1.82 mmol) 2 M NaOH solution were used in 5.0 mL of a mixture of dioxane/water (1:1). After a reaction time of 88 h, the reaction mixture was acidified with 1 M citric acid. The precipitated product was collected by filtration and dried under high vacuum resulting in 163 mg (yield 68 %) of a light-yellow solid as product.

M.p.: 317 – 318 °C

^{1}H NMR (500 MHz, DMSO-*d*$_6$): δ (ppm) = 12.91 (s, 1H, OH), 8.39 (dd, *J* = 8.0, 1.7 Hz, 2H, 1'''-H, 8'''-H), 7.98 – 7.85 (m, 2H, 2'-H, 6'-H), 7.76 (ddd, *J* = 8.7, 6.9, 1.7 Hz, 2H, 3'''-H, 6'''-H), 7.62 (d, *J* = 8.7 Hz, 2H, 4'''-H, 5'''-H), 7.35 (t, *J* = 7.4 Hz, 2H, 2'''-H, 7'''-H), 7.31 – 7.23 (m, 2H, 3'-H, 5'-H), 5.89 (s, 2H, 1''-H)

^{13}C NMR (126 MHz, DMSO-*d*$_6$): δ (ppm) = 176.7 (C-9'''), 167.0 (C-1), 142.0 (C-4a''', C-10a'''), 141.6 (C-4'), 134.4 (C-3''', C-6'''), 129.9 (C-2', C-6'), 129.9 (C-1'), 126.8 (C-1''', C-8'''), 126.1 (C-3', C-5'), 121.7 (C-8a''', C-9a'''), 121.7 (C-2''', C-7'''), 116.1 (C-4''', C-5'''), 49.0 (C-1'')

IR (KBr): ṽ (cm^{-1}) = 3070, 2913, 2619, 2497, 1709, 1609, 1591, 1496, 1461, 1419, 1379, 1292, 1267, 1234, 1177, 1111, 937, 872, 754, 674, 548

HRMS (ESI): m/z = 330.1124 [M+H]$^+$ calculated for $C_{21}H_{16}NO_3^+$ 330.1125

(±)-4-{[9-Oxoacridin-10(9*H*)-yl]methyl}-*N*-[(tetrahydro-2*H*-pyran-2-yl)oxy]benzamide (KV-48)

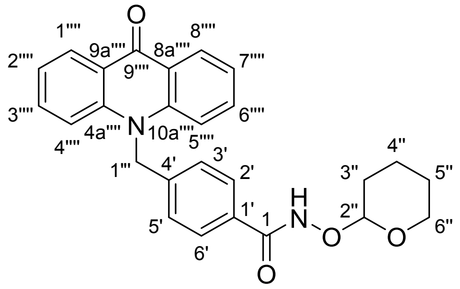

$C_{26}H_{24}N_2O_4$

M_r = 428.49

Synthesis was performed according to General procedure 1. Therefore, 110 mg (0.334 mmol) 4-{[9-oxoacridin-10(9*H*)-yl]methyl}benzoic acid (**KV-47**) in 1.5 mL dry DMF, 110 µL (0.647 mmol) *N*,*N*-diisopropylethylamine, 220 mg (0.514 mmol) COMU in 1.0 mL dry DMF and 59 mg (0.50 mmol) *O*-(tetrahydro-2*H*-pyran-2-yl)hydroxylamine in 1.0 mL dry DMF were used. After 72 h, the mixture was extracted three times with DCM (10 mL each). Purification by FCC (DCM/MeOH = 98:2, R_f = 0.3) resulted in 69 mg (yield 48 %) of a light-yellow solid.

M.p.: 224 °C

^{1}H NMR (500 MHz, DMSO-*d*$_6$): δ (ppm) = 11.58 (s, 1H, NH), 8.39 (d, *J* = 7.9 Hz, 2H, 1''''-H, 8''''-H), 7.75 (t, *J* = 8.0 Hz, 2H, 3''''-H, 6''''-H), 7.73 – 7.68 (m, 2H, 2'-H, 6'-H), 7.61 (d, *J* = 8.8 Hz, 2H, 4''''-H, 5''''-H), 7.35 (t, *J* = 7.6 Hz, 2H, 2''''-H, 7''''-H), 7.28 – 7.21 (m, 2H, 3'-H, 5'-H), 5.87 (s, 2H, 1'''-H), 4.96 (s, 1H, 2''-H), 4.10 – 3.93 (m, 1H, 6''-H_a), 3.55 – 3.42 (m, 1H, 6''-H_e), 1.77 – 1.63 (m, 3H, 3''-H_a, 3''-H_e, 4''-H_a/H_e), 1.61 – 1.45 (m, 3H, 4''-H_a/H_e, 5''-H_a, 5''-H_e)

^{13}C NMR (126 MHz, DMSO-*d*$_6$): δ (ppm) = 176.7 (C-9''''), 164.0 (C-1), 142.1 (C-4a'''', C-10a''''), 140.1 (C-4'), 134.4 (C-3'''', C-6''''), 131.5 (C-1'), 127.8 (C-2', C-6'), 126.7 (C-1'''', C-8''''), 125.9 (C-3', C-5'), 121.7 (C-8a'''', C-9a''''), 121.7 (C-2'''', C-7''''), 116.1 (C-4'''', C-5''''), 101.0 (C-2''), 61.4 (C-6''), 48.9 (C-1'''), 27.9 (C-3''), 24.7 (C-5''), 18.3 (C-4'')

IR (KBr): ṽ (cm^{-1}) = 3242, 3069, 2943, 2871, 1676, 1631, 1607, 1575, 1492, 1461, 1378, 1292, 1267, 1178, 1112, 1037, 936, 903, 870, 755, 674, 545

HRMS (ESI): m/z = 429.1806 $[M+H]^+$ calculated for $C_{26}H_{25}N_2O_4^+$ 429.1809

_N_-Hydroxy-4-{[9-oxoacridin-10(9_H_)-yl]methyl}benzamide (KV-49)

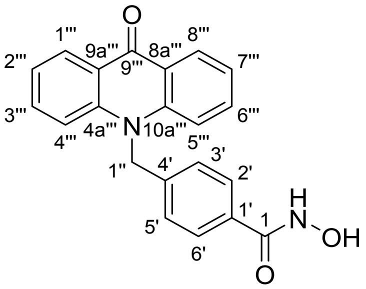

$C_{21}H_{16}N_2O_3$

M_r = 344.37

Synthesis was performed according to General procedure 2. Therefore, 40 mg (0.093 mmol) (±)-4-{[9-oxoacridin-10(9*H*)-yl]methyl}-*N*-[(tetrahydro-2*H*-pyran-2-yl)oxy]benzamide (**KV-48**) in 2.0 mL DCM and 0.12 mL (0.48 mmol) 4 M HCl in 1,4-dioxane were used. The reaction mixture was stirred for 5 h and resulted in 26 mg (yield 79 %) of a light-yellow solid as product.

M.p.: 232 °C

^{1}H NMR (500 MHz, DMSO-*d*$_6$): δ (ppm) = 11.14 (s, 1H, NHO*H*), 9.00 (bs, 1H, N*H*OH), 8.39 (dd, *J* = 8.0, 1.7 Hz, 2H, 1'''-H, 8'''-H), 7.76 (ddd, *J* = 8.7, 6.8, 1.7 Hz, 2H, 3'''-H, 6'''-H), 7.72 – 7.67 (m, 2H, 2'-H, 6'-H), 7.62 (d, *J* = 8.7 Hz, 2H, 4'''-H, 5'''-H), 7.35 (t, *J* = 7.4 Hz, 2H, 2'''-H, 7'''-H), 7.25 – 7.20 (m, 2H, 3'-H, 5'-H), 5.86 (s, 2H, 1''-H)

^{13}C NMR (126 MHz, DMSO-*d*$_6$): δ (ppm) = 176.7 (C-9'''), 163.9 (C-1), 142.0 (C-4a''', C-10a'''), 139.6 (C-4'), 134.4 (C-3''', C-6'''), 132.0 (C-1'), 127.5 (C-2', C-6'), 126.7 (C-1''', C-8'''), 125.8 (C-3', C-5'), 121.7 (C-8a''', C-9a'''), 121.6 (C-2''', C-7'''), 116.1 (C-4''', C-5'''), 48.9 (C-1'')

IR (KBr): ṽ (cm^{-1}) = 3201, 3070, 2856, 1610, 1593, 1571, 1497, 1461, 1379, 1292, 1270, 1180, 1014, 937, 896, 753, 674, 546

HRMS (ESI): m/z = 343.1087 [M-H]$^-$ calculated for $C_{21}H_{15}N_2O_3^-$ 343.1088

Methyl 4-{[2-(1*H*-indol-2-yl)phenyl]carbamoyl}benzoate (KV-22)

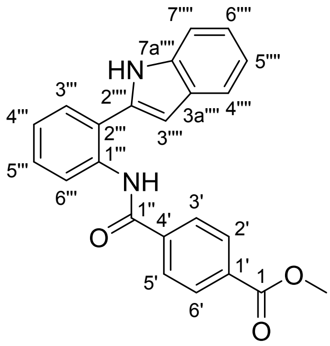

$C_{23}H_{18}N_2O_3$

M_r = 370.41

After suspending 900 mg (4.32 mmol) 2-(1*H*-indol-2-yl)aniline and 909 mg (4.58 mmol) methyl 4-(chlorocarbonyl)benzoate in 15 mL toluene, the reaction mixture was cooled to 0 °C. Then, 1.23 mL (15.2 mmol) pyridine were added slowly. After addition of 28 mg (0.23 mmol) 4-(dimethylamino)pyridine, the reaction mixture was stirred at room temperature for 1 h. The solvent was evaporated, and the crude product was purified by FCC (iso-hexane/ethyl acetate = 8:2, R_f = 0.3 and DCM/MeOH = 99:1, R_f = 0.3) resulting in 1.33 g (yield 83 %) of a light-orange solid as product.

M.p.: 142 – 144 °C

^{1}H NMR (500 MHz, CD_2Cl_2): δ (ppm) = 8.81 (s, 2H, CONH, NH), 8.47 (d, *J* = 8.2 Hz, 1H, 6'''-H), 8.06 – 7.98 (m, 2H, 2'-H, 6'-H), 7.83 – 7.75 (m, 2H, 3'-H, 5'-H), 7.65 (dd, *J* = 8.0, 1.0 Hz, 1H, 4''''-H), 7.53 (dd, *J* = 7.7, 1.6 Hz, 1H, 3'''-H), 7.47 (dd, *J* = 8.1, 1.0 Hz, 1H, 7''''-H), 7.41 (ddd, *J* = 8.6, 7.6, 1.6 Hz, 1H, 5'''-H), 7.25 (td, *J* = 7.5, 1.2 Hz, 1H, 4'''-H), 7.25 (ddd, *J* = 8.2, 7.0, 1.1 Hz, 1H, 6''''-H), 7.16 (ddd, *J* = 8.0, 7.1, 1.0 Hz, 1H, 5''''-H), 6.71 (dd, *J* = 2.1, 0.9 Hz, 1H, 3''''-H), 3.90 (s, 3H, CH_3)

^{13}C NMR (126 MHz, CD_2Cl_2): δ (ppm) = 166.5 (C-1), 164.9 (C-1''), 138.9 (C-4'), 137.4 (C-7a''''), 135.8 (C-1'''), 134.6 (C-2''''), 133.6 (C-1'), 130.4 (C-2', C-6'), 129.9 (C-3'''), 129.7 (C-5'''), 129.4 (C-3a''''), 127.6 (C-3', C-5'), 125.4 (C-4'''), 124.4 (C-2'''), 123.4 (C-6''''), 122.2 (C-6'''), 121.2 (C-4''''), 121.0 (C-5''''), 111.7 (C-7''''), 103.0 (C-3''''), 52.8 (CH_3)

IR (KBr): ṽ (cm^{-1}) = 3377, 3059, 2951, 2844, 1724, 1663, 1583, 1524, 1439, 1283, 1192, 1110, 1017, 870, 823, 792, 750, 724

HRMS (ESI): m/z = 369.1247 [M-H]$^-$ calculated for $C_{23}H_{17}N_2O_3^-$ 369.1245

Methyl 4-(11*H*-indolo[3,2-c]quinolin-6-yl)benzoate (KV-25)

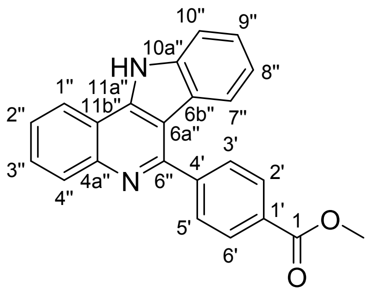

$C_{23}H_{16}N_2O_2$

M_r = 352.39

In a microwave tube, 1.01 g (2.73 mmol) methyl 4-{[2-(1*H*-indol-2-yl)phenyl]carbamoyl}benzoate (**KV-22**) and 300 µL (3.20 mmol) 2-chloropyridine were dissolved in 8.0 mL DCM and cooled down to - 78 °C under stirring. Then, 500 µL (2.97 mmol) trifluoromethanesulfonic anhydride were added dropwise and the mixture was warmed up to 0 °C after stirring for 5 min. The cooling bath was removed after another 5 min and the reaction mixture was warmed up to room temperature. Afterwards, the vial was placed in a microwave reactor and heated up for 5 min to 140 °C (300 W, max. pressure 145 psi). The mixture was cooled down to room temperature and neutralised with triethylamine. After evaporation of the solvent, the crude product was purified by FCC (DCM/MeOH = 98:2, R_f = 0.2) and resulted in 690 mg (yield 72 %) of a white solid as product.

M.p.: 272 – 273 °C

^{1}H NMR (500 MHz, DMSO-*d*$_6$): δ (ppm) = 12.97 (s, 1H, NH), 8.59 (dd, *J* = 8.1, 1.5 Hz, 1H, 1''-H), 8.28 – 8.18 (m, 2H, 2'-H, 6'-H), 8.15 (dd, *J* = 8.2, 1.3 Hz, 1H, 4''-H), 8.03 – 7.95 (m, 2H, 3'-H, 5'-H), 7.78 (ddd, *J* = 8.4, 6.9, 1.5 Hz, 1H, 3''-H), 7.74 (dd, *J* = 8.1, 0.9 Hz, 1H, 10''-H), 7.73 (ddd, *J* = 8.2, 7.0, 1.3 Hz, 1H, 2''-H), 7.48 (d, *J* = 8.1 Hz, 1H, 7''-H), 7.46 (ddd, *J* = 8.2, 7.2, 1.2 Hz, 1H, 9''-H), 7.15 (ddd, *J* = 8.1, 7.3, 0.9 Hz, 1H, 8''-H), 3.94 (s, 3H, CH_3)

^{13}C NMR (126 MHz, DMSO-*d*$_6$): δ (ppm) = 166.1 (C-1), 154.3 (C-6''), 145.2 (C-4'), 144.9 (C-4a''), 141.1 (C-11a''), 139.1 (C-10a''), 129.9 (C-1'), 129.5 (C-4''), 129.4 (C-2', C-6'), 129.4 (C-3', C-5'), 128.7 (C-3''), 126.0 (C-2''), 125.6 (C-9''), 122.0 (C-1''), 121.4 (C-6b''), 121.0 (C-7''), 120.5 (C-8''), 116.4 (C-11b''), 112.1 (C-10''), 111.8 (C-6a''), 52.4 (CH_3)

IR (KBr): ṽ (cm^{-1}) = 3431, 3055, 2946, 2883, 2844, 2795, 1724, 1559, 1517, 1502, 1458, 1437, 1360, 1272, 1241, 1112, 1100, 1018, 952, 776, 753, 745, 713, 619

HRMS (ESI): m/z = 351.1140 $[M-H]^-$ calculated for $C_{23}H_{15}N_2O_2^-$ 351.1139

4-(11*H*-Indolo[3,2-c]quinolin-6-yl)benzoic acid (KV-26)

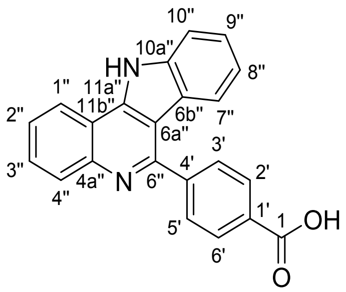

$C_{22}H_{14}N_2O_2$

M_r = 338.37

The synthesis was performed according to General procedure 3. Therefore, 240 mg (0.681 mmol) methyl 4-(11*H*-indolo[3,2-c]quinolin-6-yl)benzoate (**KV-25**) and 850 µL (1.70 mmol) 2 M NaOH solution were used in 10 mL of a mixture of dioxane/water (1:1). After a reaction time of 41 h, the reaction mixture was acidified with 1 M citric acid. The precipitated product was collected by filtration, washed with citric acid and dried under high vacuum resulting in 220 mg (yield 96 %) of a light-yellow solid as product.

M.p.: 409 – 410 °C

^{1}H NMR (400 MHz, DMSO-*d*$_6$): δ (ppm) = 12.96 (s, 2H, NH, OH), 8.59 (dd, *J* = 8.0, 1.6 Hz, 1H, 1''-H), 8.25 – 8.18 (m, 2H, 2'-H, 6'-H), 8.15 (dd, *J* = 8.2, 1.3 Hz, 1H, 4''-H), 8.00 – 7.93 (m, 2H, 3'-H, 5'-H), 7.78 (ddd, *J* = 8.4, 7.0, 1.5 Hz, 1H, 3''-H), 7.74 (d, *J* = 8.2 Hz, 1H, 10''-H), 7.73 (ddd, *J* = 8.1, 6.9, 1.3 Hz, 1H, 2''-H), 7.49 (d, *J* = 8.2 Hz, 1H, 7''-H), 7.46 (ddd, *J* = 8.2, 7.3, 1.1 Hz, 1H, 9''-H), 7.16 (ddd, *J* = 8.1, 7.2, 0.8 Hz, 1H, 8''-H)

^{13}C NMR (101 MHz, DMSO-*d*$_6$): δ (ppm) = 167.2 (C-1), 154.4 (C-6''), 144.9 (C-4a''), 144.8 (C-4'), 141.1 (C-11a''), 139.1 (C-10a''), 131.1 (C-1'), 129.5 (C-2', C-6'), 129.4 (C-4''), 129.1 (C-3', C-5'), 128.6 (C-3''), 125.9 (C-2''), 125.5 (C-9''), 122.0 (C-1''), 121.4 (C-6b''), 121.0 (C-7''), 120.4 (C-8''), 116.3 (C-11b''), 112.0 (C-10''), 111.8 (C-6a'')

IR (KBr): ṽ (cm^{-1}) = 3401, 3054, 2942, 2885, 2760, 2635, 1705, 1583, 1536, 1446, 1372, 1223, 1173, 1111, 1017, 905, 786, 742, 689, 618, 548

HRMS (ESI): m/z = 337.0982 [M-H]$^-$ calculated for $C_{22}H_{13}N_2O_2^-$ 337.0983

(±)-4-(11*H*-Indolo[3,2-*c*]quinolin-6-yl)-*N*-[(tetrahydro-2*H*-pyran-2-yl)oxy]benzamide (KV-45)

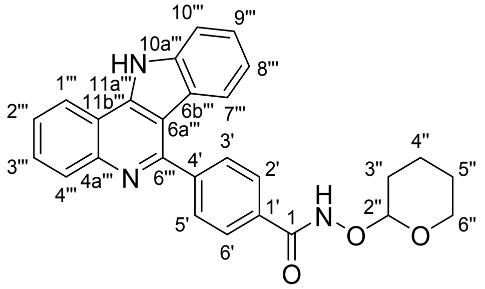

$C_{27}H_{23}N_3O_3$

M_r = 437.50

Synthesis was performed according to General procedure 1. Therefore, 102 mg (0.301 mmol) 4-(11*H*-indolo[3,2-c]quinolin-6-yl)benzoic acid (**KV-26**) in 1.5 mL dry DMF, 100 µL (0.588 mmol) *N*,*N*-diisopropylethylamine, 190 mg (0.444 mmol) COMU in 1.0 mL dry DMF and 53 mg (0.45 mmol) *O*-(tetrahydro-2*H*-pyran-2-yl)hydroxylamine in 1.0 mL dry DMF were used. After 17 h, the mixture was extracted three times with DCM (10 mL each). Purification by FCC (DCM/MeOH/0.1 % triethylamine = 92:8:0.1, R_f = 0.5) resulted in 78 mg (yield 60 %) of a white solid as product.

M.p.: 243 °C

^{1}H NMR (400 MHz, DMSO-*d*$_6$): δ (ppm) = 12.94 (s, 1H, NH), 11.85 (s, 1H, CONH), 8.59 (dd, *J* = 8.1, 1.5 Hz, 1H, 1'''-H), 8.15 (dd, *J* = 8.3, 1.2 Hz, 1H, 4'''-H), 8.07 – 8.01 (m, 2H, 2'-H, 6'-H), 7.97 – 7.91 (m, 2H, 3'-H, 5'-H), 7.79 (ddd, *J* = 8.4, 6.9, 1.5 Hz, 1H, 3'''-H), 7.74 (d, *J* = 8.1 Hz, 1H, 10'''-H), 7.73 (ddd, *J* = 8.2, 6.9, 1.3 Hz, 1H, 2'''-H), 7.51 (d, *J* = 8.1 Hz, 1H, 7'''-H), 7.46 (ddd, *J* = 8.2, 7.2, 1.1 Hz, 1H, 9'''-H), 7.16 (ddd, *J* = 8.0, 7.3, 0.9 Hz, 1H, 8'''-H), 5.09 (t, *J* = 2.3 Hz, 1H, 2''-H), 4.21 – 4.03 (m, 1H, 6''-H_a), 3.64 – 3.51 (m, 1H, 6''-H_e), 1.87 – 1.70 (m, 3H, 3''-H_a, 3''-H_e, 4''-H_a/H_e), 1.68 – 1.51 (m, 3H, 4''-H_a/H_e, 5''-H_a, 5''-H_e)

^{13}C NMR (101 MHz, DMSO-*d*$_6$): δ (ppm) = 164.0 (C-1), 154.5 (C-6'''), 144.9 (C-4a'''), 143.6 (C-4'), 141.1 (C-11a'''), 139.1 (C-10a'''), 132.6 (C-1'), 129.4 (C-4'''), 129.0 (C-3', C-5'), 128.6 (C-3'''), 127.4 (C-2', C-6'), 125.9 (C-2'''), 125.5 (C-9'''), 122.0 (C-1'''), 121.4 (C-6b'''), 121.0 (C-7'''), 120.4 (C-8'''), 116.3 (C-11b'''), 112.0 (C-10'''), 111.8 (C-6a'''), 101.0 (C-2''), 61.4 (C-6''), 27.9 (C-3''), 24.8 (C-5''), 18.3 (C-4'')

IR (KBr): ṽ (cm^{-1}) = 3441, 3263, 3053, 2942, 2869, 1663, 1638, 1566, 1501, 1440, 1359, 1319, 1240, 1205, 1112, 1022, 951, 897, 872, 767, 741

HRMS (ESI): m/z = 436.1662 $[M-H]^-$ calculated for $C_{27}H_{22}N_3O_3^-$ 436.1667

6-(4-(Hydroxycarbamoyl)phenyl)-11*H*-indolo[3,2-*c*]quinolin-5-ium chloride (KV-50)

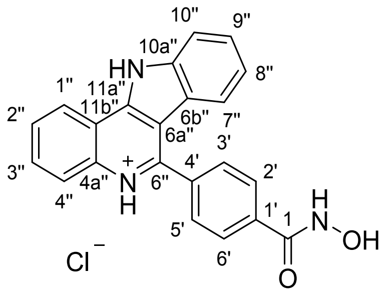

$C_{22}H_{16}ClN_3O_2$

M_r = 389.84

Synthesis was performed according to General procedure 2. Therefore, 59 mg (0.13 mmol) (±)-4-(11*H*-indolo[3,2-*c*]quinolin-6-yl)-*N*-[(tetrahydro-2*H*-pyran-2-yl)oxy]benzamide (**KV-45**) in 3.0 mL DCM and 40 µL (0.16 mmol) 4 M HCl in 1,4-dioxane were used. The reaction mixture was stirred for 5 h and resulted in 37 mg (yield 73 %) of a light-yellow solid as product.

M.p.: 264 °C

^{1}H NMR (400 MHz, DMSO-*d*$_6$): δ (ppm) = 15.41 (bs, 1H, NH$^+$), 14.77 (bs, 1H, NH), 11.62 (bs, 1H, NHO*H*), 9.30 (bs, 1H, N*H*OH), 9.00 (d, *J* = 8.1 Hz, 1H, 1''-H), 8.44 (d, *J* = 8.4 Hz, 1H, 4''-H), 8.20 – 8.14 (m, 2H, 2'-H, 6'-H), 8.14 – 8.07 (m, 3H, 3'-H, 5'-H, 3''-H), 8.01 (t, *J* = 7.6 Hz, 1H, 2''-H), 7.92 (d, *J* = 8.2 Hz, 1H, 10''-H), 7.65 (ddd, *J* = 8.2, 6.8, 1.4 Hz, 1H, 9''-H), 7.36 (t, *J* = 8.0 Hz, 1H, 8''-H), 7.32 (d, *J* = 7.7 Hz, 1H, 7''-H)

^{13}C NMR (101 MHz, DMSO-*d*$_6$): δ (ppm) = 163.2 (C-1), 150.4 (C-6''), 144.1 (C-11a''), 140.4 (C-10a''), 135.6 (C-4a''), 133.9 (C-4'), 132.3 (C-3''), 129.8 (C-1', C-3', C-5'), 128.2 (C-2''), 127.9 (C-9''), 127.6 (C-2', C-6'), 123.7 (C-1''), 122.9 (C-8''), 121.5 (C-6b''), 121.2 (C-4''), 120.9 (C-7''), 115.2 (C-11b''), 113.4 (C-10''), 112.1 (C-6a'')

IR (KBr): ṽ (cm^{-1}) = 3419, 3053, 2941, 2873, 2783, 1640, 1586, 1557, 1445, 1404, 1367, 1242, 1220, 1155, 1015, 894, 763, 711, 619, 569

HRMS (ESI): m/z = 354.1232 [M-Cl]$^+$ calculated for $C_{22}H_{16}N_3O_2^+$ 354.1237

4-[(10*H*-Phenothiazin-10-yl)methyl]benzonitrile (KV-77)

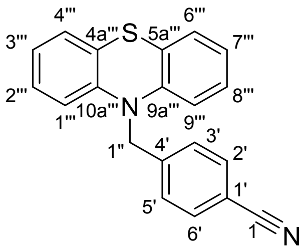

$C_{20}H_{14}N_2S$

M_r = 314.41

Under N_2 atmosphere, 1.00 g (5.02 mmol) 10*H*-phenothiazine were dissolved in 5.0 mL dry DMF and cooled to 0 °C. Then, 403 mg (10.1 mmol) of a sodium hydride dispersion (60 % in mineral oil) were added. After stirring for 30 min, a solution of 1.40 g (7.14 mmol) 4-(bromomethyl)benzonitrile in 5.0 mL dry DMF was added dropwise. The mixture was stirred for 24 h at room temperature. Afterwards, the reaction mixture was poured into water and extracted four times with DCM (60 mL each). The combined organic phases were washed with brine and dried over magnesium sulfate. After evaporation of the solvent, the crude product was purified by FCC (DCM/*n*-pentane = 1:1, R_f = 0.4) and resulted in 1.07 g (yield 68 %) of an off-white solid as product.

M.p.: 56 – 58 °C

^{1}H NMR (400 MHz, CD_2Cl_2): δ (ppm) = 7.65 – 7.59 (m, 2H, 2'-H, 6'-H), 7.51 – 7.45 (m, 2H, 3'-H, 5'-H), 7.13 (dd, *J* = 7.6, 1.6 Hz, 2H, 4'''-H, 6'''-H), 7.02 (td, *J* = 7.8, 1.6 Hz, 2H, 2'''-H, 8'''-H), 6.91 (td, *J* = 7.5, 1.2 Hz, 2H, 3'''-H, 7'''-H), 6.62 (dd, *J* = 8.1, 1.1 Hz, 2H, 1'''-H, 9'''-H), 5.14 (s, 2H, 1''-H)

^{13}C NMR (101 MHz, CD_2Cl_2): δ (ppm) = 144.8 (C-9a''', C-10a'''), 143.3 (C-4'), 133.1 (C-2', C-6'), 128.2 (C-3', C-5'), 127.8 (C-2''', C-8'''), 127.6 (C-4''', C-6'''), 124.5 (C-4a''', C-5a'''), 123.4 (C-3''', C-5'''), 119.2 (C-1), 116.0 (C-1''', C-9'''), 111.6 (C-1'), 52.5 (C-1'')

IR (KBr): ṽ (cm^{-1}) = 3058, 2920, 2851, 2226, 1569, 1463, 1443, 1365, 1286, 1255, 1217, 1127, 1038, 864, 814, 747, 547

HRMS (ESI): m/z = 315.0951 $[M+H]^+$ calculated for $C_{20}H_{15}N_2S^+$ 315.0950

4-[(10*H*-Phenothiazin-10-yl)methyl)phenyl]methanamine (KV-80)

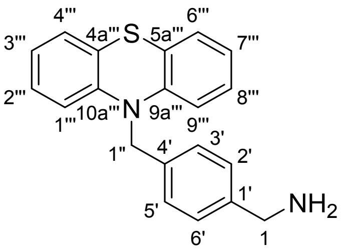

Literature known substance as hydrochloride[30]

$C_{20}H_{18}N_2S$

M_r = 318.44

In 140 mL of a mixture of MeOH/THF (5:2) 603 mg (1.92 mmol) 4-[(10*H*-phenothiazin-10-yl)methyl]benzonitrile (**KV-77**) and 912 mg (3.83 mmol) cobalt(II) chloride hexahydrate were suspended. Then, 741 mg (19.6 mmol) sodium borohydride were added carefully, and the mixture was stirred at room temperature for 1.5 h. Afterwards, 40 mL 2 M HCl were added and after stirring for another 15 min, the organic solvents were evaporated. The solution was basified with 2 M NaOH to pH 10 and then extracted with DCM three times (50 mL each). The combined organic phases were washed with water and dried over magnesium sulfate. After evaporation of the solvent, the crude product was purified by FCC (DCM/MeOH/0.1 % triethylamine = 92:8:0.1, R_f = 0.2) and resulted in 272 mg (yield 45 %) of a white solid as product.

M.p.: 206 – 209 °C

^{1}H NMR (400 MHz, DMSO-*d*$_6$): δ (ppm) = 8.43 (s, 2H, NH_2), 7.49 – 7.41 (m, 2H, 2'-H, 6'-H), 7.40 – 7.31 (m, 2H, 3'-H, 5'-H), 7.15 (dd, *J* = 7.6, 1.5 Hz, 2H, 4'''-H, 6'''-H), 7.07 (td, *J* = 7.8, 1.6 Hz, 2H, 2'''-H, 8'''-H), 6.91 (td, *J* = 7.5, 1.1 Hz, 2H, 3'''-H, 7'''-H), 6.79 (dd, *J* = 8.2, 0.8 Hz, 2H, 1'''-H, 9'''-H), 5.15 (s, 2H, 1''-H), 3.95 (s, 2H, 1-H)

^{13}C NMR (101 MHz, DMSO-*d*$_6$): δ (ppm) = 144.2 (C-9a''', C-10a'''), 137.3 (C-4'), 132.7 (C-1'), 129.2 (C-2', C-6'), 127.5 (C-2''', C-8'''), 126.9 (C-3', C-5'), 126.9 (C-4''', C-6'''), 122.7 (C-3''', C-4a''', C-5a''', C-7'''), 115.8 (C-1''', C-9'''), 50.6 (C-1''), 41.8 (C-1)

IR (KBr): ṽ (cm^{-1}) = 3423, 3008, 2894, 1594, 1573, 1464, 1443, 1368, 1285, 1255, 1219, 1128, 1049, 864, 751

HRMS (ESI): m/z = 319.1261 $[M+H]^+$ calculated for $C_{20}H_{19}N_2S^+$ 319.1263

Ethyl {4-[(10*H*-phenothiazin-10-yl)methyl]benzyl}carbamate (KV-83)

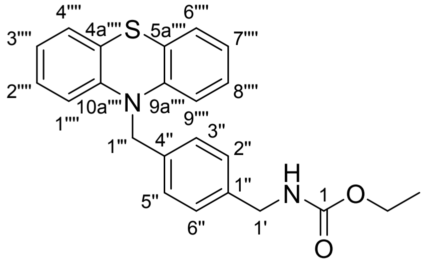

$C_{23}H_{22}N_2O_2S$

M_r = 390.50

A solution of 112 mg (0.351 mmol) 4-[(10*H*-phenothiazin-10-yl)methyl)phenyl]methanamine (KV-80) in 2.0 mL DCM was cooled to 0 °C. After addition of 180 µL (1.05 mmol) *N*,*N*-diisopropylethylamine and 87 µL (0.91 mmol) ethyl chloroformate, the reaction mixture was stirred for 1.5 h at room temperature. Then, 20 mL 2 M NaOH were added and the mixture was extracted three times with DCM (20 mL each). The combined organic phases were dried over sodium sulfate and the solvent was evaporated. The crude product was purified by FCC (DCM/n-pentane = 9:1, *Rf* = 0.3) and resulted in 132 mg (yield 96 %) of a white solid as product.

M.p.: 107 °C

^{1}H NMR (400 MHz, CD_2Cl_2): δ (ppm) = 7.33 – 7.27 (m, 2H, 3''-H, 5''-H), 7.26 – 7.21 (m, 2H, 2''-H, 6''-H), 7.09 (dd, *J* = 7.6, 1.6 Hz, 2H, 4''''-H, 6''''-H), 7.00 (ddd, *J* = 8.2, 7.4, 1.6 Hz, 2H, 2''''-H, 8''''-H), 6.87 (t, *J* = 7.4 Hz, 2H, 3''''-H, 7''''-H), 6.67 (dd, *J* = 8.2, 1.1 Hz, 2H, 1''''-H, 9''''-H), 5.08 (s, 3H, 1'''-H, NH), 4.31 (d, *J* = 6.1 Hz, 2H, 1'-H), 4.10 (q, *J* = 7.1 Hz, 2H, $C\underline{H_2}CH_3$), 1.22 (t, *J* = 7.1 Hz, 3H, $CH_2C\underline{H_3}$)

^{13}C NMR (101 MHz, CD_2Cl_2): δ (ppm) = 157.1 (C-1), 145.2 (C-9a'''', C-10a''''), 138.5 (C-1''), 136.5 (C-4''), 128.1 (C-2'', C-6''), 127.7 (C-2'''', C-8''''), 127.5 (C-3'', C-5''), 127.4 (C-4'''', C-6''''), 124.0 (C-4a'''', C-5a''''), 123.1 (C-3'''', C-7''''), 116.1 (C-1'''', C-9''''), 61.4 ($\underline{C}H_2CH_3$), 52.6 (C-1'''), 45.0 (C-1'), 15.0 ($CH_2\underline{C}H_3$)

IR (KBr): ṽ (cm^{-1}) = 3303, 3062, 3013, 2980, 2933, 2902, 2854, 1685, 1550, 1462, 1421, 1373, 1326, 1264, 1139, 1038, 929, 751, 727, 674

HRMS (ESI): m/z = 391.1475 $[M+H]^+$ calculated for $C_{23}H_{23}N_2O_2S^+$ 391.1475

1-{4-[(10*H*-Phenothiazin-10-yl)methyl]benzyl}-3-ethylthiourea (KV-92)

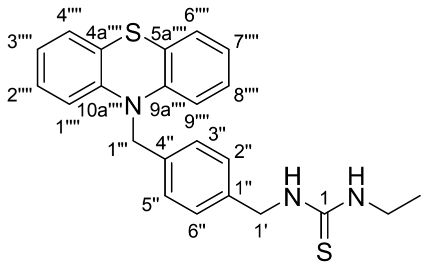

$C_{23}H_{23}N_3S_2$

M_r = 405.58

To a solution of 70 mg (0.22 mmol) 4-[(10*H*-phenothiazin-10-yl)methyl)phenyl]methanamine (**KV-80)** in 0.5 mL THF 57 µL (0.66 mmol) ethyl isothiocyanate were added dropwise and the mixture was stirred at room temperature for 16 h. Then, the mixture was poured into 20 mL water and extracted three times with DCM (20 mL each). The combined organic phases were washed with brine and dried over sodium sulfate. After evaporation of the solvent, the crude product was purified by FCC (DCM/MeOH = 99:1, *Rf* = 0.2) resulting in 53 mg (60 % yield) of a white solid as product.

M.p.: 178 – 179 °C

^{1}H NMR (400 MHz, DMSO-*d*$_6$): δ (ppm) = 7.67 (t, *J* = 5.8 Hz, 1H, N*H*CSNHCH_2CH_3), 7.37 (t, *J* = 5.0 Hz, 1H, N*H*CH_2CH_3), 7.31 – 7.26 (m, 2H, 3''-H, 5''-H), 7.26 – 7.21 (m, 2H, 2''-H, 6''-H), 7.13 (dd, *J* = 7.6, 1.5 Hz, 2H, 4''''-H, 6''''-H), 7.06 (td, *J* = 7.8, 1.6 Hz, 2H, 2''''-H, 8''''-H), 6.90 (td, *J* = 7.5, 1.2 Hz, 2H, 3''''-H, 7''''-H), 6.79 (d, *J* = 8.2 Hz, 2H, 1''''-H, 9''''-H), 5.11 (s, 2H, 1'''-H), 4.62 (d, *J* = 5.8 Hz, 2H, 1'-H), 3.38 (p, *J* = 6.9 Hz, 2H, NHC*H*$_2$CH_3), 1.07 (t, *J* = 7.2 Hz, 3H, NHCH_2C*H*$_3$)

^{13}C NMR (101 MHz, DMSO-*d*$_6$): δ (ppm) = 182.2 (C-1), 144.1 (C-9a'''', C-10a''''), 137.9 (C-1''), 135.3 (C-4''), 127.3 (C-2'''', C-8''''), 127.3 (C-2'', C-6''), 126.6 (C-4'''', C-6''''), 126.4 (C-3'', C-5''), 122.5 (C-4a'''', C-5a''''), 122.4 (C-3'''', C-7''''), 115.6 (C-1'''', C-9''''), 50.7 (C-1'''), 46.5 (C-1'), 38.1 (NH*C*H_2CH_3), 14.2 (NHCH_2*C*H_3)

IR (KBr): ṽ (cm^{-1}) = 3206, 3063, 3032, 2971, 2932, 2859, 1563, 1526, 1462, 1421, 1368, 1335, 1284, 1254, 1219, 1104, 958, 932, 802, 752, 729

HRMS (ESI): m/z = 406.1407 [M+H]$^+$ calculated for $C_{23}H_{24}N_3S_2^+$ 406.1406

Diethyl {4-[(10*H*-phenothiazin-10-yl)methyl]phenyl}phosphonate (KV-107)

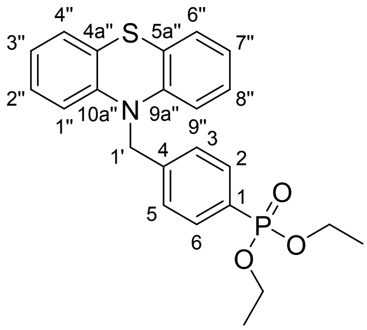

$C_{23}H_{24}NO_3PS$

M_r = 425.48

In 1.0 mL dry DMF 113 mg (0.567 mmol) 10*H*-phenothiazine were dissolved under N_2 atmosphere and cooled to 0 °C. Then, 1.13 mL (1.13 mmol) 1 M lithium bis(trimethylsilyl)amide in toluene were added dropwise. After 15 min, a solution of 296 mg (0.964 mmol) diethyl (4-(bromomethyl)benzyl)phosphonate in 0.5 mL dry DMF was added and the mixture stirred at room temperature for 18 h. Then, the mixture was poured into water and extracted three times with DCM (30 mL each). The combined organic phases were washed with brine and dried over magnesium sulfate. After evaporation of the solvent, the crude product was purified by FCC (DCM/EtOH = 97.5:2.5, *Rf* = 0.3) and resulted in 103 mg (43 % yield) of a light-brown oil as product.

^{1}H NMR (400 MHz, CD_2Cl_2): δ (ppm) = 7.78 – 7.68 (m, 2H, 2-H, 6-H), 7.48 – 7.42 (m, 2H, 3-H, 5-H), 7.11 (dd, *J* = 7.6, 1.6 Hz, 2H, 4''-H, 6''-H), 7.00 (td, *J* = 7.8, 1.6 Hz, 2H, 2''-H, 8''-H), 6.89 (td, *J* = 7.5, 1.1 Hz, 2H, 3''-H, 7''-H), 6.64 (dd, *J* = 8.1, 1.1 Hz, 2H, 1''-H, 9''-H), 5.14 (s, 2H, 1'-H), 4.17 – 3.99 (m, 4H, $C\underline{H}_2CH_3$, $C\underline{H}_2CH_3$), 1.30 (t, *J* = 7.1 Hz, 6H, $CH_2C\underline{H}_3$, $CH_2C\underline{H}_3$)

^{13}C NMR (101 MHz, CD_2Cl_2): δ (ppm) = 144.9 (C-9a'', C-10a''), 142.3 (d, $^4J_{C,P}$ = 3.2 Hz, C-4), 132.5 (d, $^2J_{C,P}$ = 10.3 Hz, C-2, C-6), 128.1 (d, $^1J_{C,P}$ = 189.8 Hz, C-1), 127.8 (C-2'', C-8''), 127.5 (C-4'', C-6''), 127.4 (d, $^3J_{C,P}$ = 15.3 Hz, C-3, C-5), 124.1 (C-4a'', C-5a''), 123.3 (C-3'', C-7''), 116.0 (C-1'', C-9''), 62.6 (d, $^2J_{C,P}$ = 5.6 Hz, $\underline{C}H_2CH_3$, $\underline{C}H_2CH_3$), 52.8 (C-1'), 16.7 (d, $^3J_{C,P}$ = 6.4 Hz, $CH_2\underline{C}H_3$, $CH_2\underline{C}H_3$)

IR (film): ṽ (cm^{-1}) = 3062, 2981, 2929, 2904, 2865, 1594, 1465, 1366, 1252, 1129, 1051, 1022, 965, 749

HRMS (ESI): m/z = 426.1290 $[M+H]^+$ calculated for $C_{23}H_{25}NO_3PS^+$ 426.1287

Ethyl hydrogen {4-[(10*H*-phenothiazin-10-yl)methyl]phenyl} phosphonate (KV-111)

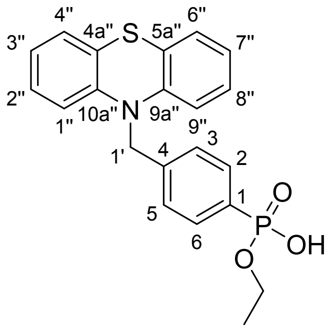

$C_{21}H_{20}NO_3PS$

M_r = 397.43

The synthesis was performed according to General procedure 3. Therefore, 68 mg (0.16 mmol) diethyl {4-[(10*H*-phenothiazin-10-yl)methyl]phenyl}phosphonate (**KV-107**) and 0.40 mL (0.80 mmol) 2 M NaOH solution were used in 0.5 mL of a mixture of dioxane/water (1:1). After a reaction time of 76 h at 70 °C, the mixture was acidified with 2 M HCl and extracted three times with DCM (30 mL each), then washed with brine, dried over magnesium sulfate and the solvent was evaporated resulting in 39 mg (yield 61 %) of a light-rose solid as product.

M.p.: 148 – 150 °C

^{1}H NMR (400 MHz, CD_2Cl_2): δ (ppm) = 7.75 – 7.65 (m, 2H, 2-H, 6-H), 7.43 – 7.35 (m, 2H, 3-H, 6-H), 7.10 (dd, *J* = 7.6, 1.6 Hz, 2H, 4''-H, 6''-H), 6.98 (td, *J* = 7.9, 1.6 Hz, 2H, 2''-H, 8''-H), 6.87 (td, *J* = 7.5, 1.2 Hz, 2H, 3''-H, 7''-H), 6.61 (dd, *J* = 8.1, 1.2 Hz, 2H, 1''-H, 9''-H), 6.28 (s, 1H, OH), 5.10 (s, 2H, 1'-H), 4.00 (qd, *J* = 7.1 Hz, $^3J_{H,P}$ = 7.1 Hz, 2H, $C\underline{H}_2CH_3$), 1.22 (t, *J* = 7.1 Hz, 3H, $CH_2C\underline{H}_3$)

^{13}C NMR (101 MHz, CD_2Cl_2): δ (ppm) = 144.9 (C-9a'', C-10a''), 142.2 (d, $^4J_{C,P}$ = 3.1 Hz, C-4), 132.2 (d, $^2J_{C,P}$ = 10.7 Hz, C-2, C-6), 128.7 (d, $^1J_{C,P}$ = 194.5 Hz, C-1), 127.8 (C-2'', C-8''), 127.4 (C-4'', C-6''), 127.3 (d, $^3J_{C,P}$ = 15.7 Hz, C-3, C-5), 124.1 (C-4a'', C-5a''), 123.2 (C-3'', C-7''), 116.0 (C-1'', C-9''), 62.5 (d, $^2J_{C,P}$ = 5.8 Hz, $\underline{C}H_2CH_3$), 52.8 (C-1'), 16.6 (d, $^3J_{C,P}$ = 6.7 Hz, $CH_2\underline{C}H_3$)

IR (KBr): ṽ (cm^{-1}) = 3061, 2979, 2924, 2853, 1593, 1464, 1367, 1256, 1219, 1130, 1041, 990, 747, 621, 523

HRMS (ESI): m/z = 398.0975 $[M+H]^+$ calculated for $C_{21}H_{21}NO_3PS^+$ 398.0974

tert-Butyl 2-{4-[(10_H_-phenothiazin-10-yl)methyl]benzoyl}hydrazine-1-carboxylate (MM-5)

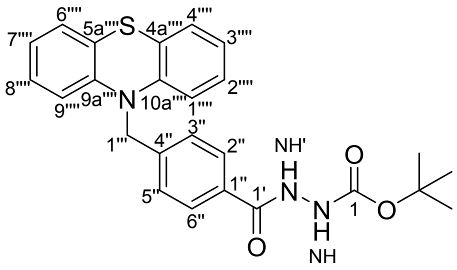

$C_{25}H_{25}N_3O_3S$

M_r = 447.55

A solution of 914 mg (2.73 mmol) 4-[(10*H*-phenothiazin-10-yl)methyl]benzoic acid[31] in 6.0 mL DMF was cooled down to 0 °C. Then, 0.47 mL (2.73 mmol) DIPEA and 1.17 g (2.73 mmol) COMU were added. The carboxylic acid was activated for 5 min at this temperature. Afterwards, 360 mg (2.73 mmol) *tert*-butyl carbazate and 0.47 mL (2.73 mmol) DIPEA were added and the mixture stirred for another 5 min at 0 °C. The ice bath was subsequently removed and stirring continued for another 16 h. The reaction mixture was quenched with water (50 mL) at 0 °C and extracted three times with diethyl ether (40 mL each). The combined organic phases were washed with saturated $NaHCO_3$ (50 mL) and brine (50 mL) and dried with sodium sulfate. Then, the solvent was removed under reduced pressure and the crude product purified by FCC (iso-hexane/ethyl acetate = 1:1, R_f = 0.4) resulting in 1.05 g (yield 86 %) of a white solid as product.

M.p.: 200 - 201 °C

^{1}H NMR (400 MHz, DMSO-*d₆*) δ (ppm) = 10.12 (d, *J* = 1.4 Hz, 1H, NH), 8.92 – 8.86 (m, 1H, NH'), 7.84 – 7.75 (m, 2H, 2''-H, 6''-H), 7.45 – 7.39 (m, 2H, 3''-H, 5''-H), 7.16 (dd, *J* = 7.6, 1.5 Hz, 2H, 4''''-H, 6''''-H), 7.07 (ddd, *J* = 8.2, 7.4, 1.6 Hz, 2H, 2''''-H, 8''''-H), 6.91 (td, *J* = 7.5, 1.1 Hz, 2H, 3''''-H, 7''''-H), 6.78 (dd, *J* = 8.2, 1.1 Hz, 2H, 1''''-H, 9''''-H), 5.20 (s, 2H, 1'''-H), 1.42 (s, 9H, $C(CH_3)_3$,)

^{13}C NMR (101 MHz, DMSO-*d₆*) δ (ppm) = 165.7 (C-1'), 155.5 (C-1), 144.1 (C-9a'''', C-10a''''), 141.1 (C-4''), 131.3 (C-1''), 127.6 (C-2'', C-6''), 127.5 (C-2'''', C-8''''), 126.9 (C-4'''', C-6''''), 126.8 (C-3'', C-5''), 122.7 (C-3'''', C-7''''), 122.6 (C-4a'''', C-5a''''), 115.8 (C-1'''', C-9''''), 79.1 (C-1'''), 50.8 ($\underline{C}(CH_3)_3$), 28.1 ($C(\underline{C}H_3)_3$)

IR (ATR): $\tilde{\nu}$ (cm^{-1}) = 3258, 1745, 1651, 1538, 1488, 1452, 1366, 1252, 1219, 1162, 1101, 1037, 1004, 875, 831, 747

HRMS (ESI): m/z = 448.1691 $[M+H]^+$ calculated for $C_{25}H_{26}N_3O_3S^+$ 448.1695

tert-Butyl 2-{4-[(10H-phenothiazin-10-yl)methyl]phenylcarbonothioyl}hydrazine-1-carboxylate (MM-6)

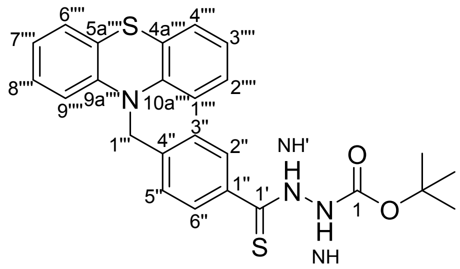

$C_{25}H_{25}N_3O_2S_2$

M_r = 463.61

Under N_2 atmosphere, 703 mg (1.56 mmol) *tert*-butyl 2-{4-[(10*H*-phenothiazine-10-yl)methyl]benzoyl}hydrazine-1-carboxylate (**MM-5**) were dissolved in 1.4 mL dry DMF in a microwave tube. To this solution 633 mg (1.56 mmol) Lawessons's reagent were added under N_2 flow. The yellow suspension was subsequently heated to 80 °C under 145 psi for 20 min in a microwave reactor. Afterwards, the solvent was removed under reduced pressure and the crude product was purified with FCC (iso-hexane/DCM = 3:7, R_f = 0.3) resulting in 406 mg (yield 56 %) of a yellow solid as product.

M.p.: 94 - 97 °C

^{1}H NMR (500 MHz, CD_2Cl_2) δ (ppm) = 9.97 (s, 1H, NH/NH'), 8.67 (s, 1H, NH/NH'), 7.70 (dd, *J* = 7.5, 1.1 Hz, 2H, 2''-H, 6''-H), 7.43 – 7.32 (m, 2H, 3''-H, 5''-H), 7.11 (dd, *J* = 7.6, 1.6 Hz, 2H, 4''''-H, 6''''-H), 7.01 (dd, *J* = 7.8, 1.5 Hz, 2H, 2''''-H, 8''''-H), 6.89 (td, *J* = 7.5, 1.1 Hz, 2H, 3''''-H, 7''''-H), 6.64 (dd, *J* = 8.1, 1.1 Hz, 2H, 1''''-H, 9''''-H), 5.11 (s, 2H, 1'''-H), 1.50 (s, 9H, $C(CH_3)_3$)

^{13}C NMR (126 MHz, CD_2Cl_2) δ (ppm) = 188.4 (C-1'), 154.3 (C-1), 144.9 (C-9a'''', C-10a''''), 141.6 (C-4''), 137.4 (C-1''), 128.0 (C-2'', C-6''), 127.8 (C-2'''', C-8''''), 127.6 (C-3'', C-5''), 127.5 (C-4'''', C-6''''), 124.1 (C-4a'''', C-5a''''), 123.2 (C-3'''', C-7''''), 116.0 (C-1'''', C-9''''), 83.7 ($\underline{C}(CH_3)_3$), 52.6 (C-1'''), 28.4 ($C(\underline{C}H_3)_3$)

IR (ATR): $\tilde{\nu}$ (cm^{-1}) = 3226, 1709, 1593, 1570, 1462, 1417, 1366, 1253, 1152, 1048, 971, 862, 744

HRMS (ESI): m/z = 464.1472 $[M+H]^+$ calculated for $C_{25}H_{26}N_3O_2S_2^+$ 464.1466

HPLC purity: 85% (λ = 210 nm), 86% (λ = 254 nm)

4-[(10*H*-Phenothiazin-10-yl)methyl]benzothiohydrazide (MM-7)

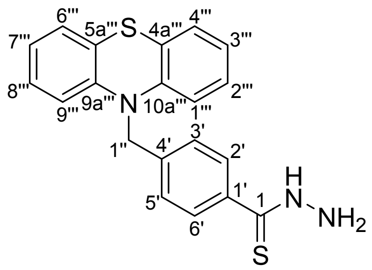

$C_{20}H_{17}N_3S_2$

M_r = 363.50

A solution of 220 mg (0.475 mmol) *tert*-butyl-2-{4-[(10*H*-phenothiazin-10-yl)methyl]phenylcarbonothioyl}hydrazine-1-carboxylate (**MM-6**) in 1.8 mL DCM was cooled down to 0 °C. Then, 1.10 mL (14.9 mmol) trifluoroacetic acid were added and the mixture was stirred at 0 °C for 1.5 h and at room temperature for 30 min. The mixture was quenched with saturated $NaHCO_3$ solution (15 mL) and extracted three times with DCM (20 mL each). The combined organic phases were dried with sodium sulfate and the solvent was removed under reduced pressure. Purification by FCC (DCM, R_f = 0.1) resulted in 72 mg (yield 41 %) of a green powder as product.

M.p.: 180 - 182 °C

^{1}H NMR (500 MHz, DMSO-*d₆*) δ (ppm) = 12.06 (s, 1H, NH), 7.75 – 7.55 (m, 2H, 2'-H, 6'-H), 7.42 – 7.28 (m, 2H, 3'-H, 5'-H), 7.15 (dd, *J* = 7.6, 1.5 Hz, 2H, 4'''-H, 6'''-H), 7.07 (ddd, *J* = 8.2, 7.4, 1.6 Hz, 2H, 2'''-H, 8'''-H), 6.91 (td, *J* = 7.5, 1.1 Hz, 2H, 3'''-H, 7'''-H), 6.79 (dd, *J* = 8.2, 1.1 Hz, 2H, 1'''-H, 9'''-H), 6.20 (s, 2H, NH_2), 5.17 (s, 2H, 1''-H)

^{13}C NMR (126 MHz, DMSO-d_6) δ (ppm) = 181.9 (C-1), 144.1 (C-9a''', C-10a'''), 139.3 (C-4'), 137.4 (C-1'), 127.5 (C-2''', C-8'''), 127.4 (C-2', C-6'), 126.9 (C-4''', C-6'''), 126.4 (C-3', C-5'), 122.7 (C-3''', C-7'''), 122.6 (C-4a''', C-5a'''), 115.8 (C-1''', C-9'''), 50.7 (C-1'')

IR (ATR): $\tilde{\nu}$ (cm^{-1}) = 3287, 3212, 3162, 3062, 2925, 1573, 1464, 1442, 1415, 1366, 1255, 1226, 1131, 1106, 1048, 969, 921, 864, 822, 761, 740

HRMS (ESI): m/z = 362.0792 $[M-H]^-$ calculated for $C_{20}H_{16}N_3S_2^-$ 362.0786

HPLC purity: decomposed

4-[(10*H*-Phenothiazin-10-yl)methyl]benzonitrile (MM-8)

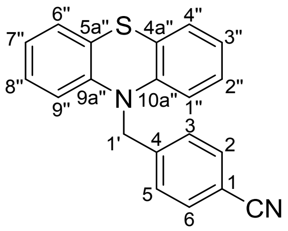

$C_{20}H_{14}N_2S$

M_r = 314.41

Under N_2 atmosphere, 300 mg (1.49 mmol) 10*H*-phenothiazine were dissolved in 1.8 mL dry DMF and cooled down to 0 °C. Then, 1.5 mL (1.5 mmol) 1 M lithium bis(trimethylsilyl)amide in toluene were added dropwise and the solution was stirred for 15 min. A solution of 497 mg (2.53 mmol) 4-(bromomethyl)benzonitrile in 1.2 mL dry DMF under N_2 atmosphere was added to the cooled phenothiazine lithium salt solution. After 15 min, the ice bath was removed and stirring continued at room temperature for 15 min and at 50 °C for 23 h. The crude mixture was quenched with water (60 mL), extracted three times with ethyl acetate (40 mL each), dried with magnesium sulfate and the solvent removed under reduced pressure. Purification by FCC (iso-hexane/ethyl acetate = 17:3, R_f = 0.3) resulted in 337 mg (yield 72 %) of a yellow solid as product.

M.p.: 58 - 60 °C

^{1}H NMR (500 MHz, CD_2Cl_2) δ (ppm) = 7.64 – 7.59 (m, 2H, 2-H, 6-H), 7.50 – 7.45 (m, 2H, 3-H, 5-H), 7.13 (dd, *J* = 7.6, 1.5 Hz, 2H, 4''-H, 6''-H), 7.02 (td, *J* = 7.8, 1.5 Hz, 2H, 2''-H, 8''-H), 6.90 (t, *J* = 7.5 Hz, 2H, 3''-H, 7''-H), 6.62 (d, *J* = 8.1 Hz, 2H, 1''-H, 9''-H), 5.14 (s, 2H, 1'-H)

^{13}C NMR (126 MHz, CD_2Cl_2) δ (ppm) = 144.8 (C-9a'', C-10a''), 143.3 (C-4), 133.1 (C-2, C-6), 128.2 (C-3, C-5), 127.8 (C-2'', C-8''), 127.6 (C-4'', C-6''), 124.5 (C-4a'', C-5a''), 123.4 (C-3'', C-7''), 119.2 (CN), 116.0 (C-1'', C-9''), 111.6 (C-1), 52.5 (C-1')

IR (ATR): $\tilde{\nu}$ (cm^{-1}) = 2226, 1592, 1569, 1462, 1444, 1365, 1332, 1286, 1255, 1217, 1129, 1105, 1049, 865, 813, 745

HRMS (ESI): m/z = 313.0806 $[M-H]^-$ calculated for $C_{20}H_{13}N_2S^-$ 313.0799

(Z)-4-[(10H-Phenothiazin-10-yl)methyl]-N'-hydroxybenzimidamide (MM-9)

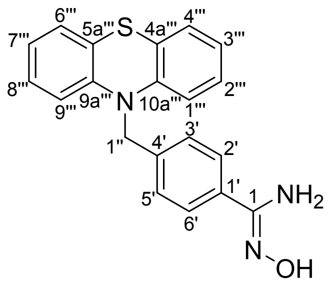

$C_{20}H_{17}N_3OS$

M_r = 347.44

A suspension of 98.0 mg (1.41 mmol) hydroxylamine hydrochloride and 119 mg (1.41 mmol) $NaHCO_3$ in 1 mL DMF was stirred for 30 min. Afterwards, 298 mg (0.942 mmol) 4-[(10*H*-phenothiazin-10-yl)methyl]benzonitrile (**MM-8**), dissolved in 1 mL DMF, were added and the mixture was stirred for 20 h. As the reaction was not completed according to TLC and most of the $NaHCO_3$ was still undissolved, more hydroxylamine hydrochloride (65.0 mg, 0.942 mmol) and DIPEA (164 µL, 0.942 mmol) were dissolved in 0.4 mL DMF, stirred for 10 minutes and subsequently added to the mixture. After altogether 67 h, the reaction was completed, the solvent was removed in vacuo, and the crude mixture purified with FCC (iso-hexane/ethyl acetate = 3:7, R_f = 0.6) to give 175 mg (yield 53 %) as a pink solid.

M.p.: 88 - 91 °C

^{1}H NMR (500 MHz, DMSO-*d₆*) δ (ppm) = 9.59 (s, 1H, OH), 7.69 – 7.56 (m, 2H, 2'-H, 6'-H), 7.36 – 7.27 (m, 2H, 3'-H, 5'-H), 7.15 (dd, *J* = 7.6, 1.5 Hz, 2H, 4'''-H, 6'''-H), 7.07 (ddd, *J* = 8.2, 7.5, 1.6 Hz, 2H, 2'''-H, 8'''-H), 6.91 (td, *J* = 7.5, 1.2 Hz, 2H, 3'''-H, 7'''-H), 6.79 (dd, *J* = 8.2, 1.1 Hz, 2H, 1'''-H, 9'''-H), 5.76 (s, 2H, NH_2), 5.14 (s, 2H, 1''-H)

^{13}C NMR (126 MHz, DMSO-d_6) δ (ppm) = 150.6 (C-1), 144.2 (C-9a''', C-10a'''), 137.7 (C-4'), 132.0 (C-1'), 127.5 (C-2''', C-8'''), 126.8 (C-4''', C-6'''), 126.5 (C-3', C-5'), 125.6 (C-2', C-6'), 122.7 (C-3''', C-7'''), 122.5 (C-4a''', C-5a'''), 115.8 (C-1''', C-9'''), 50.8 (C-1'')

IR (ATR): $\tilde{\nu}$ (cm^{-1}) = 1729, 1640, 1571, 1487, 1463, 1411, 1365, 1286, 1254, 1218, 1128, 1104, 1047, 928, 864, 747

HRMS (ESI): m/z = 348.1168 $[M + H]^+$ calculated for $C_{20}H_{18}N_3OS^+$ 348.1171

10-(4-Bromobenzyl)-10*H*-phenothiazine (MM-13)

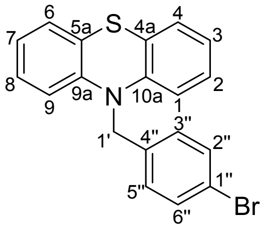

$C_{19}H_{14}BrNS$

M_r = 368.29

Under N_2 atmosphere, 1.00 g (4.97 mmol) 10*H*-phenothiazine was dissolved in 6.0 mL dry DMF and cooled down to 0 °C. Then, 5.00 mL (5.00 mmol) 1 M lithium bis(trimethylsilyl)amide in toluene were added dropwise and the mixture was stirred for 15 min. Subsequently, 2.11 g (8.45 mmol) 4-bromobenzylbromide, dissolved in 4.0 mL dry DMF, were added and the mixture stirred for 5 min at 0 °C and for 15 min at room temperature. Next, the mixture was heated to 50 °C and stirred for 68 h. The reaction mixture was allowed to cool to ambient temperature, quenched with water (60 mL) and extracted three times with DCM (40 mL each). The combined organic phases were dried with magnesium sulfate and the solvent was removed under reduced pressure. Purification by FCC (iso-hexane/DCM = 9:1, R_f = 0.2) resulted in 1.56 g (yield 85 %) of a fine, white solid as product.

M.p.: 133 °C

^{1}H NMR (400 MHz, CD_2Cl_2) δ (ppm) = 7.48 – 7.42 (m, 2H, 3''-H, 5''-H), 7.27 – 7.20 (m, 2H, 2''-H, 6''-H), 7.11 (ddd, *J* = 7.6, 1.6, 0.4 Hz, 2H, 4-H, 6-H), 7.01 (ddd, *J* = 8.2, 7.4, 1.6 Hz, 2H, 2-H, 8-H), 6.89 (td, *J* = 7.5, 1.2 Hz, 2H, 3-H, 7-H), 6.65 (ddd, *J* = 8.1, 1.2, 0.3 Hz, 2H, 1-H, 9-H), 5.04 (s, 2H, 1'-H)

^{13}C NMR (101 MHz, CD_2Cl_2) δ (ppm) = 145.0 (C-9a, C-10a), 136.7 (C-1''), 132.3 (C-3'', C-5''), 129.2 (C-2'', C-6''), 127.8 (C-2, C-8), 127.5 (C-4, C-6), 124.3 (C-4a, C-6a), 123.2 (C-3, C-7), 121.2 (C-4''), 116.1 (C-1, C-9), 52.3 (C-1')

IR (ATR): $\tilde{\nu}$ (cm^{-1}) = 1593, 1570, 1484, 1463, 1442, 1402, 1362, 1286, 1253, 1220, 1140, 1106, 1067, 1050, 1008, 926, 859, 803, 740, 656

HRMS (ESI): m/z = 368.0104 $[M + H]^+$ calculated for $C_{19}H_{15}BrNS^+$ 368.0103

5-{4-[(10*H*-Phenothiazin-10-yl)methyl]phenyl}-3-methylisoxazole (MM-16)

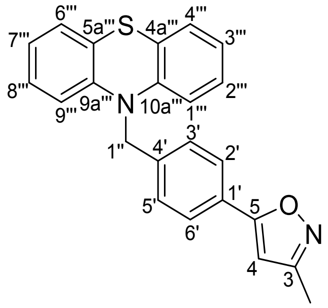

$C_{23}H_{18}N_2OS$

M_r = 370.47

A mixture of 219 mg (0.596 mmol) 10-(4-bromobenzyl)-10*H*-phenothiazine (**MM-13**), 20.9 mg (29.8 µmol) bis(triphenylphosphine)palladium(II) dichloride and 333 mg (0.894 mmol) 3-methyl-5-(tributylstannyl)isoxazole in 2.8 mL dry 1,4-dioxane was heated to 125 °C under 145 psi for 1 h in a microwave reactor. The solvent was subsequently removed under reduced pressure and FCC (iso-hexane/DCM = 1:1, R_f = 0.3) was performed. However, impurities of tributyltin bromide were not entirely removed, therefore the product was washed with distilled iso-hexane nine times. The pure title compound was obtained as a pink solid (139 mg, yield 63 %).

M.p.: 133 - 134 °C

^{1}H NMR (400 MHz, CD_2Cl_2) δ (ppm) = 7.74 – 7.68 (m, 2H, 2'-H, 6'-H), 7.47 – 7.42 (m, 2H, 3'-H, 5'-H), 7.12 (dd, *J* = 7.6, 1.5 Hz, 2H, 4'''-H, 6'''-H), 7.01 (ddd, *J* = 8.2, 7.4, 1.6 Hz, 2H, 2'''-H, 8'''-H), 6.89 (td, *J* = 7.5, 1.2 Hz, 2H, 3'''-H, 7'''-H), 6.68 (dd, *J* = 8.2, 1.2 Hz, 2H, 1'''-H, 9'''-H), 6.36 (s, 1H, 4-H), 5.13 (s, 2H, 1''-H), 2.31 (s, 3H, CH_3)

^{13}C NMR (101 MHz, CD_2Cl_2) δ (ppm) = 169.7 (C-5), 161.0 (C-3), 145.0 (C-9a''', C-10a'''), 139.7 (C-4'), 128.0 (C-3', C-5'), 127.8 (C-2''', C-8'''), 127.5 (C-4''', C-6'''), 127.1 (C-1'), 126.6 (C-2', C-6'), 124.2 (C-4a''', C-5a'''), 123.2 (C-3''', C-7'''), 116.1 (C-1''', C-9'''), 100.7 (C-4), 52.7 (C-1''), 11.8 (CH_3)

IR (ATR): $\tilde{\nu}$ (cm^{-1}) = 1618, 1589, 1568, 1516, 1484, 1451, 1417, 1370, 1327, 1284, 1255, 1218, 1103, 1034, 1018, 933, 889, 857, 826, 793, 756, 747, 732, 687, 605, 589

HRMS (ESI): m/z = 371.1214 $[M + H]^+$ calculated for $C_{23}H_{19}N_2OS^+$ 371.1218

(*Z*)-1-{4-[(10*H*-Phenothiazin-10-yl)methyl]phenyl}-3-aminobut-2-en-1-one (MM-17)

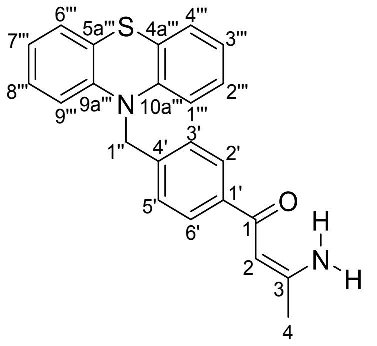

$C_{23}H_{20}N_2OS$

M_r = 372.49

To a suspension of 53.6 mg (0.145 mmol) 5-{4-[(10*H*-phenothiazin-10-yl)methyl]phenyl}-3-methylisoxazole (**MM-16**) in 1.4 mL ethanol 58 µL THF were added to increase solubility. Then, 106 mg (1.88 mmol) potassium hydroxide and 15.4 mg (0.145 mmol) palladium on charcoal (10 wt %) were added. The reaction mixture was hydrogenated with 20 bar H_2 pressure at 50 °C for 2 hours. The solvent was removed under reduced pressure and the crude product purified by FCC (DCM, R_f = 0.1) resulting in 47.6 mg (yield 88 %) of a white powder as product.

M.p.: 191 - 192 °C

^{1}H NMR (400 MHz, DMSO-*d*$_6$) δ (ppm) = 10.00 (d, *J* = 5.7 Hz, 1H, NH), 7.83 – 7.73 (m, 3H, 2'-H, 6'-H, NH), 7.39 – 7.32 (m, 2H, 3'-H, 5'-H), 7.15 (dd, *J* = 7.6, 1.5 Hz, 2H, 4'''-H, 6'''-H), 7.06 (ddd, *J* = 8.2, 7.4, 1.6 Hz, 2H, 2'''-H, 8'''-H), 6.90 (td, *J* = 7.5, 1.1 Hz, 2H, 3'''-H, 7'''-H), 6.77 (dd, *J* = 8.2, 1.1 Hz, 2H, 1'''-H, 9'''-H), 5.65 (d, *J* = 1.6 Hz, 1H, 2-H), 5.16 (s, 2H, 1''-H), 1.96 (s, 3H, 4-H)

^{13}C NMR (101 MHz, DMSO-*d*$_6$) δ (ppm) = 186.1 (C-1), 164.5 (C-3), 144.2 (C-9a''', C-10a'''), 139.6 (C-4'), 138.9 (C-1'), 127.5 (C-2''', C-8'''), 127.0 (C-2', C-6'), 126.8 (C-4''', C-6'''), 126.6 (C-3', C-5'), 122.7 (C-3''', C-7'''), 122.5 (C-4a''', C-5a'''), 115.8 (C-1''', C-9'''), 90.4 (C-2), 50.9 (C-1''), 21.8 (C-4)

IR (ATR): $\tilde{\nu}$ (cm^{-1}) = 3282, 3152, 3063, 1594, 1570, 1525, 1504, 1453, 1411, 1372, 1329, 1287, 1254, 1128, 1101, 1038, 1020, 929, 845, 786, 745, 647

HRMS (ESI): m/z = 373.1367 $[M + H]^+$ calculated for $C_{23}H_{21}N_2OS^+$ 373.1375

1-{4-[(10*H*-Phenothiazin-10-yl)methyl]phenyl}ethan-1-one (MM-20)

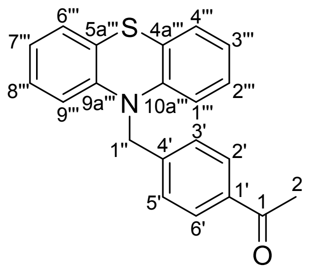

$C_{21}H_{17}NOS$

M_r = 331.43

Under N_2 atmosphere, 1.52 g (4.12 mmol) 10-(4-bromobenzyl)-10*H*-phenothiazine (**MM-13**) and 116 mg (0.165 mmol) bis(triphenylphosphine)palladium(II) dichloride were dissolved in 12 mL dry 1,4-dioxane in a microwave tube. Then, 1.53 mL (4.53 mmol) 1-(ethoxyvinyl)tributylstannane were added. The reaction mixture was heated to 125 °C under 145 psi pressure for 1 h. Afterwards, 3.09 mL (6.18 mmol) 2 M HCl were added, and the mixture was stirred for 1 h at 50 °C. Then, the mixture was quenched with water (30 mL) and extracted three times with DCM (30 mL each). The combined organic phases were washed with brine (30 mL), dried with magnesium sulfate and the solvent was removed under reduced pressure. FCC (iso-hexane/ethyl acetate = 17:3, R_f = 0.4) was performed for purification. Traces of tributyltin bromide were removed by dissolving the product in as little DCM as possible and precipitating it with iso-hexane. This resulted in 1.02 g (yield 75 %) of the pure target product as a white powder.

M.p.: 114 °C

^{1}H NMR (500 MHz, CD_2Cl_2) δ (ppm) = 7.93 – 7.88 (m, 2H, 2'-H, 6'-H), 7.47 – 7.43 (m, 2H, 3'-H, 5'-H), 7.12 (dd, *J* = 7.6, 1.6 Hz, 2H, 4'''-H, 6'''-H), 7.00 (ddd, *J* = 8.1, 7.4, 1.6 Hz, 2H, 2'''-H, 8'''-H), 6.89 (td, *J* = 7.5, 1.2 Hz, 2H, 3'''-H, 7'''-H), 6.65 (dd, *J* = 8.2, 1.2 Hz, 2H, 1'''-H, 9'''-H), 5.14 (s, 2H, 1''-H), 2.55 (s, 3H, 2-H)

^{13}C NMR (126 MHz, CD_2Cl_2) δ (ppm) = 197.8 (C-1), 145.0 (C-9a''', C-10a'''), 143.1 (C-4'), 136.8 (C-1'), 129.2 (C-2', C-6'), 127.8 (C-2''', C-8'''), 127.5 (C-3', C-5'), 127.5 (C-4''', C-6'''), 124.3 (C-4a''', C-5a'''), 123.3 (C-3''', C-7'''), 116.0 (C-1''', C-9'''), 52.7 (C-1''), 27.0 (C-2)

IR (ATR): $\tilde{\nu}$ (cm^{-1}) = 1670, 1605, 1588, 1567, 1462, 1442, 1410, 1363, 1256, 1225, 1127, 1106, 966, 862, 819, 756, 740, 643, 577

HRMS (ESI): m/z = 332.1107 $[M + H]^+$ calculated for $C_{21}H_{18}NOS^+$ 332.1109

Methyl 3-{4-[(10*H*-phenothiazin-10-yl)methyl]benzamido}isonicotinate (MM-19)

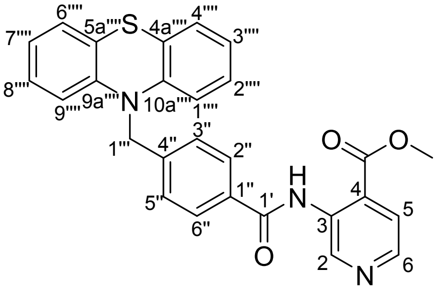

$C_{27}H_{21}N_3O_3S$

M_r = 467.54

A solution of 593 mg (1.78 mmol) 4-[(10*H*-phenothiazin-10-yl)methyl]benzoic acid[31] in 5.0 mL DCM was cooled to 0 °C. Then, 143 µL (1.78 mmol) pyridine and 129 µL (1.78 mmol) thionyl chloride were added at this temperature and the mixture stirred for 10 min. Afterwards, a solution of 226 mg (1.48 mmol) methyl 3-aminoisonicotinate and 576 µL (7.42 mmol) pyridine in 2.0 mL DCM was added. The mixture was stirred for 1.5 h, the ice bath was subsequently removed and stirring continued for another 19 h. The solvent was removed under reduced pressure, the reaction mixture diluted with saturated $NaHCO_3$ (16 mL) and extracted three times with a mixture of ethyl acetate and methanol (9:1, 30 mL each). The combined organic phases were dried with sodium sulfate and the solvent was removed under reduced pressure. Purification by FCC (iso-hexane/ethyl acetate = 1:1, R_f = 0.4) resulted in 192 mg (yield 28 %) of a yellow solid as product.

M.p.: 181 °C

^{1}H NMR (500 MHz, CD_2Cl_2) δ (ppm) = 11.56 (s, 1H, NH), 10.14 (s, 1H, 2-H), 8.45 (d, *J* = 4.7 Hz, 1H, 6-H), 8.03 – 7.95 (m, 2H, 2''-H, 6''-H), 7.84 (d, *J* = 5.0 Hz, 1H, 5-H), 7.56 – 7.49 (m, 2H, 3''-H, 5''-H), 7.13 (dd, *J* = 7.6, 1.6 Hz, 2H, 4''''-H, 6''''-H), 7.02 (ddd, *J* = 8.1, 7.4, 1.6 Hz, 2H, 2''''-H, 8''''-H), 6.90 (td, *J* = 7.5, 1.2 Hz, 2H, 3''''-H, 7''''-H), 6.68 (dd, *J* = 8.2, 1.2 Hz, 2H, 1''''-H, 9''''-H), 5.18 (s, 2H, 1'''-H), 3.98 (s, 3H, CO_2CH_3)

^{13}C NMR (126 MHz, CD_2Cl_2) δ (ppm) = 168.4 ($\underline{C}O_2CH_3$), 165.3 (C-1'), 145.0 (C-9a'''', C-10a''''), 144.5 (C-6), 144.0 (C-2), 142.4 (C-4''), 137.1 (C-3), 133.6 (C-1''), 128.4 (C-2'', C-6''), 127.9 (C-3'', C-5''), 127.8 , (C-2'''', C-8''''), 127.5 (C-4'''', C-6''''), 124.2 (C-4a'''', C-5a''''), 123.3 (C-3'''', C-7''''), 123.2 (C-5), 121.5 (C-4), 116.0 (C-1'''', C-9''''), 53.6 , ($CO_2\underline{CH_3}$), 52.6 (C-1''')

IR (ATR): $\tilde{\nu}$ (cm^{-1}) = 1701, 1677, 1595, 1571, 1524, 1502, 1466, 1439, 1415, 1366, 1293, 1261, 1179, 1105, 1051, 1017, 959, 868, 792, 741, 705, 971

HRMS (ESI): m/z = 468.1380 $[M-H]^+$ calculated for $C_{27}H_{22}N_3O_3S^+$ 468.1382

3-{4-[(10*H*-Phenothiazin-10-yl)methyl]benzamido}isonicotinic acid (MM-21)

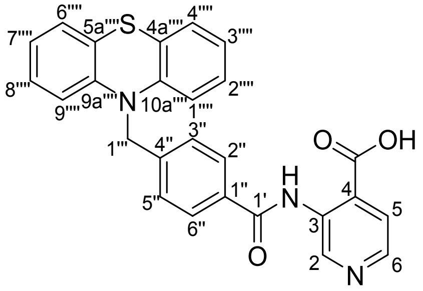

$C_{26}H_{19}N_3O_3S$

M_r = 453.52

To a suspension of 140 mg (0.319 mmol) methyl 3-{4-[(10*H*-phenothiazin-10-yl)methyl]benzamido}isonicotinate (**MM-19**) in 2 mL of a mixture of water/1,4-dioxane (1:1) 0.20 mL (0.40 mmol) 2 M NaOH were added. The mixture was heated to 60 °C and stirred for 19 h. Then, the mixture was cooled to room temperature and acidified with 0.24 mL (0.48 mmol) 2 M HCl to a pH of about 2. The precipitated carboxylic acid was collected by filtration and residual 1,4-dioxane and water were removed by suspending the compound in 5.0 mL distilled acetone. The mixture was centrifuged and the supernatant was discarded. This procedure was repeated with acetone another four times and with distilled DCM another three times. The highly insoluble title compound was thus obtained completely pure as a white solid (94.1 mg, yield 65 %).

M.p.: 267 - 269 °C (under decomposition)

^{1}H NMR (80 °C, 400 MHz, DMSO-*d$_6$*) δ (ppm) = 11.41 (s, 1H, NH), 9.67 (s, 1H, 2-H), 8.46 (d, *J* = 5.0 Hz, 1H, 6-H), 7.98 – 7.87 (m, 2H, 2''-H, 6''-H), 7.83 (d, *J* = 5.0 Hz, 1H, 5-H), 7.62 – 7.50 (m, 2H, 3''-H, 5''-H), 7.15 (dd, *J* = 7.6, 1.6 Hz, 2H, 4''''-H, 6''''-H), 7.08 (td, *J* = 7.7, 1.6 Hz, 2H, 2''''-H, 8''''-H), 6.92 (td, *J* = 7.5, 1.2 Hz, 2H, 3''''-H, 7''''-H), 6.84 (dd, *J* = 8.0, 1.2 Hz, 2H, 1''''-H, 9''''-H), 5.25 (s, 2H, 1'''-H)

^{13}C NMR (80 °C, 101 MHz, DMSO-*d$_6$*) δ (ppm) = 167.4 (COOH), 164.2 (C-1'), 143.9 (C-6), 143.9 (C-9a'''', C-10a''''), 143.0 (C-2), 141.6 (C-4''), 134.8 (C-3), 132.4 (C-1''), 127.1 (C-2'', C-6''), 127.0 (C-2'''', C-8''''), 126.9 (C-3'', C-5''), 126.4 (C-4'''', C-6''''), 125.4 (C-4), 122.9 (C-5), 122.7 (C-4a'''', C-5a''''), 122.3 (C-3'''', C-7''''), 115.5 (C-1'''', C-9''''), 50.6 (C-1''')

IR (ATR): $\tilde{\nu}$ (cm^{-1}) = 1683, 1573, 1509, 1456, 1441, 1336, 1287, 1255, 1222, 1181, 1089, 1042, 935, 870, 840, 804, 746, 653

HRMS (ESI): m/z = 454.1223 [M + H]$^+$ calculated for $C_{26}H_{20}N_3O_3S^+$ 454.1225

HPLC purity: not soluble in mobile phase

1-{4-[(10*H*-Phenothiazin-10-yl)methyl]phenyl}-2,2,2-trifluoroethan-1-one (MM-22)

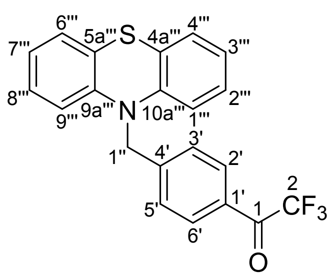

$C_{21}H_{14}F_3NOS$

M_r = 385.40

Method a)

A solution of 1.07 g (3.09 mmol) methyl-4-[(10*H*-phenothiazin-10-yl)methyl]benzoate[31] in 50 mL dry toluene under N_2 atmosphere was cooled to -78 °C. Then, 0.638 mL (4.32 mmol) trimethyl(trifluoromethyl)silane (Ruppert's reagent) were added and the mixture was stirred for 15 min followed by adding 0.247 mL (0.247 mmol) tetrabutylammonium fluoride (1 M in THF). The cooling bath was subsequently removed and the mixture was stirred for 27 h. Next, the mixture was treated with 20.0 mL (40.0 mmol) 2 M HCl and stirred for 2 h at room temperature. The organic layer was separated and the aqueous phase extracted once with diethyl ether (30 mL). The combined organic phases were dried with magnesium sulfate and the solvent was removed under reduced pressure. FCC was performed (iso-hexane/DCM = 1:1, R_f = 0.5 - 0.7 and iso-hexane/acetone = 17:3, R_f = 0.1 - 0.3) to yield the title compound as a yellow oil with 693 mg in 79 % purity (547.2 mg, 1.42 mmol, 46 %).

Method b)

A solution of 880 mg (2.39 mmol) 10-(4-bromobenzyl)-10*H*-phenothiazine (**MM-13**) in 12 mL dry THF under N_2 atmosphere was cooled down to -78 °C with dry ice in acetone. At this temperature, 1.15 mL (2.86 mmol) *n*-butyllithium (2.5 M in hexane) were added over 50 min and the mixture was stirred for 75 min. Afterwards, a solution of 0.458 mL (3.25 mmol) *N,N*-diethyl-2,2,2-trifluoroacetamide in 2.0 mL dry THF was added over 5 min and the mixture stirred for 3.5 h. At -78 °C, the reaction mixture was quenched with a saturated NH_4Cl solution (30 mL), and the mixture was extracted two times with ethyl acetate (25 mL each). The combined organic phases were washed two times with a saturated NH_4Cl solution (25 mL each) and two times with water (20 mL each). The crude mixture was purified with FCC (iso-hexane/acetone = 17:3, R_f = 0.1 - 0.3) to obtain the title compound as a yellow oil with 490 mg in 79 % purity (387 mg, 1.00 mmol, 42 %).

^{1}H NMR (500 MHz, CD_2Cl_2) δ (ppm) = 8.08 – 8.00 (m, 2H, 2'-H, 6'-H), 7.62 – 7.55 (m, 2H, 3'-H, 5'-H), 7.14 (dd, *J* = 7.6, 1.6 Hz, 2H, 4'''-H, 6'''-H), 7.02 (ddd, *J* = 8.0, 7.3, 1.5 Hz, 2H, 2'''-H, 8'''-H), 6.91 (td, *J* = 7.5, 1.2 Hz, 2H, 3'''-H, 7'''-H), 6.63 (dd, *J* = 8.2, 1.1 Hz, 2H, 1'''-H, 9'''-H), 5.18 (s, 2H, 1''-H)

^{13}C NMR (126 MHz, CD_2Cl_2) δ (ppm) = 180.5 (q, $^2J_{C,F}$ = 34.9 Hz, C-1), 146.5 (C-4'), 144.8 (C-9a''', C-10a'''), 131.1 (q, $^4J_{C,F}$ = 2.2 Hz, C-2', C-6'), 129.4 (C-1'), 128.2 (C-3', C-5'), 127.9 (C-2''', C-8'''), 127.6 (C-4''', C-6'''), 124.5 (C-4a''', C-5a'''), 123.4 (C-3''', C-7'''), 117.3 (q, $^1J_{C,F}$ = 291.4 Hz, C-2), 116.0 (C-1''', C-9'''), 52.7 (C-1'')

IR (ATR): $\tilde{\nu}$ (cm^{-1}) = 3066, 2925, 2854, 1804, 1714, 1613, 1594, 1574, 1466, 1367, 1286, 1258, 1206, 1170, 1148, 939, 867, 752

HRMS (ESI): m/z = 384.0676 [M - H]$^-$ calculated for $C_{21}H_{13}F_3NOS^-$ 384.0670

HPLC purity: 78% (λ = 210 nm), 79% (λ = 254 nm)

(*E*)-1-{4-[(10*H*-Phenothiazin-10-yl)methyl]phenyl}-2,2,2-trifluoroethan-1-one oxime (MM-24)

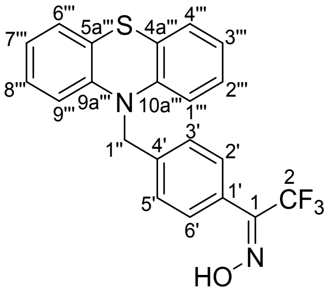

$C_{21}H_{15}F_3N_2OS$

M_r = 400.42

A solution of 239 mg (0.619 mmol) 1-{4-[(10*H*-phenothiazin-10-yl)methyl]phenyl}-2,2,2-trifluoroethan-1-one (**MM-22**), 86.0 mg (1.24 mmol) hydroxylamine hydrochloride and 74.8 µL (0.928 mmol) pyridine in 2 mL methanol was refluxed for 5 h. After cooling, the reaction mixture was quenched with water (30 mL) and extracted three times with DCM (30 mL each). The combined organic phases were dried with magnesium sulfate and the solvent was removed under reduced pressure. Purification by FCC (iso-hexane/DCM = 3:7, R_f = 0.2) resulted in 189 mg (yield 76 %) of a pink solid as product.

M.p.: 125 - 126 °C

^{1}H NMR (400 MHz, CD_2Cl_2) δ (ppm) = 8.32 (s, 1H, OH), 7.52 – 7.44 (m, 4H, 2'-H, 3'-H, 5'-H, 6'-H), 7.12 (dd, *J* = 7.5, 1.6 Hz, 2H, 4'''-H, 6'''-H), 7.02 (ddd, *J* = 8.2, 7.4, 1.6 Hz, 2H, 2'''-H, 8'''-H), 6.90 (td, *J* = 7.5, 1.2 Hz, 2H, 3'''-H, 7'''-H), 6.67 (dd, *J* = 8.2, 1.2 Hz, 2H, 1'''-H, 9'''-H), 5.14 (s, 2H, 1''-H)

^{13}C NMR (101 MHz, CD_2Cl_2) δ (ppm) = 148.0 (q, *J* = 32.4 Hz, C-1), 145.0 (C-9a''', C-10a'''), 140.5 (C-4'), 129.6 (C-2', C-6'), 127.8 (C-2''', C-8'''), 127.5 (C-3', C-5'), 127.5 (C-4''', C-6'''), 125.3 (C-1'), 124.1 (C-4a''', C-5a'''), 123.3 (C-3''', C-7'''), 118.5 (q, *J* = 274.5 Hz, C-2), 116.0 (C-1''', C-9'''), 52.7 (C-1'')

IR (ATR): $\tilde{\nu}$ (cm^{-1}) = 3312, 2922, 1596, 1569, 1489, 1460, 1443, 1368, 1260, 1221, 1187, 1139, 1032, 1010, 961, 864, 750, 735

HRMS (ESI): m/z = 401.0932 [M-H]$^+$ calculated for $C_{21}H_{16}F_3N_2OS^+$ 401.0935

Methyl {4-[(10*H*-phenothiazin-10-yl)methyl]benzoyl}-L-prolinate (MM-25)

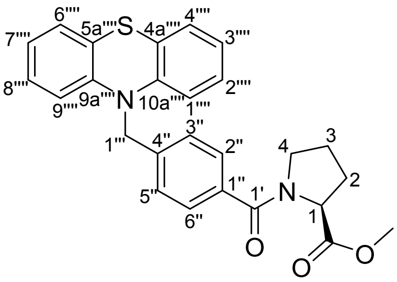

$C_{26}H_{24}N_2O_3S$

M_r = 444.55

Under N_2 atmosphere, 229 mg (0.687 mmol) 4-[(10*H*-phenothiazin-10-yl)methyl]benzoic acid[31] were dissolved in 3.0 mL dry DMF at 0 °C and activated by adding 294 mg (0.687 mmol) COMU and 0.12 mL (0.69 mmol) DIPEA and stirring the mixture for 5 min. Afterwards, 114 mg (0.687 mmol) L-proline methyl ester and 0.12 mL (0.69 mmol) DIPEA were added and the mixture was stirred for 10 min at 0 °C. Next, the ice bath was removed and stirring continued for another 21 h. The reaction mixture was quenched with ice water (30 mL) and extracted three times with DCM (20 mL each). The combined organic fractions were washed two times with saturated $NaHCO_3$ (20 mL each) and once with brine (20 mL), dried with sodium sulfate and the solvent was removed under reduced pressure. Purification by FCC (iso-hexane/ethyl acetate = 1:1, R_f = 0.2) resulted in 245 mg (yield 80 %) of a white, fine powder as product.

M.p.: 153 °C

^{1}H NMR (500 MHz, CD_2Cl_2) δ (ppm) = 7.54 – 7.49 (m, 2H, 2''-H, 6''-H), 7.41 – 7.36 (m, 2H, 3''-H, 5''-H), 7.10 (dd, *J* = 7.6, 1.6 Hz, 2H, 4''''-H, 6''''-H), 7.00 (ddd, *J* = 8.1, 7.4, 1.6 Hz, 2H, 2''''-H, 8''''-H), 6.88 (td, *J* = 7.5, 1.2 Hz, 2H, 3''''-H, 7''''-H), 6.66 (dd, *J* = 8.3, 1.2 Hz, 2H, 1''''-H, 9''''-H), 5.13 (s, 2H, 1'''-H), 4.57 (dd, *J* = 8.4, 5.0 Hz, 1H, 1-H), 3.73 (s, 3H, CO_2CH_3), 3.65 – 3.57 (m, 1H, 4-H_a/4-H_e), 3.56 – 3.49 (m, 1H, 4-H_a/4-H_e), 2.35 – 2.25 (m, 1H, 2-H_a/2-H_e), 2.06 – 1.92 (m, 2H, 2-H_a/2-H_e, 3-H_a/3-H_e), 1.92 – 1.80 (m, 1H, 3-H_a/3-H_e)

^{13}C NMR (126 MHz, CD_2Cl_2) δ (ppm) = 173.2 ($\underline{C}O_2CH_3$), 169.4 (C-1'), 145.0 (C-9a'''', C-10a''''), 139.8 (C-4''), 135.7 (C-1''), 128.2 (C-2'', C-6''), 127.8 (C-2'''', C-8''''), 127.4 (C-4'''', C-6''''), 127.1 (C-3'', C-5''), 124.0 (C-4a'''', C-5a''''), 123.2 (C-3'''', C-7''''), 116.1 (C-1'''', C-9''''), 59.8 (C-1), 52.8 (C-1'''), 52.6 ($CO_2\underline{C}H_3$), 50.5 (C-4), 29.9 (C-2), 26.0 (C-3).

IR (ATR): $\tilde{\nu}$ (cm^{-1}) = 1742, 1629, 1570, 1464, 1419, 1364, 1284, 1255, 1200, 1173, 1038, 1022, 925, 864, 749

HRMS (ESI): m/z = 445.1579 $[M\text{-}H]^+$ calculated for $C_{26}H_{25}N_2O_3S^+$ 445.1586

1-{4-[(10*H*-Phenothiazin-10-yl)methyl]phenyl}-2-nitroethan-1-one (MM-27)

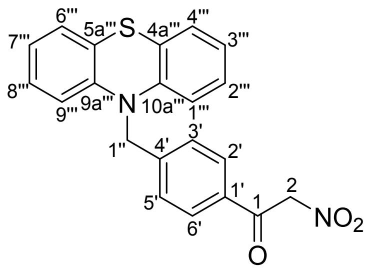

$C_{21}H_{16}N_2O_3S$

M_r = 376.43

Activation of the carboxylic acid: A solution of 498 mg (1.49 mmol) 4-[(10*H*-phenothiazin-10-yl)methyl]benzoic acid[31] and 363 mg (2.24 mmol) 1,1'-carbonyldiimidazole in 12 mL dry THF under N_2 atmosphere was heated to reflux and stirred for 1 h.

To a suspension of 252 mg (2.24 mmol) potassium *tert*-butoxide in 10 mL dry THF under N_2 atmosphere at 0 °C 120 µL (2.24 mmol) anhydrous nitromethane were added over 5 min and the mixture stirred for completion of the deprotonation for 2 h. After letting the reaction mixture cool to room temperature, the solution of the activated carboxylic acid was slowly added to the deprotonated nitromethane over 4 min. The mixture was stirred at 0 °C for 10 min, at room temperature for 40 min and refluxed for 12 h. Within this period, the activated carboxylic acid was not completely consumed as confirmed by TLC monitoring, therefore another mixture of potassium *tert*-butoxide (503 mg, 4.48 mmol) and nitromethane (240 µL, 4.48 mmol) in anhydrous THF (10 mL) was prepared and stirred for 1 h. The cooled down reaction mixture was added to the second nitromethane solution at 0 °C and after stirring at room temperature for 4 h, the solution was refluxed for 2.5 h. After cooling the mixture, 15 mL 1 M HCl was added until pH 1 was established. Next, the mixture was extracted three times with ethyl acetate (25 mL each). The combined organic fractions were washed with brine (30 mL), dried over sodium sulfate and the solvent was removed under reduced pressure. Purification by FCC (iso-hexane/DCM = 2:3 with 0.1 % acetic acid, R_f = 0.4) resulted in 372 mg (yield 66 %) of a yellow solid as product.

M.p.: 79 - 82 °C

^{1}H NMR (400 MHz, CD_2Cl_2) δ (ppm) = 7.86 – 7.80 (m, 2H, 2'-H, 6'-H), 7.57 – 7.52 (m, 2H, 3'-H, 5'-H), 7.13 (ddd, *J* = 7.6, 1.6, 0.4 Hz, 2H, 4'''-H, 6'''-H), 7.02 (ddd, *J* = 8.2, 7.4, 1.6 Hz, 2H, 2'''-H, 8'''-H), 6.90 (td, *J* = 7.5, 1.2 Hz, 2H, 3'''-H, 7'''-H), 6.63 (ddd, *J* = 8.2, 1.2, 0.4 Hz, 2H, 1'''-H, 9'''-H), 5.89 (s, 2H, 2-H), 5.17 (s, 2H, 1''-H).

^{13}C NMR (101 MHz, CD_2Cl_2) δ (ppm) = 186.1 (C-1), 145.8 (C-4'), 144.9 (C-9a''', C-10a'''), 133.0 (C-1'), 129.2 (C-2', C-6'), 128.3 (C-3', C-5'), 127.8 (C-2''', C-8'''), 127.6 (C-4''', C-6'''), 124.5 (C-4a''', C-5a'''), 123.4 (C-3''', C-7'''), 116.0 (C-1''', C-9'''), 82.2 (C-2), 52.6 (C-1'').

IR (ATR): $\tilde{\nu}$ (cm^{-1}) = 1699, 1605, 1560, 1463, 1366, 1324, 1256, 1226, 995, 863, 750, 653

HRMS (ESI): m/z = 375.0810 $[M-H]^-$ calculated for $C_{21}H_{15}N_2O_3S^-$ 375.0803

Supporting References

1. Lechner, S. *et al.* Target deconvolution of HDAC pharmacopoeia reveals MBLAC2 as common off-target. *Nat Chem Biol* **18**, 812–820 (2022).

2. Reinecke, M. *et al.* Chemoproteomic Selectivity Profiling of PIKK and PI3K Kinase Inhibitors. *ACS Chem Biol* **14**, 655–664 (2019).

3. Fuchs, H. *et al.* Understanding gene functions and disease mechanisms: Phenotyping pipelines in the German Mouse Clinic. *Behavioural Brain Research* **352**, 187–196 (2018).

4. Fuchs, H. *et al.* Mouse phenotyping. *Methods* **53**, 120–135 (2011).

5. Gailus-Durner, V. *et al.* Introducing the German Mouse Clinic: open access platform for standardized phenotyping. *Nat Methods* **2**, 403–404 (2005).

6. Rathkolb, B. *et al.* Blood Collection from Mice and Hematological Analyses on Mouse Blood. *Curr Protoc Mouse Biol* **3**, 101–119 (2013).

7. Rathkolb, B. *et al.* Clinical Chemistry and Other Laboratory Tests on Mouse Plasma or Serum. *Curr Protoc Mouse Biol* **3**, 69–100 (2013).

8. Hölter, S. M. *et al.* Tests for Anxiety-Related Behavior in Mice. *Curr Protoc Mouse Biol* **5**, 291–309 (2015).

9. Heermann, T. *et al.* Crybb2 Mutations Consistently Affect Schizophrenia Endophenotypes in Mice. *Mol Neurobiol* **56**, 4215–4230 (2019).

10. Pawliczek, D. *et al.* Spectral domain - Optical coherence tomography (SD-OCT) as a monitoring tool for alterations in mouse lenses. *Exp Eye Res* **190**, 107871 (2020).

11. Moreth, K. *et al.* High-throughput phenotypic assessment of cardiac physiology in four commonly used inbred mouse strains. *Journal of Comparative Physiology B* **184**, 763–775 (2014).

12. Hughes, C. S. *et al.* Single-pot, solid-phase-enhanced sample preparation for proteomics experiments. *Nat Protoc* **14**, 68–85 (2019).

13. Ruprecht, B., Zecha, J., Zolg, D. P. & Kuster, B. *High PH Reversed-Phase Micro-Columns for Simple, Sensitive, and Efficient Fractionation of Proteome and (TMT Labeled) Phosphoproteome Digests*. vol. 1550 (Springer New York, New York, NY, 2017).

14. Tüshaus, J. *et al.* A region-resolved proteomic map of the human brain enabled by high-throughput proteomics. *EMBO J* **42**, (2023).

15. Eberhardt, J., Santos-Martins, D., Tillack, A. F. & Forli, S. AutoDock Vina 1.2.0: New Docking Methods, Expanded Force Field, and Python Bindings. *J Chem Inf Model* **61**, 3891–3898 (2021).

16. Morris, G. M. *et al.* AutoDock4 and AutoDockTools4: Automated docking with selective receptor flexibility. *J Comput Chem* **30**, 2785–2791 (2009).

17. Salentin, S., Schreiber, S., Haupt, V. J., Adasme, M. F. & Schroeder, M. PLIP: fully automated protein–ligand interaction profiler. *Nucleic Acids Res* **43**, W443–W447 (2015).

18. Evans, P. R. & Murshudov, G. N. How good are my data and what is the resolution? *Acta Crystallogr D Biol Crystallogr* **69**, 1204–1214 (2013).

19. Dolot, R. *et al.* Biochemical, crystallographic and biophysical characterization of histidine triad nucleotide-binding protein 2 with different ligands including a non-hydrolyzable analog of Ap4A. *Biochim Biophys Acta Gen Subj* **1865**, (2021).

20. Vagin, A. & Teplyakov, A. MOLREP: an Automated Program for Molecular Replacement. *J Appl Crystallogr* **30**, 1022–1025 (1997).

21. Emsley, P. & Cowtan, K. Coot: model-building tools for molecular graphics. *Acta Crystallogr D Biol Crystallogr* **60**, 2126–2132 (2004).

22. Lebedev, A. A. *et al.* JLigand: a graphical tool for the CCP4 template-restraint library. *Acta Crystallogr D Biol Crystallogr* **68**, 431–440 (2012).

23. Kovalevskiy, O., Nicholls, R. A., Long, F., Carlon, A. & Murshudov, G. N. Overview of refinement procedures within REFMAC5: utilizing data from different sources. *Acta Crystallogr D Struct Biol* **74**, 215–227 (2018).

24. Chen, V. B. *et al.* MolProbity: all-atom structure validation for macromolecular crystallography. *Acta Crystallogr D Biol Crystallogr* **66**, 12–21 (2010).

25. Winn, M. D. *et al.* Overview of the CCP4 suite and current developments. *Acta Crystallogr D Biol Crystallogr* **67**, 235–242 (2011).

26. Kabsch, W. & Sander, C. Dictionary of protein secondary structure: Pattern recognition of hydrogen-bonded and geometrical features. *Biopolymers* **22**, 2577–2637 (1983).

27. Yue, K. *et al.* First-in-Class Hydrazide-Based HDAC6 Selective Inhibitor with Potent Oral Anti-Inflammatory Activity by Attenuating NLRP3 Inflammasome Activation. *J Med Chem* **65**, 12140–12162 (2022).

28. Deziel, R. *et al.* Dibenzo[b,f][1,4]oxazepine derivatives as inhibitors of histone deacetylase . EP 2 343 286 A1.

29. Katsilometes, G. C. *et al.* Novel 10, 10'-substituted-9,9'-biacridine luminescent molecules and their preparation . EP 1 026 156 A2.

30. Bay, S., Makhloufi, G., Janiak, C. & Müller, T. J. J. The Ugi four-component reaction as a concise modular synthetic tool for photo-induced electron transfer donor-anthraquinone dyads. *Beilstein Journal of Organic Chemistry* **10**, 1006–1016 (2014).

31. Vögerl, K. *et al.* Synthesis and Biological Investigation of Phenothiazine-Based Benzhydroxamic Acids as Selective Histone Deacetylase 6 Inhibitors. *J Med Chem* **62**, 1138–1166 (2019).
